# Substandard and falsified antibiotics: neglected drivers of antimicrobial resistance?

**DOI:** 10.1136/bmjgh-2022-008587

**Published:** 2022-08-18

**Authors:** Guillermo A Zabala, Khonsavath Bellingham, Vayouly Vidhamaly, Phonepasith Boupha, Kem Boutsamay, Paul N Newton, Céline Caillet

**Affiliations:** 1 Lao-Oxford-Mahosot Hospital-Wellcome Trust Research Unit, Medicine Quality Research Group, Laboratory of Microbiology, Mahosot Hospital, Vientiane, Lao People's Democratic Republic; 2 Nuffield Department of Medicine, Medicine Quality Research Group, University of Oxford Centre for Tropical Medicine and Global Health, Oxford, UK; 3 Clinical Infection Unit, Saint George's University Hospital NHS Foundation Trust, London, UK; 4 Nuffield Department of Medicine, Infectious Diseases Data Observatory (IDDO)/WorldWide Antimalarial Resistance Network (WWARN), Medicine Quality Research Group, University of Oxford, Oxford, UK

**Keywords:** medical microbiology, public Health, pharmacology

## Abstract

**Objectives:**

Antimicrobial resistance (AMR) is a significant global health threat with substandard and falsified (SF) antibiotics being neglected contributing factors. With their relationships poorly understood, more research is needed in order to determine how interventions to reduce SF antibiotics should be ranked as priorities in national AMR action plans. We assessed the evidence available on the global prevalence of SF antibiotics, examined the quality of the evidence and discussed public health impact.

**Materials/Methods:**

We searched PubMed, Embase, Google and Google Scholar for publications on antibiotic quality up to 31 December 2020. Publications reporting on the prevalence of SF antibiotics were evaluated for quantitative analysis and assessed using the Medicines Quality Assessment Reporting Guidelines.

**Results:**

Of the 10 137 screened publications, 648 were relevant to antibiotic quality. One hundred and six (16.4%) surveys, published between 1992 and 2020 and conducted mainly in low-income and middle-income countries (LMICs) (89.9% (480/534) of the data points), qualified for quantitative analysis. The total number of samples tested for quality in prevalence surveys was 13 555, with a median (Q1–Q3) number of samples per survey of 47 (21–135). Of the 13 555 samples, 2357 (17.4%) failed at least one quality test and the median failure frequency (FF) per survey was 19.6% (7.6%–35.0%). Amoxicillin, sulfamethoxazole-trimethoprim and ciprofloxacin were the most surveyed antibiotics, with FF of 16.1% (355/2208), 26.2% (329/1255) and 10.4% (366/3511), respectively. We identified no SF survey data for antibiotics in the WHO ‘Reserve’ group. The mean Medicine Quality Assessment Reporting Guidelines score was 11 (95% CI 10.1 to 12.2) out of 26.

**Conclusions:**

SF antibiotics are widely spread with higher prevalence in LMICs. The quality of the evidence is poor, and these data are not generalisable that 17.4% of global antibiotic supply is SF. However, the evidence we have suggests that interventions to enhance regulatory, purchasing and financial mechanisms to improve the global antibiotic supply are needed.

**PROSPERO registration number:**

CRD42019124988.

**WHAT IS ALREADY KNOWN ON THIS TOPIC**

- Substandard and falsified (SF) antibiotics worsen clinical outcomes, lead to adverse drug reactions, economic loss and diminish public confidence in health systems, but there is limited evidence on their prevalence, although they are also hypothesised to be locally key drivers of antimicrobial resistance (AMR).

**WHAT THIS STUDY ADDS**

- One hundred and six prevalence surveys were identified, including a total of 13 555 samples, and 17.4% of those failed at least one quality test.
- Samples mainly failed because they did not contain the correct amount of active pharmaceutical ingredient or failed dissolution testing, risking reduced bioavailability.
- There are major gaps in the evidence, with geographical disparities, and no data for many antibiotics important for public health and AMR.
- These data are not generalisable to suggest that 17.4% of the global antibiotic supply are SF.

**HOW THIS STUDY MIGHT AFFECT RESEARCH, PRACTICE OR POLICY**

- The quality of antibiotics, especially in supply chains in countries without stringent medicine regulation, requires urgent attention.
- Further research is needed to assess the quality of antibiotics worldwide and its link to AMR, to inform policy to combat this serious health threat.

## Introduction

Eight months after evidence supporting sulfanilamide use as an antibiotic in the USA in 1937, at least 105 patients died due to a toxic excipient.^1^ This disaster led directly to the strengthening of the US Food and Drug Administration and requirement for evidence of safety before new medicine approval. The following decades saw the rapid spread of antibiotic use globally and an increasing number of agents available. The antibiotic market reached a total value of ~US$45 billion in 2018.^2^

Bacterial pathogens are still killing people in large numbers, with higher burdens in low-income and middle-income countries (LMICs).^3^ With global economic development, antibiotic demand and consumption are growing; between 2000 and 2015, global antimicrobial medicines consumption (AMC) increased by 65% and is projected to increase up to 200% by 2030.^4 5^ Compromised access is compounded by substandard and falsified (SF) antibiotics. Falsified medicines are those that ‘deliberately/fraudulently misrepresent their identity, composition or source’.^5^ Substandard medicines are ‘authorised medical products that fail to meet either their quality standards or their specifications, or both’.^5^ These may result from gross negligence/unintended errors during the manufacturing process or degradation through inappropriate storage/transport within the supply chain. Both may contain low, high, no or wrong active ingredients or impaired dissolution and can prolong illness duration and lead to death, loss of income, increased spending on healthcare and sow public mistrust.^6^

Within 5 years of its first clinical use, a large trade in falsified penicillin developed.^7^ The problems persist; recently, a child’s death in Uganda was associated with substandard ceftriaxone containing <50% of the stated active pharmaceutical ingredient (API).^8^ WHO estimated that the prevalence of SF medicines is ~10% in LMICs, and antibiotics are the second most frequent class of SF medical products reported to the WHO Global Surveillance and Monitoring System.^9^ Mathematical modelling suggested that 72 430 childhood pneumonia deaths per year are attributable to SF antibiotics.^10^

Subtherapeutic antibiotic concentrations promote antimicrobial resistance (AMR),^11–13^ and one of the likely mechanisms for this is the consumption of SF medicines containing lower than the stated antibiotic concentrations, poor dissolution and/or adulteration with subtherapeutic amounts of unstated, cryptic, antibiotics.^14–18^ For example, the high prevalence of substandard chloramphenicol and sulfamethoxazole-trimethoprim in Myanmar may have contributed to the high typhoid antibiotic resistance prevalence.^19 20^

Understanding the relationship between the prevalence of SF medicines and AMR using field data is impaired because of poorly understood time intervals between bacterial exposure to antibiotics and rise in AMR prevalence. Also, areas with high SF antibiotic prevalence are also likely to have other sympatric AMR drivers such as poor patient adherence, antibiotic misuse and inappropriate prescriptions, confounding relationships.^21 22^ The WHO AWaRe (Access, Watch, Reserve) classification helps optimise antibiotic use and guides policies on access to quality antibiotics as contributors to Universal Health Coverage and the Sustainable Development Goals to reduce the risk of AMR.^23^

Research on the impact of SF antibiotics on AMR is neglected despite multiple passing references,^24 25^ with AMR studies commonly omitting SF antibiotics as potential drivers.^22 26–36^ Here, we review the epidemiology of SF antibiotics, discuss their potential impact on AMR and provide evidence to inform interventions.

## Methods

### Search strategy

PubMed and Embase databases were searched in English, and Google and Google Scholar searched in English and French, without an inclusion start date, up to 31 December 2020. Search terms included the WHO terminology for medicine quality and other commonly used terms (eg, “substandard”, “falsified”, “counterfeit”) and all antibiotic API listed in the Anatomical Therapeutic Chemical classification.^5 9 37^ Different spelling and naming variations were included, such as British approved names (BAN) and alternative spellings (eg, cephalexin and cefalexin). Google and Google Scholar search terms were adapted for the character limit (online supplemental file 1). The results from PubMed, Embase and the first 20 pages (200 reports) of Google Scholar and Google were exported to Mendeley Desktop citation manager. After removal of duplicates, titles and abstracts were screened and full texts of the identified articles were assessed for eligibility. Manual searches of the reference lists in the included articles, and of the websites of Medicines Regulatory Agencies (MRA) and other organisations involved in medicine quality were performed. Relevant articles discovered in previous work by our group, but not captured by our search, were also included. Articles in Spanish and French resulting from our search were also included after translation by native speakers. This review was registered with the National Institute for Health Research International Prospective Register of Systematic Reviews (PROSPERO) (registration number: CRD42019124988).

10.1136/bmjgh-2022-008587.supp1 Supplementary data

### Eligibility criteria

Scientific and grey literature articles evaluating or discussing the quality of antibiotics in English, Spanish or French, irrespective of whether they contained empirical data or not, were included. General discussions (eg, on the regulatory framework surrounding SF antibiotics) and reviews of the literature on various aspects of antibiotics’ quality (eg, review of the literature on antibiotic recalls) were included. We included studies that surveyed the quality of antibiotics in one or more locations (hereafter ‘prevalence surveys’), that compared the pharmaceutical equivalence of different brands of a given antibiotic (hereafter ‘equivalence studies’), reports of adverse events that brought into question the quality of the antibiotics used, studies describing assay techniques to determine the quality of antibiotics (hereafter ‘analytical technique studies’), publications discussing sampling methodology and pharmaceutical legislation, reports on seizures and recalls by pharmaceutical companies or MRA and case reports on antibiotic quality in the scientific or lay press (online supplemental file 2).

10.1136/bmjgh-2022-008587.supp2 Supplementary data

Antibiotics which are primarily used for the treatment of tuberculosis (rifampicin, isoniazid, ethambutol, pyrazinamide, bedaquiline, pretomanid, ethionamide, prothionamide, cycloserine and para-aminosalicylic acid) were not included. For the quantitative analysis, only publications that provided data on results for chemical and/or physical quality test results were included.

### Definitions

Further to the discussion of key definitions given in the ‘Introduction’ section,^9^ evidence to distinguish poor quality medicines resulting from errors within the factory or subsequent degradation in the supply chain due to heat, humidity or light exposure is sparse. It is not possible to accurately classify a medicine as substandard or falsified without packaging analysis. Products that failed at least one quality testing without information on packaging authenticity are thus defined as ‘substandard or falsified’ (SorF).^38^ This term is not mandated by the WHO definitions, but we suggest that it enhances understanding for samples with incomplete evidence. Samples without packaging analysis that contained an API other than the stated or no API were assumed to be falsified. There is a risk of misclassification of such samples as falsified when they are substandard due to severe manufacturing errors.

Pharmaceutical analysis relies on compendial tests described in pharmacopoeial monographs. For finished medicines, monographs commonly include the identification and quantification of API content (using sophisticated standardised techniques such as liquid chromatography coupled with various detectors), dissolution testing, detection of specific levels of predetermined impurities/related substances, uniformity of dosage units and additional attributes depending on the formulation of the product (eg, tablet friability). In many studies included here, not all pharmacopoeial analyses were conducted and a variety of non-pharmacopoeial assays were used, for example, for investigating specific contaminants or unstated APIs. Assay details were not always provided making it difficult to standardise the definition of a ‘failed sample’. Consequently, we define a failed sample as one for which at least one quality analysis test performed by the investigators gave a fail result, irrespective of the number and type of assays used. We relied on the authors conclusions as to whether a sample was genuine, falsified/counterfeit and substandard and did not reinterpret them.

We define ‘failure frequency’ (FF) as the proportion of samples included in a prevalence survey that failed at least one quality test described in the report. A ‘data point’ is a specific location where medicines were collected for quality analysis, at a given time during a given study (online supplemental file 2).

### Data collection

Information within each publication was manually extracted into the ‘Online Medicine Quality Data Manager’, an online database developed by the Infectious Diseases Data Observatory (IDDO) Informatics team and the Medicine Quality Research Group (MQRG). Publication type (eg, report, original research article), year of publication, publisher, sampling type, location (country and city, where available) and type of outlet where samples were collected, total number of samples collected, API/API combination name, number of samples failing medicine quality test(s), quality defect and the techniques used to analyse samples were entered. In stability studies, only data on the quality test results of medicines before being submitted to stress conditions were included. In cases where the threshold used for the consideration of the sample as ‘pass’ or ‘fail’ was unclear, we did not include the data in the analysis.

### Analysis and reporting

Data were extracted using FlySpeed SQL Query (V.3.5.4.2) and Microsoft Excel 365 and RStudio V.0.99.486 were used for data analysis and creation of figures and tables, including polynomial trend lines. Statistical analyses were carried out using Microsoft Excel 365 (means, medians and quartiles), FFs and Stata V.17.0. Qualitative variables were expressed as numbers and percentages (n (%)). Quantitative variables were expressed as the median and first and third quartiles (Q1–Q3) or mean (95% CI) where appropriate. This review followed the Preferred Reporting Items for Systematic Reviews and Meta-Analyses guidelines (online supplemental file 3).

10.1136/bmjgh-2022-008587.supp3 Supplementary data

### Risk of bias assessment

Articles in which the primary objective was to estimate the prevalence of SF antibiotics were reviewed using the Medicine Quality Assessment Reporting Guidelines (MEDQUARG).^39^ Only the prevalence surveys published as original articles in scientific journals or following the Introduction/Methods/Results/Discussion or similar style and published as reports, MSc or PhD thesis, were assessed. Two reviewers (GZ, KBe) blinded to each other’s scores appraised each article independently, and a third (CC), blinded to colleagues’ individual scores, resolved discrepancies. Since there are no standardised methods to assess equivalence, analysis technique, lay press and case reports publications, their risk of bias was not addressed.

### Patient and public involvement

Patients or the public were not involved in the design, or conduct, or reporting, or dissemination plans of our research.

## Results

### Types of publications and studies

A total of 10 137 publications were identified through database searches. After removal of 2300 duplicates, 7837 remaining publications were screened by title and abstract, and 475 articles were eligible (online supplemental file 4). Non-academic Google and medicine quality website searches yielded an additional 126 articles, and 68 publications with data on antibiotic quality previously identified in the MQRG scientific literature database were also included. Twenty-one studies were excluded due to being in languages not included in our review, or the full text not being available, resulting in a total of 648 included publications (online supplemental file 5). Four hundred and ninety-eight publications were original research publications from scientific journals, with 34 short communications, 33 public alerts, 32 lay press publications, 28 institutional reports, 17 theses, 3 articles discussing drug regulation/supply and 2 book/book chapters. The United States Pharmacopoeia Medicines Quality Database (https://www.usp.org/global-public-health/medicines-quality-database) was considered as a single ‘publication’.

10.1136/bmjgh-2022-008587.supp4 Supplementary data

10.1136/bmjgh-2022-008587.supp5 Supplementary data

Of the 498 original research articles published in scientific journals, 225 described analytical techniques, 89 were prevalence surveys, 101 were equivalence studies, 51 were reviews, 14 were stability studies and 11 postmarketing surveillance studies. Five were case reports of falsified antibiotics or their deleterious effects on patients, one article studied tetracycline bioavailability as a surrogate for its quality^40^ and one article studied how substandard antibiotics could affect AMR by using different concentrations of antibiotics.^41^

All data are mapped in the IDDO Quality Surveyor (https://www.iddo.org/mqsurveyor/%23antibiotics) and can be freely downloaded.

### Prevalence surveys

#### Characteristics of prevalence surveys

**Figure 1:**
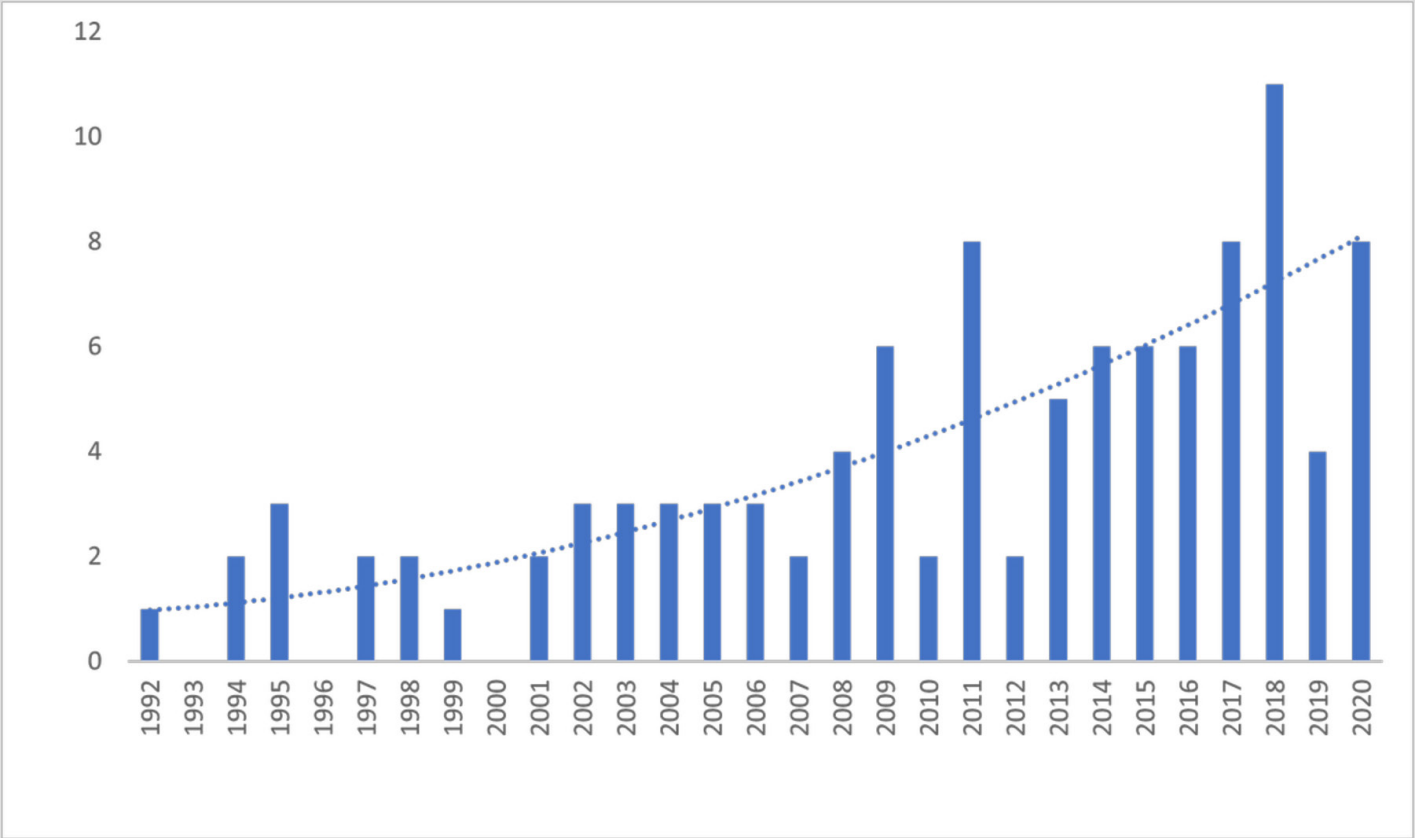
Number of prevalence surveys (y-axis) pertinent to antibiotic quality published per year (x-axis). A second-order polynomial trendline is represented as the blue dotted line.

Data from 106 prevalence surveys were included in the quantitative analysis as one paper with aggregated data was excluded from analysis.^42^ Eighty-nine (84.0%) papers were published as original research articles, eight (7.5%) were institutional reports, four (3.8%) short communications and five (4.7%) were theses. Of the 106 surveys, 76.4% (81/106) were published in peer-reviewed journals. Publication dates ranged between 1992 and 2020, but more than half (62.3%, 66/106) were published between 2010 and 2020 (figure 1). In total, 13 555 samples, originating from 67 different countries, were tested for quality in prevalence surveys (online supplemental file 6). Seventy (66.0%, 70/106) of the included studies used convenience sampling, 30 used random sampling (28.3%, 30/106), 1 study used a combination of both, 4 did not disclose the sampling method and 1 study piloted Lot Quality Assurance Sampling.^43^

10.1136/bmjgh-2022-008587.supp6 Supplementary data

#### Antibiotic failure frequency

**Figure 2:**
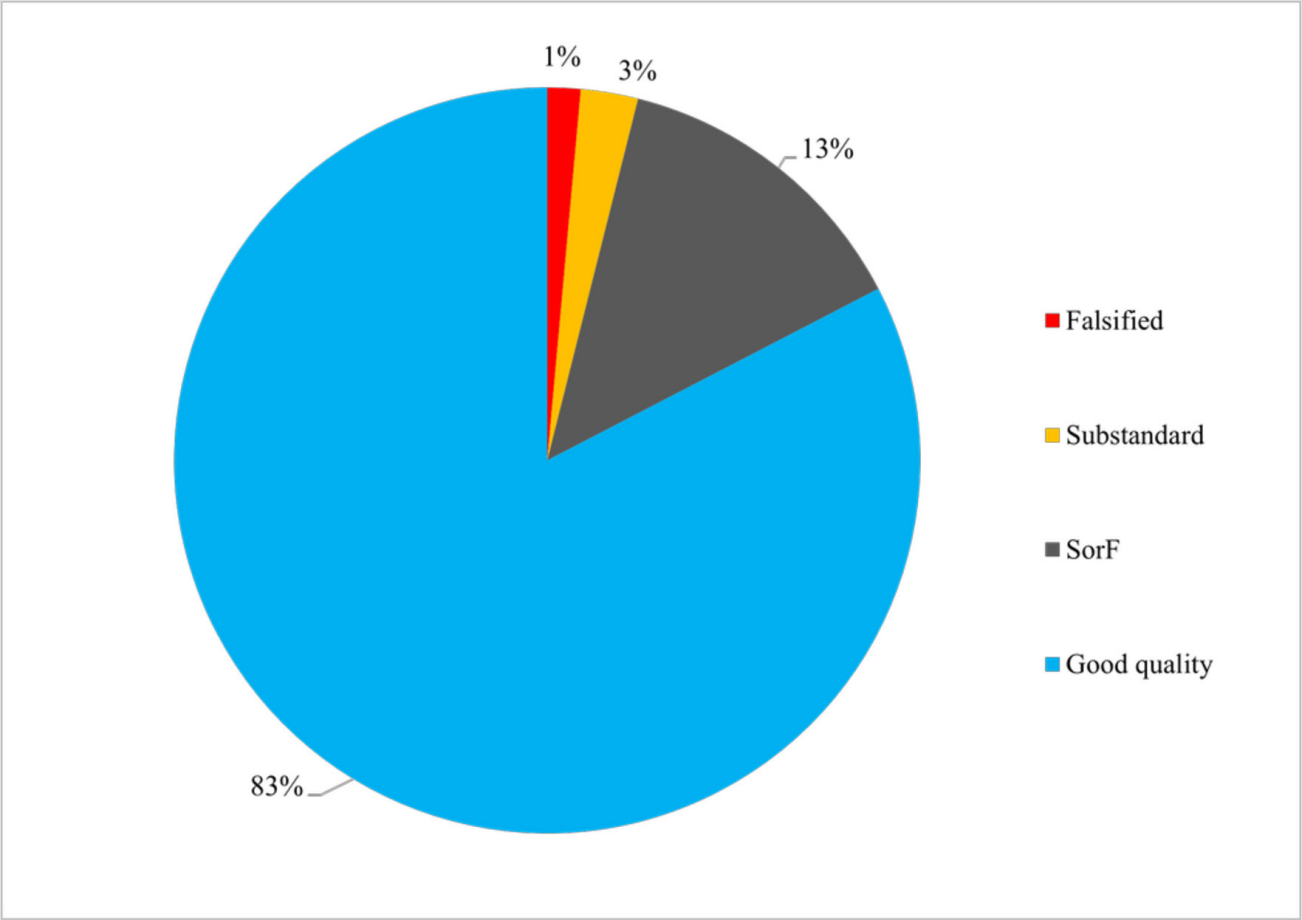
Quality categories of samples from antibiotic quality prevalence studies. Samples are classed as of ‘good quality’ if they passed all the tests performed by the investigators of a given study, which often do not cover the full pharmacopoeial specifications. Substandard and falsified samples are those who have failed at least one of the tests performed by the investigators. SorF, substandard or falsified.

Of the 13 555 samples included in the analysis, 2357 (17.4%) failed at least one quality test. Of those, 336 (14.3%) samples were substandard, 195 (8.3%) falsified and authenticity was not investigated for 1826 (77.5%) and are therefore considered SorF. We did not find any samples identified as ‘degraded’. The proportions of SorF, substandard and falsified medicines from prevalence surveys were thus 13.5%, 2.5% and 1.4%, respectively (figure 2). The median (Q1–Q3) number of samples collected per survey was 47 (21–135) and the median FF (at least one quality test failing) per survey was 19.6% (7.6%–35.0%). Thirteen (12%) surveys found no SF antibiotics (online supplemental file 6).

#### Failure frequency—geographical distribution

Of the 534 data points, 267 (50.0%) were from Africa, 185 (34.6%) from Asia, 31 (5.8%) from the Americas, 9 (1.7%) from Europe and 14 (2.6%) from Oceania (online supplemental file 7). There were 28 (5.2%) ‘unknown’ data point locations, with the FF not broken down by geography. Most samples were collected in sub-Saharan Africa (n=3825, 28.2%), South-East Asia (n=3555, 26.2%) and South Asia (n=1388, 10.2%). The highest observed antibiotic FF was in Africa (28.4%, 1090/3836), followed by Asia, Oceania, Americas and Europe at 15.2% (943/6210), 15.0% (15/100), 12.5% (112/898) and 7.7% (5/65), respectively.

10.1136/bmjgh-2022-008587.supp7 Supplementary data

**Figure 3:**
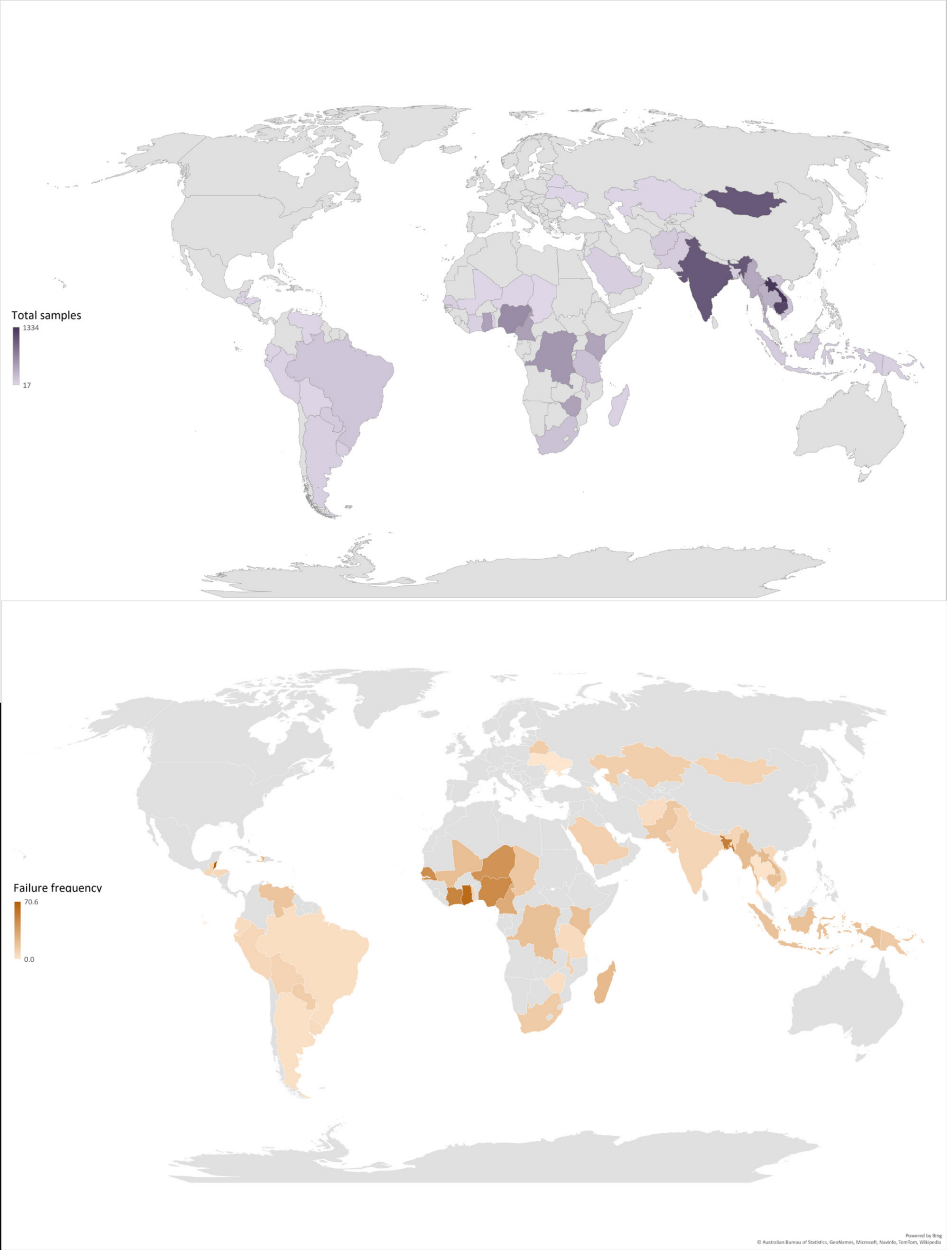
Global distribution of the evidence on antibiotics quality: total number of samples included in prevalence surveys (A) and failure frequency (B); countries with <15 samples have been greyed out. Caution must be exercised when drawing conclusions from these graphs. Samples from a given country may originate from a study sampling a single small urban or rural area, authorised or illicit outlets only (or a mix), etc and are not representative of medicine quality in the whole country. See online supplemental file 6 for further details.

The median (Q1–Q3) number of samples tested per country was 56 (15–180). The five countries with the most samples reported were the Lao People’s Democratic Republic (n=1334), Cambodia (n=1208), India (n=1055), Mongolia (n=1053) and Nigeria (n=632), with FF of 22.3%, 19.6%, 8.2%, 9.7% and 47.2%, respectively. Due to data aggregation, it was not possible to identify the country for 18.5% (2511/13 555) of samples. Sample numbers and FF per country are presented in figure 3, showing the disparity in the origin of the evidence, and potential ‘hotspots’ for SF antibiotics. Furthermore, 19.5% (2643/13 555) of the samples originated from low-income countries (LICs), 54.3% (7355/13 555) from LMICs, 3.6% (486/13 555) from upper-middle-income countries and 3.9% (524/13 555) from high-income countries (HICs). Due to data aggregation, it was not possible to identify the country-level income for 18.8% (2547/13 555) of samples.

#### Antibiotic failure frequency by AWaRe classification

Twenty-one (43.8%) APIs of the 48 included in the 2019 WHO AWaRe Access group and 16 (14.5%) of the 110 in the Watch group were investigated in prevalence surveys, with no Reserve group or carbapenems included.^23^ We found data for two APIs not included in the AWaRe classification (nalidixic acid, sulfamethoxazole). The antibiotics with the highest number of samples collected were ciprofloxacin (n=3511, 25.9%), amoxicillin (n=2208, 16.3%), sulfamethoxazole-trimethoprim (n=1255, 8.6%), tetracycline (n=1191, 9.3%) and ampicillin (n=1010, 5.5%). The overall FF for these APIs were 10.4% (366/3511), 16.1% (355/2208), 26.2% (329/1255), 12.1% (144/1191) and 20.9% (211/1010), respectively. Antibiotics in the Access group had an overall FF of 19.5% (1633/8354) and those in the Watch group 13.8% (718/5191) (online supplemental file 8).

10.1136/bmjgh-2022-008587.supp8 Supplementary data

#### Techniques used and quality defects of samples

More than one quality test was conducted in 67.9% (72/106) of the prevalence surveys and the median (range) number of analysis techniques per survey was 3 (1–5). API content analysis was performed in 79.2% (84/106) of prevalence surveys, with 76.0% (10 307/13 555) of the samples tested and an FF of 16.5% (1701/10 307) (table 1). Of the samples tested for API content against the amount stated on the label and pharmacopoeial limits, 6.4% (662/10 307) were found to contain lower API, 1.3% (131/10 307) contained no API and 2.4% (249/10 307) contained higher API content. For 6.4% (659/10 307) of samples some dosage units contained lower API, and some contained higher API than stated, or samples were reported as having the incorrect API amount without further details given.

**Table 1:** Percentage of failing samples per type of quality analysis in the prevalence studies

Quality component	FF % (n/N)
API content	16.5 (1701/10 307)
Dissolution	9.1 (296/3261)
API ID and semi-quantitation	7.5 (210/2783)
Impurity/Contaminant/Related substance	3.5 (12/346)
Packaging/Label/Physical appearance inspection	2.8 (129/4612)
Other chemical tests*	4.4 (187/4212)
Other physical tests†	2.2 (71/3290)

One sample may have been tested for more than one quality test.

*API identification, degradation products, pH and other undeclared chemical tests.

†Includes disintegration, friability, hardness, thickness, wetting time and water absorption testing.

API, active pharmaceutical ingredient; FF, failure frequency.

Dissolution testing was performed in 34/106 (32.1%) of surveys; 9.1% samples (296/3261) failed, making this the second most common defect found. Packaging analysis/visual inspection of dosage units were the third most frequently performed analyses, included in 31/106 (29.2%) of surveys, with a failure of 2.8% (129/4612) of those tested. Some APIs showed signals of concern for specific tests. Of all those that failed, flucloxacillin had an FF for API content of 85.7% (18/21), azithromycin of 65.6% (21/32), erythromycin of 48.3% (28/58), ampicillin-cloxacillin of 41.7% (80/192) and chloramphenicol 31.7% (40/126). Of samples that failed, dissolution failures were 69.2% (9/13) for erythromycin, 29.7% (19/64) for levofloxacin, 29.8% (17/57) for roxithromycin and 28.4% (25/88) for clarithromycin (online supplemental file 9).

10.1136/bmjgh-2022-008587.supp9 Supplementary data

#### Failure frequency by source

Outlets where antibiotics had been collected were reported for 48.9% (6624/13 555) of samples in prevalence surveys (table 2). In total, 6616/13 555 (48.8%) samples from 33 countries (255 different data points) were collected in outlet combinations, without breakdown by outlet type. The overall FF for such aggregated samples was 18.3% (1211/6616). Four studies did not contain explicit information on the outlets where samples were collected.^44–47^

**Table 2:** Failure frequency of antibiotics by outlet type in prevalence surveys

Outlet/Source	Failure frequency % (n/N)	Data points	Countries
Combination of outlets*	18.3% (1211/6616)	255	Afghanistan, Armenia, Azerbaijan, Belarus, Belize, Cambodia, Cameroon, Chad, Democratic Republic of the Congo, Estonia, Ghana, India, Indonesia, Kazakhstan, Kenya, Lao People’s Democratic Republic, Madagascar, Malawi, Mongolia, Myanmar, Niger, Nigeria, Papua New Guinea, Russian Federation, Rwanda, Senegal, Sudan, Tanzania, Thailand, UK, Uzbekistan, Viet Nam, Zimbabwe
Government clinics/depots	22.1% (44/199)	8	Cambodia, Cameroon, Myanmar, South Africa
Hospitals/Health centres	4.8% (26/543)	16	Cameroon, India, Kazakhstan, Kenya, Tanzania, Ukraine, Zimbabwe
Internet	4.3% (11/255)	5	India, USA
Private pharmacies	15.7% (707/4510)	120	Argentina, Bangladesh, Bolivia, Brazil, Cambodia, Cameroon, China, Ecuador, Ethiopia, Ghana, Guatemala, Honduras, India, Kenya, Lao People’s Democratic Republic, Malawi, Mexico, Nigeria, Pakistan, Papua New Guinea, Paraguay, Peru, Saudi Arabia, Sierra Leone, South Africa, Tanzania, Thailand, Togo, USA, Uruguay, Venezuela
Unknown†	16.2% (51/315)	12	Bangladesh, Cambodia, Cameroon, Ethiopia, Lao People’s Democratic Republic, Thailand
Unregistered/Unlicensed outlets‡	34.3% (210/613)	36	Cameroon, Côte d'Ivoire, India, Kenya, Nigeria, Pakistan, Senegal, Thailand
Wholesalers/Distributors	19.3% (97/504)	82	Burkina Faso, Democratic Republic of the Congo, Germany, Kazakhstan, Kenya, Madagascar, Mali, Nepal, Nigeria, South Africa, Tajikistan, Tanzania, Uganda, Viet Nam, Zimbabwe

*Nearly half of the surveys described several types of outlets where medicines were collected in the methods but did not present their results broken down by individual types of outlets.

†Four studies did not explicitly mention the outlets where samples were sourced.

‡Includes unlicensed/unregistered market stalls, shops, ambulant sellers, etc.

When authors reported the results by outlet type, private pharmacies were the most commonly sampled, representing 33.3% (4510/13 555) of the samples collected in 31 different countries (120 data points), with an FF of 15.7% (707/4510). The type of outlet with the highest FF was unregistered/unlicensed, with samples collected in eight countries, and an FF of 34.3% (210/613). Samples from hospitals/health centres were collected in 7 countries with an FF of 4.8% (26/543), wholesalers/distributors from 15 countries, with FF of 19.3% (97/504) and internet pharmacies with data from 2 countries and an FF of 4.3% (11/255). The FF in government facilities was 22.1% (44/199), with samples from four countries.

#### Reporting bias assessment

Eighty-two prevalence surveys met the inclusion criteria for appraisal using MEDQUARG. Fifty-four (65.9%) were published after MEDQUARG publication in 2009; eight (14.8%) of those stated that they used this. Over the 82 surveys, the number (%) of MEDQUARG items reported ranged from 2/26 (7.7%) to 23/26 (88.5%), with a mean score of 11 (95% CI 10.1 to 12.2) and a mean proportion of agreement of 42.3% (online supplemental file 9). Scores were significantly higher for surveys published after 2009 with a mean difference of concordance of 4.5 (95% CI 2.4 to 6.5, t-test, p<0.001) (online supplemental file 10).

10.1136/bmjgh-2022-008587.supp10 Supplementary data

**Figure 4:**
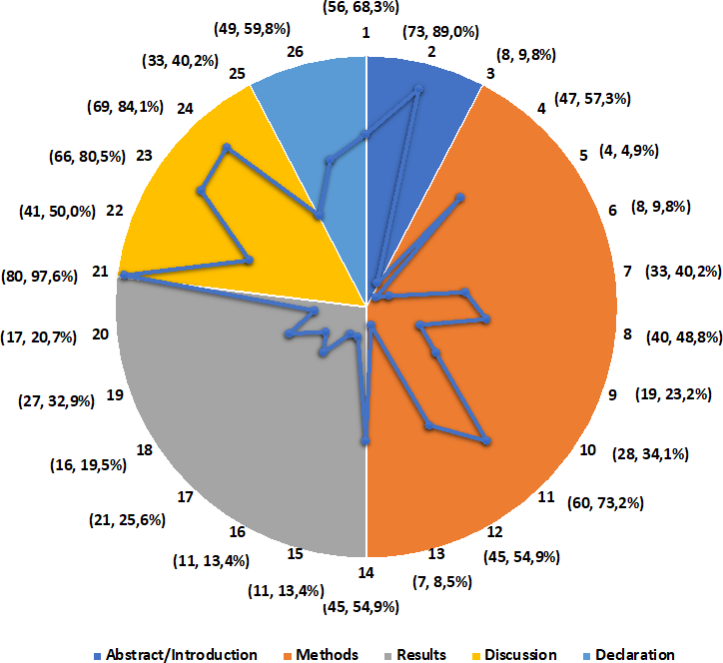
Frequency and proportion of prevalence surveys (out of 82) by individual Medicine Quality Assessment Reporting Guidelines checklist items reported.

Fifty-six prevalence surveys out of 82 (68.3%) were identified as such in their titles, and their abstract included sufficient details of methods (figure 4). Quality of medicines definitions were provided in 47 (57.3%) studies. Eight studies (9.8%) specified the time frame for samples collection and analysis, eight (9.8%) provided information on how outlets were selected and how sample size was determined. Thirty-three (40.2%) studies reported whether sampling was conducted by covert shoppers or not and the reason the shopper gave to the seller for the purchase. Twenty-one (25.6%) studies clearly categorised the samples as genuine, falsified, substandard, other equivalent terminology or provided reasons for not having done so. The MRA of the country where samples were collected was either involved in conducting the survey or was stated as informed of the results in 27 (32.9%) studies.

### Equivalence studies

One hundred and five publications assessed the quality of different antibiotic brands containing the same API(s) (online supplemental file 11). A total of 1090 samples, with a median of 9 (95% CI 5 to 12) samples per study, were collected in 51 countries (174 data points). These included 32 API/API combinations, 12 belonging to Access and 20 to Watch classes. The API/API combinations with the greatest number of studies were ciprofloxacin (n=32), metronidazole (n=17) and amoxicillin or amoxicillin-clavulanic acid (n=16). The overall FF was 25.9% (282/1090) and the median FF per study was 4.5% (95% CI 0.0% to 40.0%). The median number of techniques used to test the quality of each sample was 5 (95% CI 2 to 6). The tests most commonly failed were the API identification and semi-quantitation (15.2% (34/223)), API content (12.4% (155/1251)), dissolution (9.6% (59/616)) and impurities/contaminants/related substances (8.2% (8/98)).

10.1136/bmjgh-2022-008587.supp11 Supplementary data

### Seizures, recalls and case reports

Sixty-six publications described recall/warning/alerts (n=41), seizures (n=18) and case reports (n=7) of antibiotics with quality issues, published between 1991 and 2020, in 24 countries (online supplemental file 12). In total, 120 API/API combinations were listed, 71 belonging to Access, 48 to Watch classes; one was not included in the AWaRe classification, and four articles did not state the API. Articles mostly described instances of SorF (n=41) and falsified antibiotics (n=27); 14 articles described substandard products, 4 of products unregistered in the country they were marketed in and 5 with unspecified details. Out-of-specifications API content and identification test failures were most commonly described (n=39); 11 contained no API, 5 contained lower API than stated, 4 contained wrong API(s) and in 19 cases no such details were given.

10.1136/bmjgh-2022-008587.supp12 Supplementary data

## Discussion

The quantitative analysis of 106 prevalence surveys from 67 different countries and 13 555 samples tested for quality, resulted in an overall FF of 17.4%. Most samples were from LICs and LMICs in Africa and Asia. The majority were antibiotics from the Access group, a small proportion from the Watch group and none from the Reserve group. Sulfamethoxazole-trimethoprim had the highest FF, followed by ampicillin, amoxicillin, ciprofloxacin and tetracycline. Limited tests were used to assess the quality of samples, with a focus on API content and, to a lesser extent, dissolution and packaging analysis. The survey methodology and reporting were of low quality, many surveys were of small sample size, and samples often collected using convenience sampling. These data cannot and should not be interpreted that 17.4% of the global supply of antibiotics are SF but they do point to severe focal issues necessitating interventions to improve the global antibiotic supply.

Similar to results from previous reviews,^6 48 49^ most samples were collected in Asia and Africa, with many fewer samples from elsewhere. The highest FFs were found in Africa, followed by Asia. A review from 14 years ago also found the proportion of reported SF antibiotics to be highest in these regions but was lower in Africa than Asia at 18.7% and 33.6%, respectively.^50^ This shift, also described in a recent review,^51^ could be caused by the increasing research output from Africa, expansion of pharmaceutical supply, increased access and demand and an early stage regulatory environment in comparison with many Asian countries.

Quality assessment surveys have focused on the AWaRe Access class, representing more than three-fifths of samples included in prevalence surveys identified here. The remainder were Watch class antibiotics, with more than two-thirds of these samples being ciprofloxacin. Of concern, no data for Reserve class antibiotics were found, which may in part result from these antibiotics being relatively rarely used in Africa and Asia.^52^ No surveys on vancomycin or antipseudomonal penicillins’ quality were identified.

API content defects were the most frequently encountered. The most striking examples were from a survey in Bangladesh, which reported cephadrine and ciprofloxacin samples containing 1% and 1.5% of the stated amounts, respectively.^38^ A study in the Western Pacific Islands identified cloxacillin tablets containing 6.9% of the stated amount.^39^ In Kenya, parenteral ampicillin were found to contain 190% of the stated amount,^40^ and in India azithromycin tablets contained 160% of the stated amount.^41^ Lower API amount than stated on the label was found for >6% of all samples tested for API content. Dissolution failure was also observed in almost one-tenth of samples tested. These findings show that SF antibiotics may expose patients to subtherapeutic levels and risk the selection and spread of resistant bacteria.^14^ There is a lack of evidence to what degree quality defects will translate in impaired antibiotic bioavailability. Would an 85% API content antibiotic, when the pharmacopoeial lower limit is set to 90%, have clinically significant effects on patient outcome and AMR for different bacterial pathogens? Pharmacodynamic-pharmacokinetic modelling to understand the relationship between quality defect(s), such as low API content or impaired dissolution, to clinical outcomes are needed.

Aggregate results of prevalence surveys were reported for combinations of outlet types for more than half of samples, similar to the WHO findings, making it difficult to assess the sources of SF antibiotics.^9^ When outlet types were specified, private registered pharmacies were the most reported, with a 15.7% FF. The highest FF of 34.3% was for samples from unregistered and unlicensed outlets, but data points were few.

Combining AMC data with the prevalence of SF antibiotics could be a novel, although crude, method to assess population risk of consumption of SF antibiotics. This could allow policy makers to focus their efforts on agents with high AMC in relation to their quality issues and provide insights into SF antibiotics-AMR relationships. We paired global AMC data with the SF antibiotic data summarised here, multiplying median AMC and FF.^52 53^ However, AMC reporting countries were mostly MIC or HIC. Beta-lactams and penicillins had the highest index (online supplemental file 13). Future SF prevalence surveys also collecting sympatric AMC or antimicrobial use (AMU) data could be a useful approach for estimating risk of SF antibiotics in supply chains and hence risk of SF antibiotics on both patient outcomes and AMR. Improved AMC and AMU reporting (eg, in Laos https://www.youtube.com/watch?v=QELwHIPsKw4) is needed in order to rank the local importance, or otherwise, of SF antibiotics as drivers of AMR and allow targeted policymaking.

10.1136/bmjgh-2022-008587.supp13 Supplementary data

The overall FF in prevalence surveys observed here is higher than estimates in other recent reviews such as that by Ozawa *et al*, who focused on 11 studies and reported an FF of 12.4%.^49^ Comparison with the WHO 2017 review^10^ is difficult because the antimicrobials category also included ‘other anti-infective products’ (FF of 7.2%). Great caution is needed when interpreting the findings, as the higher FF in our review may be influenced by the inclusion of studies with smaller sample sizes. Half of the surveys included <47 samples; these had a median FF of 22.8%.

Results are strongly influenced by the heterogeneity, and the low methodological quality of studies included, as mirrored by the low MEDQUARG scores. Gaps in the quality of the methods and results reporting were evident, such as poor sampling design or lack of packaging analysis. Two-thirds of all studies used convenience sampling, risking bias and misestimation of SF antibiotic prevalence.^10 16 49 54^ We are further limited by the inclusion of studies published only in English, French and Spanish and by medicine regulatory authority and the pharmaceutical industry datasets mostly not being in the public domain.^10 16 38 49 54^

Most studies performed less than five tests to assess adherence to the different pharmacopoeial properties, budgetary constraints being a common hurdle encountered.^55–57^ Difficulties in collecting sufficient dosage units required, especially in remote areas and when sampling using a ‘mystery shopper’ approach are also barriers. This has important implications since samples are classed as of good quality if they pass the reduced battery tests conducted instead of full pharmacopoeial standards. Therefore, an antibiotic could be reported as being of good quality due to having a within-range amount of API but may actually be SF due to other key aspects not being tested for. Such underestimation of the prevalence of SF antibiotics is supported by the higher FF found in bioequivalence studies in which the median number of quality tests performed per sample was higher than in prevalence surveys.

Accurate data on the epidemiology of SF antibiotics are key to understanding the problem and planning and prioritising interventions. The true prevalence of SF medicines can only be known from good quality evidence. The MEDQUARG and subsequent WHO guidelines and checklists provide a framework for better quality field surveys and reporting of medicine quality studies.^39 58^ Key features include randomised sampling of medicines with appropriate sample size; ‘mystery shopper’ sampling to mimic real-life procurement of medicines; minimised timing between collection and analysis and appropriate storage in-between collection and analysis; following pharmacopoeia monographs to test the quality of medicines, or at a minimum API identity and content compared with recommended, and dissolution rate. Packaging analysis is also important to differentiate between SF medicines but this is often difficult as genuine packaging should be obtained from pharmaceutical companies, some being hard to reach and committing to the requirement. Calculation of the required sample size is also vital. Due to the costs associated with pharmacopoeial analysis and randomised sampling, and in light of the severe implications SF antibiotics carry, more funding is needed to support such studies and allow access to a wider range of chemical analysis methods.

These data show reasons for global concern as SF antibiotics risk increased morbidity, mortality, patient health expense, decline in confidence in health systems, economic harm to societies, governments and the pharmaceutical industry and AMR. SF antibiotics in the Watch and Reserve classes could severely impact patient outcomes in sub-Saharan Africa, Oceania and Asia, where sepsis incidence and sepsis-related mortality are highest.^59^ Poor antibiotic quality appears to affect all antibiotic classes, especially those which are most consumed and in countries facing greater socioeconomic challenges.^52^ Furthermore, unlicensed outlets seem to be particularly affected by this public health threat. SF antibiotics are likely to impede achieving the Sustainable Development Goals and controlling AMR in pathogens of key global importance such as those included in the WHO Global Antimicrobial Resistance Surveillance System initiative.^56 60^ Lastly, with increasing access to antibiotics and higher AMC, failure to tackle quality problems will inevitably lead to higher absolute numbers of SF antibiotics reaching the population, causing direct harm and engendering further AMR.

The relationship between SF antibiotics and AMR is tangled as SF antibiotics are likely to be found where poor adherence and prescribing are sympatric, and there is little understanding of the delay between bacterial communities being exposed to subtherapeutic antibiotic concentrations and rising AMR prevalence being observed. In a recent study, the abundance and diversity of resistance genes was mainly correlated to sanitation and population health at local and national levels, without including an assessment of the impact of antibiotic quality.^61^ The highest predicted diversity of AMR genes was found in Africa, which was also the region with the highest concentration of antibiotics in urban sewage, and where the highest FF was observed here.^14 61^ Whether SF AMC contributes disproportionately, in comparison to good quality AMC, to AMR gene diversity at an individual level, which then spreads horizontally in regions with poor sanitation, leading to worsened health outcomes which then affect AMC in a vicious cycle, needs exploring.

In addition to global efforts to promote responsible use of antibiotics in humans and in animal feed, interventions as recommended in the WHO’s SF ‘Prevent, Detect, Respond’ strategy and enhanced regulatory oversight of antibiotic manufacture are needed without waiting for better evidence.^49 62^ A comprehensive effort to alleviate this public health issue must include policy interventions to ensure access to good quality antibiotics and control of AMR.

## Conclusion

Antibiotic quality epidemiology remains a poorly understood and neglected topic, as shown in recent analyses of the AMR situation that did not include medicine quality as a potential driver.^63^ Logically SF antibiotics will increase the risk of adverse patient outcomes, cause economic harms and drive AMR. These data argue that nations and international organisations should assess and prioritise interventions to enhance regulatory, purchasing and financial mechanisms to improve the global antibiotic supply.^64^ How interventions to reduce SF antibiotics should be ranked as priorities in national AMR action plans is unclear. WHO Prevent, Detect and Respond strategies are key,^62^ and portable, affordable screening devices offer hope for empowering drug inspectors for postmarket surveillance of antibiotic quality.^65^

## Data Availability

All data relevant to the study are included in the article or uploaded as supplementary information.
