# Supplementary material for: Substandard and falsified antibiotics: neglected drivers of antimicrobial resistance?
Source: BMJ Glob Health. 2022 Aug 18;7(8):e008587. doi: 10.1136/bmjgh-2022-008587 (PMC9394205; doi:10.1136/bmjgh-2022-008587)
Supplement: Supplementary data [file bmjgh-2022-008587supp001.pdf]

Substandard and falsified antibiotics: neglected drivers of antimicrobial resistance?

Supplementary file 1. Search terms.

PUBMED

(substandard OR falsified OR counterfeit OR fake OR spurious OR degraded OR “falsely labelled” OR “drug* qualit*” OR “medicine* qualit*” OR “pharmaceutical* qualit*” OR “poor qualit*”) AND (antibacterial* OR antibiotic* OR antimicrobial* OR anti-infective* OR demeclocycline OR doxycycline OR chlortetracycline OR lymecycline OR metacycline OR oxytetracycline OR tetracycline* OR minocycline OR rolitetracycline OR penimepicycline OR clomocycline OR omadacycline OR tigecycline OR eravacycline OR sarecycline OR chloramphenicol OR thiamphenicol OR ampicillin OR pivampicillin OR carbenicillin OR amoxicillin OR carindacillin OR bacampicillin OR epicillin OR pivmecillinam OR azlocillin OR mezlocillin OR mecillinam OR piperacillin OR ticarcillin OR metampicillin OR talampicillin OR sulbenicillin OR temocillin OR hetacillin OR aspoxicillin OR benzylpenicillin OR phenoxymethylpenicillin OR propicillin OR azidocillin OR pheneticillin OR penamecillin OR clometocillin OR “benzathine benzylpenicillin” OR “procain* benzylpenicillin” OR “benzathine phenoxymethylpenicillin” OR dicloxacillin OR cloxacillin OR methicillin OR oxacillin OR flucloxacillin OR nafcillin OR sulbactam OR tazobactam OR avibactam OR sultamicillin OR cefalexin OR cefaloridine OR cefalotin OR cefazolin OR cefadroxil OR cefazedone OR cefatrizine OR cefapirin OR cefradine OR cefacetrile OR cefroxadine OR ceftezole OR cefoxitin OR cefuroxime OR cefamandole OR cefaclor OR cefotetan OR cefonicid OR cefotiam OR loracarbef OR cefmetazole OR cefprozil OR ceforanide OR cefminox OR cefbuperazone OR flomoxef OR cefotaxime OR ceftazidime OR cefsulodin OR ceftriaxone OR cefmenoxime OR latamoxef OR ceftizoxime OR cefixime OR cefodizime OR cefetamet OR cefpiramide OR cefoperazone OR cefpodoxime OR ceftibuten OR cefdinir OR cefditoren OR cefcapene OR cefteram OR cefepime OR cefpirome OR cefozopran OR ceftolozane OR aztreonam OR carumonam OR meropenem OR ertapenem OR doripenem OR biapenem OR tebipenem OR imipenem OR panipenem OR “ceftobiprole medocaril” OR “ceftaroline fosamil” OR “faropenem” OR trimethoprim OR brodimoprim OR iclaprim OR sulfaisodimidine OR sulfamethizole OR sulfadimidine OR sulfapyridine OR sulfafurazole OR sulfanilamide OR sulfathiazole OR sulfathiourea OR sulfamethoxazole OR sulfamoxole OR sulfadiazine OR sulfadimethoxine OR sulfalene OR sulfametomidine OR sulfametoxydiazine OR sulfamethoxypyridazine OR sulfaperin OR sulfamerazine OR sulfaphenazole OR

Zabala GA, *et al. BMJ Global Health* 2022; 7:e008587. doi: 10.1136/bmjgh-2022-008587

sulfamazone OR erythromycin OR spiramycin OR midecamycin OR oleandomycin OR roxithromycin OR josamycin OR troleandomycin OR clarithromycin OR azithromycin OR miocamycin OR rokitamycin OR dirithromycin OR flurithromycin OR telithromycin OR solithromycin OR clindamycin OR lincomycin OR pristinamycin OR quinupristin OR dalfopristin OR streptomycin OR streptoduocin OR tobramycin OR gentamicin OR kanamycin OR neomycin OR amikacin OR netilmicin OR sisomicin OR dibekacin OR ribostamycin OR isepamicin OR arbekacin OR bekanamycin OR plazomicin OR ofloxacin OR ciprofloxacin OR pefloxacin OR enoxacin OR temafloxacin OR norfloxacin OR lomefloxacin OR fleroxacin OR sparfloxacin OR rufloxacin OR grepafloxacin OR levofloxacin OR trovafloxacin OR moxifloxacin OR gemifloxacin OR gatifloxacin OR prulifloxacin OR pazufloxacin OR garenoxacin OR sitafloxacin OR tosufloxacin OR delafloxacin OR rosoxacin OR nalidixic acid OR piromidic acid OR pipemidic acid OR oxolinic acid OR cinoxacin OR flumequine OR nemonoxacin OR vancomycin OR teicoplanin OR telavancin OR dalbavancin OR oritavancin OR colistin OR “polymyxin B” OR “fusidic acid” OR metronidazole OR tinidazole OR ornidazole OR azanidazole OR propenidazole OR nimorazole OR secnidazole OR nitrofurantoin OR nifurtoinol OR furazidin OR fosfomycin OR xibornol OR clofoctol OR spectinomycin OR methenamine OR “mandelic acid” OR nitroxoline OR linezolid OR daptomycin OR bacitracin OR tedizolid OR lefamulin OR macrolide* OR tetracycline* OR Beta-Lactam* OR Sulfonamide* OR lincosamide* OR aminoglycoside* OR *quinolone* OR glucopeptide* OR lipopeptide OR cephalosporin* OR “penicillin V” OR penicillin OR “penicillin G” OR co-amoxiclav OR Co-fluampicil OR Co-trimoxazole OR cephalexin OR cephaloridine OR cephalothin OR cephazolin OR cephapirin OR cephradine OR cephacetrile)

EMBASE

(substandard OR falsified OR counterfeit OR fake OR spurious OR degraded OR “falsely labelled” OR “drug quality” OR “medicine quality” OR “pharmaceutical quality” OR “poor quality”) AND (antibacterial OR antibiotic OR antimicrobial OR anti-infective OR demeclocycline OR doxycycline OR chlortetracycline OR lymecycline OR metacycline OR oxytetracycline OR tetracycline OR minocycline OR rolitetracycline OR penimepicycline OR clomocycline OR omadacycline OR tigecycline OR eravacycline OR sarecycline OR chloramphenicol OR thiamphenicol OR ampicillin OR pivampicillin OR carbenicillin OR amoxicillin OR carindacillin OR bacampicillin OR epicillin OR pivmecillinam OR azlocillin OR mezlocillin OR mecillinam OR piperacillin OR ticarcillin OR metampicillin OR talampicillin OR sulbenicillin OR temocillin OR hetacillin OR aspoxicillin OR benzylpenicillin OR phenoxymethylpenicillin OR propicillin OR azidocillin OR pheneticillin OR penamecillin OR

clometocillin OR “benzathine benzylpenicillin” OR “procain benzylpenicillin” OR benzathine phenoxymethylpenicillin OR dicloxacillin OR cloxacillin OR methicillin OR oxacillin OR flucloxacillin OR nafcillin OR sulbactam OR tazobactam OR avibactam OR sultamicillin OR cefalexin OR cefaloridine OR cefalotin OR cefazolin OR cefadroxil OR cefazedone OR cefatrizine OR cefapirin OR cefradine OR cefacetrile OR cefroxadine OR ceftezole OR cefoxitin OR cefuroxime OR cefamandole OR cefaclor OR cefotetan OR cefonicid OR cefotiam OR loracarbef OR cefmetazole OR cefprozil OR ceforanide OR cefminox OR cefbuperazone OR flomoxef OR cefotaxime OR ceftazidime OR cefsulodin OR ceftriaxone OR cefmenoxime OR latamoxef OR ceftizoxime OR cefixime OR cefodizime OR cefetamet OR cefpiramide OR cefoperazone OR cefpodoxime OR ceftibuten OR cefdinir OR cefditoren OR cefcapene OR cefteram OR cefepime OR cefpirome OR cefozopran OR ceftolozane OR aztreonam OR carumonam OR meropenem OR ertapenem OR doripenem OR biapenem OR tebipenem OR imipenem OR panipenem OR “ceftobiprole medocaril” OR “ceftaroline fosamil” OR faropenem OR trimethoprim OR brodimoprim OR iclaprim OR sulfaisodimidine OR sulfamethizole OR sulfadimidine OR sulfapyridine OR sulfafurazole OR sulfanilamide OR sulfathiazole OR sulfathiourea OR sulfamethoxazole OR sulfamoxole OR sulfadiazine OR sulfametrole OR sulfadimethoxine OR sulfalene OR sulfametomidine OR sulfametoxydiazine OR sulfamethoxypyridazine OR sulfaperin OR sulfamerazine OR sulfaphenazole OR sulfamazone OR erythromycin OR spiramycin OR midecamycin OR oleandomycin OR roxithromycin OR josamycin OR troleandomycin OR clarithromycin OR azithromycin OR miocamycin OR rokitamycin OR dirithromycin OR flurithromycin OR telithromycin OR solithromycin OR clindamycin OR lincomycin OR pristinamycin OR quinupristin OR dalfopristin OR streptomycin OR streptoduocin OR tobramycin OR gentamicin OR kanamycin OR neomycin OR amikacin OR netilmicin OR sisomicin OR dibekacin OR ribostamycin OR isepamicin OR arbekacin OR bekanamycin OR plazomicin OR ofloxacin OR ciprofloxacin OR pefloxacin OR enoxacin OR temafloxacin OR norfloxacin OR lomefloxacin OR fleroxacin OR sparfloxacin OR rufloxacin OR grepafloxacin OR levofloxacin OR trovafloxacin OR moxifloxacin OR gemifloxacin OR gatifloxacin OR prulifloxacin OR pazufloxacin OR garenoxacin OR sitafloxacin OR tosufloxacin OR delafloxacin OR rosoxacin OR nalidixic acid OR piromidic acid OR pipemidic acid OR oxolinic acid OR cinoxacin OR flumequine OR nemonoxacin OR vancomycin OR teicoplanin OR telavancin OR dalbavancin OR oritavancin OR colistin OR polymyxin B OR fusidic acid OR metronidazole OR tinidazole OR ornidazole ornidazole OR azanidazole OR propenidazole OR nimorazole OR secnidazole OR nitrofurantoin OR nifurtoinol OR furazidin OR fosfomycin OR xibornol OR clofoctol OR spectinomycin OR methenamine OR “mandelic acid” OR nitroxoline OR linezolid OR daptomycin OR bacitracin OR tedizolid OR lefamulin OR macrolide OR tetracycline OR Beta-Lactam OR Sulfonamide OR lincosamide OR aminoglycoside OR fluorquinolone OR quinolone OR glucopeptide OR lipopeptide OR cephalosporin OR penicillin V OR penicillin OR

penicillin G OR co-amoxiclav OR Co-fluampicil OR Co-trimoxazole OR cephalexin OR cephaloridine OR cephalothin OR cephazolin OR cephapirin OR cephradine OR cephacetrile)

Google and Google Scholar (sorted by relevance, selected first 200 for each search)

English language searches

1. (substandard OR falsified OR counterfeit OR fake OR spurious OR “drug quality” OR “medicine quality” OR “pharmaceutical quality”) AND (antibacterial OR antibiotic OR antimicrobial OR aminoglycoside OR Quinolone OR macrolide OR Beta-Lactam)
2. (substandard OR falsified OR counterfeit OR fake OR spurious OR “drug quality” OR “medicine quality” OR “pharmaceutical quality”) AND (Tetracyclines OR penicillin OR Cephalosporin)

French language searches

3. (sous-standard OR falsifié OR contrefaçon OR faux OR "médicament fallacieux" OR "qualité des médicaments" OR “qualité pharmaceutique”) AND (antibactérien OR antibiotique OR antimicrobien OR aminoside OR quinolone OR macrolide OR béta-lactamine)
4. (sous-standard OR falsifié OR contrefaçon OR faux OR "médicament fallacieux" OR "qualité des médicaments" OR “qualité pharmaceutique”) AND (tétracycline OR pénicilline OR céphalosporine)

Zabala GA, *et al. BMJ Global Health* 2022; 7:e008587. doi: 10.1136/bmjgh-2022-008587
