# Supplementary material for: Substandard and falsified antibiotics: neglected drivers of antimicrobial resistance?
Source: BMJ Glob Health. 2022 Aug 18;7(8):e008587. doi: 10.1136/bmjgh-2022-008587 (PMC9394205; doi:10.1136/bmjgh-2022-008587)

Substandard and falsified antibiotics: neglected drivers of antimicrobial resistance?

Supplementary file 4. PRISMA flow diagram of the selection process of the publications on antibiotic quality.

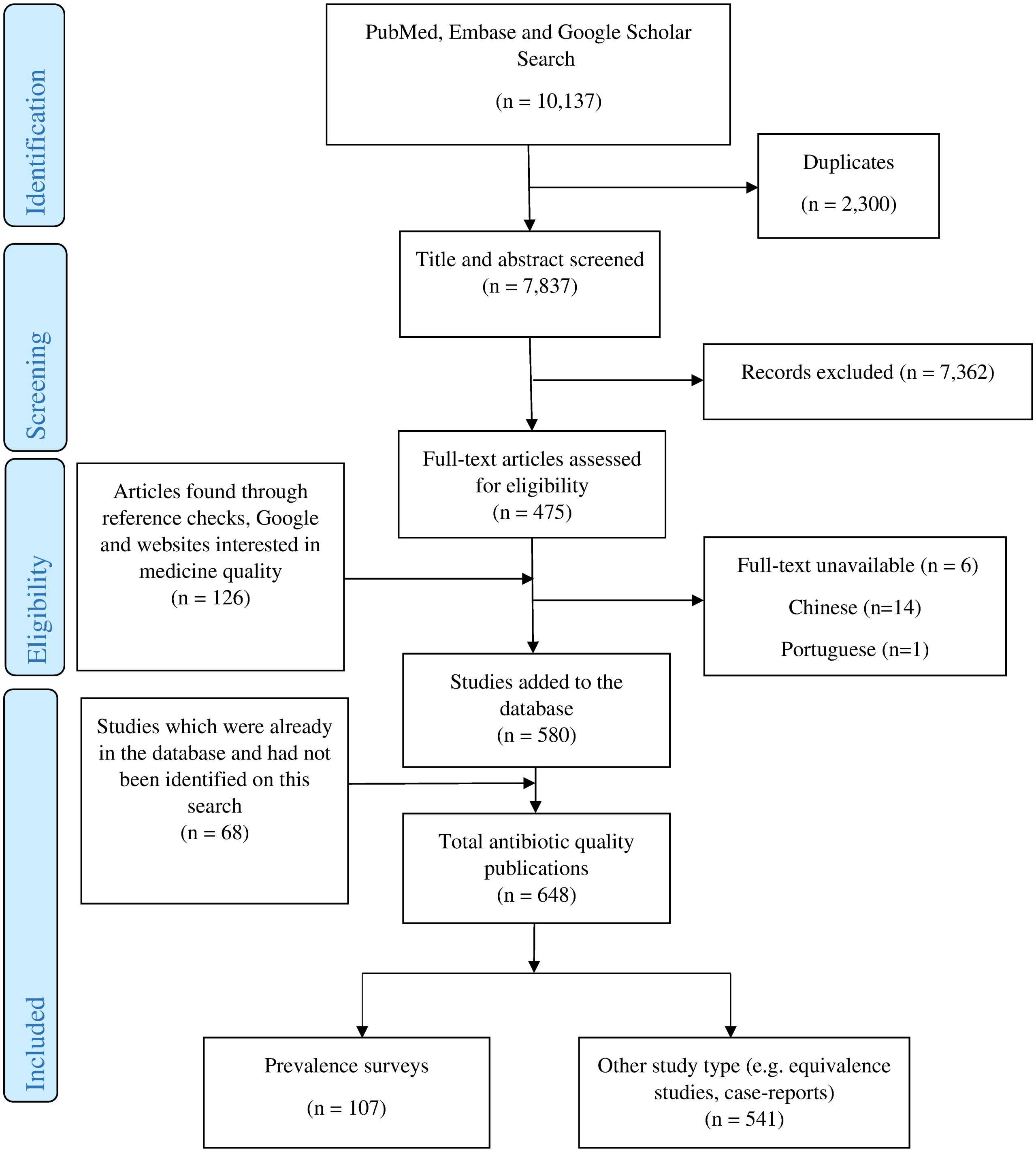

Supplement: Supplementary data [file bmjgh-2022-008587supp004.pdf]
