# Supplementary material for: Substandard and falsified antibiotics: neglected drivers of antimicrobial resistance?
Source: BMJ Glob Health. 2022 Aug 18;7(8):e008587. doi: 10.1136/bmjgh-2022-008587 (PMC9394205; doi:10.1136/bmjgh-2022-008587)
Supplement: Supplementary data [file bmjgh-2022-008587supp005.pdf]

Substandard and falsified antibiotics: neglected drivers of antimicrobial resistance?

Supplementary file 5. Papers excluded post eligibility assessment of full text or unable to obtain full text

Unable to obtain full text

1. Agom JK, Akanni AO, Dawodu TO. Quality of ampicillin/cloxacillin preparations on the Nigerian market. Nig J Pharmacol 1990; 21: 36-38.
2. Hailu GS, Gutema GB, Hishe HZ, et al. Comparative in vitro bioequivalence evaluation of different brands of amoxicillin capsules marketed in Tigray, Ethiopia. Int J Pharm Sci Nanotech 2013; 6(1): 1966-1971.
3. Mukhtar MD, Maryam IA, Adoum AO. Quality assessment of some brands of ampicillin oral formulations on sale in some parts of Kano by thin layer chromatography and microbiological techniques. Journal of Research in Biosciences. 2006; 2(1).
4. Secco G, Sachetti C, Rossato-Grando LG, et al. Quality of vancomycin for injection formulations in Brazil. Current Pharmaceutical Analysis 2019; 15(3): 280-285
5. Tariq S, Rasheed H, Rasheed MA, Ashraf M 2012, 'Quality evaluation of different brands of ceftriaxone', M.Phil Thesis, University of Veterinary and Animal Sciences, Lahore, Pakistan.
6. Usman S, Alam A, Suleiman R, et al. Evaluation of dissolution testing for ciprofloxacin (500mg) tablets: post market surveillance of different brands available in Ras al Khaimah (UAE). Int J Biopharm 2014; 5: 65-72.

Study reported in other languages not included in the review

7. Bonfilio R, Santos OMM, Novaes ZR, et al. Controle de qualidade físico-químico e microbiológico em 2347 amostras manipuladas em 2010 e 2011. Revista de ciências farmacêuticas básica e aplicada 2013; 34(4): 527-535.
8. Cao FQ, Li D, Yan ZY. Determination of Norfloxacin by its enhancement effect on the fluorescence intensity of functionalized CdS nanoparticles. Guang Pu Xue Yu Guang Pu Fen Xi 2009; 29(8): 2222-2226.
9. Du Y. Study on the determination of metronidazole and amoxicillin in Bijiaxilin tablet by HPLC. Chinese Pharmaceutical Journal 2001; 36(2): 115-117.
10. Kan J, Qu J. HPLC determination of the contents in compound roxithromycin tablets. Chinese Journal of New Drugs 2009; I2: 171-173.
11. Li N, Shen J, Jia Y, et al. The inspection of amoxicillin medicines studied by the terahertz time-domain spectroscopy technique. Spectroscopy and Spectral Analysis 2007; 27(9): 1692-1695.
12. Lei DQ, Feng YC, Hu CQ. Using Near-infrared Spectroscopy correlation coefficient method monitoring drug quality in the circulation field. Chinese Pharmaceutical Journal 2010; 45(14): 1097-1103.

13. Liu L. Improvement of the TLC method of limit test for related substances in doxycycline hydrochloride. Chinese Journal of Pharmaceutical Analysis 1993; 13(5): 318-320.
14. Mei D, Du XL, Li DK. Pharmaceutical evaluation of different sterile ceftriaxone sodium products. Chinese Pharmaceutical Journal 2004; 39(6): 463-466.
15. Mei D, Zhao W, Fu Q, et al. Quality evaluation of different cefradine capsules. Chinese Journal of Antibiotics 2002; 27(1): 31-32, 62.
16. Shao-liang C, Lie L. Thermal analysis of the quality of chloramphenicol tablets. Chinese Journal of Antibiotics 2004; 29(1): 26-28.
17. Wu Y, Guo CM, Zhang SQ. Quality control of spectinomycin hydrochloride by HPLC/ELSD. Journal of China Pharmaceutical University 2005; 36(1):40-43.
18. Xie J, Zhang Y, Zeng Z, et al. Determination of clindamycin liposome by HPLC. Pharmaceutical Biotechnology 2008; 15(4): 299-301.
19. Yu T, Zhao RS, Zhou Y, et al. Quality evaluation of vancomycin hydrochloride for injection. Chinese Pharmaceutical Journals 2009; 44(21): 1662-1665.
20. Zarapkar SS, Halkar UP, Rane SH. Reverse phase high-performance liquid chromatographic determination of Ampicillin and Probenecid in capsules. Indian Drugs 200; 37(4): 200-203.
21. Zhang ZF, Yang GL, Liang GJ, et al. Quality study of cefoperazone sodium and sulbactam sodium for injection by HPLC. Chinese Pharmaceutical Journal 2003; 38(6): 462-464.
