# Supplementary material for: Substandard and falsified antibiotics: neglected drivers of antimicrobial resistance?
Source: BMJ Glob Health. 2022 Aug 18;7(8):e008587. doi: 10.1136/bmjgh-2022-008587 (PMC9394205; doi:10.1136/bmjgh-2022-008587)
Supplement: Supplementary data [file bmjgh-2022-008587supp006.pdf]

Substandard and falsified antibiotics: neglected drivers of antimicrobial resistance?

Supplementary file 6. Main characteristics of prevalence surveys of antibiotic quality in order of date of publication

Reference	Journal/Publisher	Sampling method	Countries	Active Pharmaceutical Ingredients (API) Sampled	Data points	Failed samples n/N (%)
**Iwuagwu, 1992[1]**	International Journal of Pharmacy Practice	Convenience	Nigeria	Ampicillin	1	3/13 (23.1 %)
**Muritu, 1994[2]**	Journal of Pharmaceutical and Biomedical Analysis	Convenience	Kenya	Tetracycline	1	0/50 (0 %)
**Okeke, 1995[3]**	International Journal of Antimicrobial Agents	Convenience	Nigeria	Tetracycline	1	6/7 (85.7 %)
**Taylor, 1995[4]**	The Lancet	Convenience	Nigeria	Amoxicillin	1	5/9 (55.6 %)
**Reseau Medicament et Developpement, 1995[5]**	The World Health Organization	Convenience	Cameroon, Chad, Madagascar	Amoxicillin, Ampicillin, Chloramphenicol, Metronidazole, Penicillin-unspecified, Sulfamethoxazole-Trimethoprim, Tetracycline	21	47/180 (26.1 %)
**Nazerali, 1996[6]**	WHO Action Programme on Essential Drugs	Convenience	Zimbabwe	Amoxicillin, Ampicillin, Benzylpenicillin (penicillin G), Doxycycline, Phenoxymethylpenicillin (penicillin V), Tetracycline	13	9/299 (3.0%)
**Gimenez, 1997[7]**	Medecine et Maladies Infectieuses	Convenience	Cambodia	Amoxicillin, Chloramphenicol, Metronidazole, Sulfamethoxazole-Trimethoprim, Tetracycline	5	23/82 (28.0 %)
**Shakoor, 1997[8]**	Tropical Medicine and International Health	Convenience	Nigeria, Thailand	Amoxicillin, Ampicillin-Cloxacillin, Sulfamethoxazole-Trimethoprim, Tetracycline	12	21/59 (35.6 %)
**Kibwage, 1998[9]**	East and Central African Journal of Pharmaceutical Sciences	Convenience	Kenya	Sulfamethoxazole-Trimethoprim	2	14/37 (37.8 %)
**Nazerali, 1998[10]**	The British Medical Journal (BMJ)	Convenience	Zimbabwe	Ampicillin	1	2/10 (20.0 %)
**Wondemagegnehu, 1999[11]**	The World Health Organization	Random	Myanmar, Viet Nam	Amoxicillin, Ampicillin, Chloramphenicol, Metronidazole, Sulfamethoxazole-Trimethoprim, Tetracycline	15	46/313 (14.7 %)
**Kamau, 2001[12]**	East and Central African Journal of Pharmaceutical Sciences	Convenience	Kenya	Ampicillin	1	11/45 (24.4 %)
**Taylor, 2001[13]**	The Lancet	Random	Nigeria	Amoxicillin, Ampicillin, Ampicillin-Cloxacillin, Benzylpenicillin (penicillin G), Cloxacillin, Doxycycline, Metronidazole, Streptomycin, Sulfamethoxazole-Trimethoprim	9	136/273 (49.8 %)
**Kolawole, 2002[14]**	Nigerian Journal of Pharmaceutical Science	Convenience	Nigeria	Ampicillin	1	14/31 (45.2%)

**Prazuck, 2002[15]**	Sexually Transmitted Diseases	Combination *	Myanmar	Benzylpenicillin (penicillin G), Ceftriaxone, Chlortetracycline, Ciprofloxacin, Doxycycline, Erythromycin, Sulfamethoxazole-Trimethoprim	7	7/19 (36.8 %)
**Sow, 2002[16]**	International Journal of Infectious Disease	Convenience	Senegal	Ampicillin, Oxytetracycline, Phenoxymethylpenicillin (penicillin V), Sulfamethoxazole-Trimethoprim	4	27/47 (57.4 %)
**Ahmed, 2003[17]**	Journal of Biological Sciences	Random	Bangladesh	Metronidazole	1	9/40 (22.5%)
**Kamau, 2003[18]**	East and Central African Journal of Pharmaceutical Sciences	Convenience	Kenya	Amoxicillin	1	4/57 (7.0 %)
**Risha, 2003[19]**	Ghent University Faculty of Pharmaceutical Sciences	Convenience	Tanzania	Amoxicillin, Ciprofloxacin, Metronidazole, Sulfamethoxazole-Trimethoprim	4	2/25 (8.0 %)
**Basco, 2004[20]**	The American Journal of Tropical Medicine and Hygiene	Convenience	Cameroon	Sulfamethoxazole-Trimethoprim	1	0/1 (0.0 %)
**Kayumba, 2004[21]**	Journal of Clinical Pharmacy and Therapeutics	Convenience	Rwanda, Tanzania	Amoxicillin, metronidazole, sulfamethoxazole-trimethoprim	6	4/25 (16.0%)
**Syhakhang, 2004[22]**	Pharmaceutical World Science	Random	Lao People's Democratic Republic	Ampicillin, Tetracycline	4	124/356 (34.8 %)
**Legris, 2005[23]**	Universite Henri Poincare – Nancy I	Random	Ivory Coast	Ampicillin, amoxicillin, metronidazole, sulfamethoxazole-trimethoprim	4	33/65 (50.8%)
**Phanouvong, 2005[24]**	United States Pharmacopeia (USP)	Convenience	Cambodia, Lao People's Democratic Republic, Thailand	Tetracycline	3	3/168 (1.8 %)
**Weir, 2005[25]**	British Journal of Ophthalmology	Convenience	India	Ciprofloxacin	1	30/30 (100.0 %)
**Lon, 2006[26]**	Transactions of the Royal Society of Tropical Medicine and Hygiene	Convenience	Cambodia	Tetracycline	1	34/128 (26.6 %)
**Obodozie, 2006[27]**	Journal of Clinical Pharmacy and Therapeutics	Convenience	Nigeria	Ampicillin-Cloxacillin	2	23/40 (57.5 %)
**Vijaykadga, 2006[28]**	The Southeast Asian Journal of Tropical Medicine and Public Health	Convenience	Thailand	Tetracycline	1	0/19 (0.0 %)
**Okoro, 2007[29]**	Research Journal of Pharmacology	Convenience	Nigeria	Ampicillin	1	5/10 (50.0 %)
**Sheth, 2007[30]**	South East Asian FIP-WHO Forum of Pharmaceutical Associations	Convenience	India	Amikacin, Amoxicillin, Amoxicillin-Clavulanic acid, Ampicillin-Cloxacillin, Cefadroxil, Cefalexin, Cefotaxime, Ceftazidime, Cefuroxime, Ciprofloxacin, Erythromycin, Gentamicin, Roxithromycin	14	8/195 (4.1%)

**Camara, 2008[31]**	Ministere de l'Education Nationale, Universite de Bamako	Convenience	Mali	Erythromycin, gentamicin, tetracycline	3	6/30 (20.0%)
**Kyriacos, 2008[32]**	Journal of Clinical Pharmacy and Therapeutics	Random	Egypt, Jordan, Lebanon, Saudi Arabia	Amoxicillin	5	14/32 (43.8 %)
**Meos, 2008[33]**	Drug Development Research	Convenience	Estonia, Russian Federation	Doxycycline	2	2/8 (25.0 %)
**Pouillot, 2008[34]**	Bulletin de la Societe de Pathologie exotique	Convenience	Cameroon, Niger	Amoxicillin, Ampicillin, Chloramphenicol, Gentamicin, Metronidazole, Norfloxacin, Oxacillin, Sulfamethoxazole-Trimethoprim, Tetracycline	11	45/102 (44.1 %)
**Bate, 2009[35]**	PLoS ONE	Convenience	India	Ciprofloxacin, Erythromycin	4	17/220 (7.8 %)
**Central Drug Standard Control Organisation, 2009[36]**	Central Drugs Standard Control Organisation CDSCO, India	Convenience	India	Amoxicillin, Ciprofloxacin, Sulfamethoxazole-Trimethoprim	3	0/165 (0.0 %)
**Diop, 2009[37]**	Medecine Tropicale	Unknown	Senegal	Amoxicillin, Ampicillin, Ciprofloxacin, Erythromycin, Phenoxymethylpenicillin (penicillin V)	5	10/34 (29.4 %)
**Mak, 2009[38]**	Universite de la mediterranee aix-marseille II	Random	Cambodia	Amoxicillin	6	3/39 (7.7%)
**Obaid, 2009[39]**	Pakistan Journal of Pharmaceutical Sciences	Convenience	Pakistan	Ceftriaxone	1	16/96 (16.7 %)
**Zaheer, 2009[40]**	Pakistan Journal of Scientific and Industrial Research	Unknown	Pakistan	Ciprofloxacin, Levofloxacin, Ofloxacin	3	1/12 (8.3 %)
**Hadi, 2010[41]**	BMC Infectious Diseases	Convenience	Indonesia	Amoxicillin, Chloramphenicol, Ciprofloxacin, Sulfamethoxazole-Trimethoprim, Tetracycline	5	19/104 (18.3 %)
**Khan, 2010[42]**	Tropical Medicine and International Health	Random	Cambodia	Metronidazole	1	1/79 (1.3 %)
**Bate, 2011[43]**	Journal of Health Economics	Convenience	Democratic Republic of the Congo	Ciprofloxacin, Erythromycin	2	34/304 (11.2 %)
**Haider, 2011[44]**	Journal of Applied Pharmaceutical Science	Convenience	Bangladesh	Amoxicillin	1	3/10 (30.0 %)
**Idries, 2011[45]**	University of Khartoum	Convenience	Sudan	Amoxicillin, Ceftriaxone, Ciprofloxacin, Metronidazole	4	1/11 (9.1 %)
**Kamuhabwa, 2011[46]**	International Journal of Pharmaceutical Research	Convenience	Tanzania	Sulfamethoxazole-Trimethoprim	1	4/17 (23.5 %)
**Nair, 2011[47]**	Journal of Pharmaceutical Sciences	Random	Papua New Guinea	Amoxicillin	1	8/8 (100.0 %)
**Seear, 2011[48]**	Journal of Clinical Pharmacy and Therapeutics	Convenience	India	Ciprofloxacin	1	9/100 (9.0 %)

**World Health Organization, 2011[49]**	The World Health Organization	Convenience	Armenia, Azerbaijan, Belarus, Kazakhstan, Ukraine, Uzbekistan	Ofloxacin	6	6/50 (12.0 %)
**Yusuf, 2011[50]**	Management Sciences for Health		Afghanistan	Amoxicillin, Ceftriaxone, Ciprofloxacin, Gentamicin	4	5/125 (4.0%)
**Karlage, 2012[51]**	Drug Development and Industrial Pharmacy	Random	Mexico, United States	Amoxicillin, Ampicillin, Ciprofloxacin, Sulfamethoxazole-Trimethoprim	6	7/11 (63.6 %)
**Patel, 2012[52]**	BMC Health Services Research	Convenience	South Africa	Amoxicillin	3	0/46 (0 %)
**Akinkunmi, 2013[53]**	Annals of Tropical Medicine and Parasitology	Convenience	Nigeria	Chloramphenicol, Gentamicin	2	0/2 (0 %)
**Khan, 2013[54]**	BMC Pharmacology and Toxicology	Random	Cambodia	Amoxicillin-Clavulanic acid	1	26/59 (44.1 %)
**Khojah, 2013[55]**	Pharmacology & Pharmacy	Lot Quality Assurance Sampling	Saudi Arabia	Amoxicillin	1	9/83 (10.8%)
**Phanouvong, 2013a[56]**	The Southeast Asian Journal of Tropical Medicine and Public Health	Random	Thailand	Tetracycline	1	1/242 (0.4 %)
**Phanouvong, 2013b** [57]**	The Southeast Asian Journal of Tropical Medicine and Public Health	Random	Cambodia	Tetracycline	1	unknown (5.0%)
**Ramachandran, 2013[58]**	Tropical Medicine and International Health	Convenience	India	Levofloxacin	1	9/67 (13.4 %)
**Food and Drug Quality Control Center (Lao PDR), 2014[59]**	Ministry of Health, Lao PDR	Random	Lao PDR	Amoxicillin, Cloxacillin	11	0/15 (0%)
**Hetzel, 2014[60]**	PLoS ONE	Random	Papua New Guinea	Amoxicillin, Doxycycline	2	1/59 (1.7 %)
**Khuluza, 2014[61]**	Malawi Medical Journal	Random	Malawi	Sulfamethoxazole-Trimethoprim	1	5/11 (45.4 %)
**Khurelbat, 2014[62]**	SpringerPlus	Random	Mongolia	Amoxicillin, Ampicillin, Doxycycline, Metronidazole, Sulfamethoxazole-Trimethoprim	10	55/699 (7.8 %)
**Nagaraj, 2014[63]**	Journal of Research in Pharmacy Practice	Convenience	India	Amoxicillin, Cefuroxime, Ofloxacin	3	0/10 (0.0 %)
**Yoshida, 2014[64]**	BMC Pharmacology and Toxicology	Random	Cambodia	Cefixime, Clarithromycin, Sulfamethoxazole-Trimethoprim	3	32/190 (16.8 %)

**Bate, 2015[65]**	Journal of Economics & Management Strategy	Convenience	Angola, Brazil, China, Democratic Republic of the Congo, Egypt, Ethiopia, Ghana, India, Kenya, Mozambique, Nigeria, Russia, Rwanda, Tanzania, Thailand, Turkey, Uganda, Zambia	Ciprofloxacin	1	142/1437 (9.9 %)
**Boadu, 2015[66]**	Medicinal Chemistry	Convenience	Ghana	Amoxicillin, Cloxacillin, Flucloxacillin	3	23/54 (42.6 %)
**Fadeyi, 2015[67]**	The American Journal of Tropical Medicine and Hygiene	Convenience	Ghana, Nigeria, United Kingdom	Amoxicillin, Sulfamethoxazole-Trimethoprim	6	15/35 (42.8 %)
**Tshilumba, 2015[68]**	Pan African Medical Journal	Convenience	Democratic Republic of the Congo	Amoxicillin, Ampicillin, Ciprofloxacin, Metronidazole	4	14/47 (29.8 %)
**Wang, 2015[69]**	Journal of Clinical Pharmacy and Therapeutics	Convenience	China, Ethiopia, Lao People's Democratic Republic, Mexico, Nigeria, South Africa, Thailand, United States	Sulfamethoxazole-Trimethoprim	10	2/42 (4.8 %)
**World Health Organization, 2015[70]**	The World Health Organization	Convenience	Burkina Faso, Kenya, Madagascar, Nepal, Nigeria, Tajikistan, Tanzania, Uganda, Viet Nam, Zimbabwe	Amoxicillin, Ampicillin, Ceftriaxone, Gentamicin, Procaine benzylpenicillin (penicillin G)	49	23/106 (21.7 %)

**Kaale, 2016[71]**	PLoS ONE	Random	Tanzania	Amoxicillin, Sulfamethoxazole-Trimethoprim	2	0/124 (0 %)
**Khan, 2016[72]**	International Journal of Pharmacy and Pharmaceutical Sciences	Convenience	India	Amoxicillin	1	13/46 (28.3 %)
**Nga, 2016[73]**	International Journal of Innovation and Applied Studies	Random	Cameroon	Sulfamethoxazole-Trimethoprim	3	27/37 (73 %)
**Osei-Safo, 2016[74]**	International Journal of Pharmaceutical Sciences and Research	Convenience	Ghana, Nigeria	Azithromycin, Clindamycin, Erythromycin	6	36/45 (80.0 %)
**Tshilumba, 2016[75]**	International Journal of Pharmacy and Pharmaceutical Sciences	Random	Democratic Republic of the Congo	Metronidazole	1	4/15 (26.7 %)
**Wafula, 2016[76]**	Drugs - Real World Outcomes	Convenience	Kenya	Amoxicillin, Amoxicillin-Clavulanic acid, Erythromycin, Metronidazole, Sulfamethoxazole-Trimethoprim	5	2/21 (9.5 %)
**Alhedethe, 2017[77]**	Archives in Chemical Research	Convenience	Sierra Leone	Amoxicillin	1	0/9 (0.0%)
**Bate, 2017[78]**	Safe Medicines Coalition	Convenience	The Internet	Ciprofloxacin	1	11/244 (4.5 %)
**Islam, 2017[79]**	Journal of International Health	Convenience	Cambodia	Ceftriaxone, Cefuroxime, Ciprofloxacin, Clarithromycin, Gentamicin, Levofloxacin, Nalidixic acid, Ofloxacin, Phenoxymethylpenicillin (penicillin V), Roxithromycin, Sulfamethoxazole-Trimethoprim	11	115/590 (19.5 %)
**Islam, 2017[80]**	Graduate School of Medical Science & Technology Kanazawa University	Convenience	Myanmar	Ceftriaxone, Cefuroxime, Gentamicin	3	37/167 (22.2 %)
**Khuluza, 2017[81]**	The American Journal of Tropical Medicine and Hygiene	Random	Malawi	Amoxicillin, Amoxicillin-Clavulanic acid, Cefuroxime, Chloramphenicol, Ciprofloxacin, Phenoxymethylpenicillin (penicillin V)	6	3/23 (13.0 %)
**Nabirova, 2017[82]**	The International Journal of Tuberculosis and Lung Disease	Random	Kazakhstan	Amoxicillin-Clavulanic acid, Levofloxacin, Moxifloxacin, Ofloxacin	4	1/8 (12.5 %)
**Ononna, 2017[83]**	Bangladesh Medical Research Council Bulletin	Random	Bangladesh	Amoxicillin, Azithromycin, Cefixime, Cephradine, Ciprofloxacin, Metronidazole	6	36/39 (92.3 %)
**Petersen, 2017[84]**	PLoS ONE	Convenience	Democratic Republic of the Congo	Amoxicillin, Amoxicillin-Clavulanic acid, Ampicillin, Ampicillin-Cloxacillin, Azithromycin, Cefalexin, Cefixime, Ceftriaxone, Cefuroxime, Chloramphenicol, Ciprofloxacin, Clarithromycin, Cloxacillin, Doxycycline, Erythromycin, Levofloxacin, Metronidazole, Moxifloxacin, Ofloxacin, Phenoxymethylpenicillin (penicillin V), Sulfamethoxazole, Sulfamethoxazole-Trimethoprim, Tetracycline	23	5/426 (1.2 %)

**Bate, 2018[85]**	The B.E. Journal of Economic Analysis & Policy	Convenience	Argentina, Bolivia, Brazil, Ecuador, Guatemala, Honduras, Paraguay, Peru, Uruguay, Venezuela	Ciprofloxacin	10	48/687 (7.0 %)
**Chikowe, 2018[86]**	The American Journal of Tropical Medicine and Hygiene	Convenience	Malawi	Amoxicillin	1	0/42 (0.0 %)
**Ernest, 2018[87]**	International Journal of Recent Innovations in Academic Research	Convenience	Cameroon	Metronidazole	3	2/10 (20.0%)
**Frimpong, 2018[88]**	Journal of Tropical Medicine	Random	Ghana	Amoxicillin, Cefuroxime, Ciprofloxacin, Flucloxacillin, Metronidazole, Sulfamethoxazole-Trimethoprim	6	21/29 (72.4 %)
**Joda, 2018[89]**	lfe Journal of Science	Random	Nigeria	Ciprofloxacin	1	4/16 (25.0 %)
**Lehmann, 2018a[90]**	Journal of Pharmaceutical Sciences	Random	South Africa	Amoxicillin, Amoxicillin-Clavulanic acid	2	25/138 (18.1 %)
**Lehmann, 2018b[91]**	Journal of Pharmacy and Pharmacology	Convenience	Germany, South Africa	Amoxicillin-Clavulanic acid	2	0/2 (0.0 %)
**Nga, 2018[92]**	Health Sciences and Diseases	Random	Cameroon	Sulfamethoxazole-trimethoprim	2	21/81 (25.9%)
**Schafermann, 2018[93]**	PLoS ONE	Convenience	Togo	Amoxicillin, Amoxicillin-Clavulanic acid, Ciprofloxacin, Doxycycline, Metronidazole, Phenoxymethylpenicillin (penicillin V), Sulfamethoxazole-Trimethoprim	25	6/64 (9.4 %)
**Schiavetti, 2018[94]**	The American Journal of Tropical Medicine and Hygiene	Random	Democratic Republic of the Congo	Amoxicillin	1	9/80 (11.2 %)
**Tshilombo, 2018[95]**	American Journal of Analytical Chemistry	Convenience	Democratic Republic of the Congo	Amoxicillin, Amoxicillin-Clavulanic acid	2	55/200 (27.5%)
**Lawal, 2019[96]**	Asian Journal of Pharmaceutical Research and Development	Convenience	Nigeria	Ampicillin-Cloxacillin, Amoxicillin, Amoxicillin-Clavulanic acid, Ciprofloxacin, Metronidazole, Sulfamethoxazole-Trimethoprim	6	43/112 (38.4%)
**Myers, 2019[97]**	Analytical Methods	Convenience	Kenya	Amoxicillin, Amoxicillin-Clavulanic acid, Ampicillin	3	46/189 (24.3 %)

**Scrimgeour, 2019[98]**	Journal of Pharmacy Practice and Research	Convenience	Papua New Guinea, Solomon Islands, Vanuatu	Amoxicillin, Ampicillin, Benzylpenicillin (penicillin G), Ceftriaxone, Cloxacillin, Doxycycline, Erythromycin, Flucloxacillin, Metronidazole, Phenoxymethylpenicillin (penicillin V), Sulfamethoxazole-Trimethoprim	11	6/33 (18.2 %)
**Tabernero, 2019[99]**	Journal of Antimicrobial Chemotherapy	Random	Lao People's Democratic Republic	Amoxicillin, Ampicillin, Ceftriaxone, Ciprofloxacin, Doxycycline, Ofloxacin, Sulfamethoxazole-Trimethoprim, Tetracycline	8	129/905 (14.3%)
**Bekoe, 2020[100]**	Tropical Medicine and International Health	Convenience	Ghana	Ampicillin, Amoxicillin, Amoxicillin-Clavulanic acid, Benzylpenicillin, Cefuroxime, Ceftriaxone, Ciprofloxacin, Erythromycin, Gentamicin, Metronidazole, Phenoxymethylpenicillin, Sulfamethoxazole-Trimethoprim, Tetracycline	13	231/342 (67.5%)
**Hand, 2020[101]**	Pharmacology Research and Perspectives	Unknown	Unknown	Benzathine Benzylpenicillin	1	0/35 (0.0%)
**Husaini, 2020[102]**	PLoS ONE	Random	Belize	Amoxicillin, Ciprofloxacin, Sulfamethoxazole-Trimethoprim	3	12/17 (70.6%)
**Jean-Baptiste, 2020[103]**	MDPI	Random	Haiti	Amoxicillin, Amoxicillin-Clavulanic acid, Azithromycin, Ciprofloxacin, Clarithromycin, Cloxacillin, Erythromycin, Metronidazole, Sulfamethoxazole-Trimethoprim, Tetracycline	10	45/180 (25.0%)
**Khurelbat, 2020[104]**	BMC Public Health	Random	Mongolia	Amoxicillin, Ciprofloxacin, Metronidazole	3	47/354 (13.3%)
**Koech, 2020[105]**	Hindawi BioMed Research International	Convenience	Kenya	Amoxicillin, Amoxicillin-Clavulanic acid	2	4/53 (7.6%)
**Sakuda, 2020[106]**	Pharmacy (MDPI)	Unknown	Myanmar	Ciprofloxacin, Levofloxacin	2	20/86 (23.3%)
**Schafermann, 2020[107]**	The Journal of Tropical Medicine and Hygiene	Random	Cameroon, Democratic Republic of the Congo	Amoxicillin, Amoxicillin-Clavulanic acid, Ciprofloxacin, Doxycycline, Metronidazole, Phenoxymethylpenicillin, Sulfamethoxazole-Trimethoprim	14	45/348 (12.9%)
*** Combination of convenience and randomized**						
**** Number of samples unknown**						

References

1 Iwuagwu MA, Onyeonwu N. In vitro assessment of ampicillin capsules marketed in Nigeria. *Int J Pharm Pract* 1992;**1**:167–71.

2 Muritu JW, Kibwage IO, Maitai CK, *et al.* Evaluation of tetracycline raw materials and finished products found on the Kenyan market. *J Pharm Biomed Anal* 1994;**12**:1483–8. doi:10.1016/0731-7085(94)00105-7

3 Okeke IN, Lamikanra A. Quality and bioavailability of tetracycline capsules in a Nigerian semi-urban community. *Int J Antimicrob Agents* 1995;**5**:245–50. doi:10.1016/0924-8579(94)00064-2

4 Taylor RB, Shakoor O, Behrens RH. Drug quality, a contributor to drug resistance? *Lancet* 1995;**346**:122.

5 Reseau Medicament et Developpement. La qualite des medicaments sur le marche pharmaceutique africain: etude analytique dans trois pays - Cameroun, Madagascar, Tchad. Geneva: 1995.

6 Nazerali H, Muchemwa T, Hogerzeil H. Stability of essential drugs in tropical climates (Zimbabwe). Geneva: 1996. http://apps.who.int/medicinedocs/pdf/s2206e/s2206e.pdf

7 Gimenez F, Bruneton C, Narong Rith DY. Etude de la qualite des medicaments vendus et dispenses au Cambodge. *Med Mal Infect* 1997;**27**:541–4.

8 Shakoor O, Taylor RB, Behrens RH. Assessment of the incidence of substandard drugs in developing countries. *Trop Med Int Heal* 1997;**2**:839–45. doi:10.1046/j.1365-3156.1997.d01-403.x

9 Kibwage IO, Ondari CO, Mureithi IG, *et al.* Analysis of Co-trimoxazole products on the Kenyan market. *East Cent African J Pharm Sci* 1998;**1**:34–8.

10 Nazerali H, Hogerzeil H V. The quality and stability of essential drugs in rural Zimbabwe: controlled longitudinal study. *BMJ* 1998;**317**:512–3. doi:10.1136/bmj.317.7157.512

11 Wondemagegnehu E. Counterfeit and Substandard Drugs in Myanmar and Viet Nam. Geneva: 1999. doi:10.1016/j.scispo.2006.06.006

12 Kamau FN, Thoithi GN, Kibwage IO. Quality of Ampicillin Preparations on the Kenyan Market. *East Cent African J Pharm Sci* 2001;**4**:25–9.

13 Taylor RB, Shakoor O, Behrens RH, *et al.* Pharmacopoeial quality of drugs supplied by Nigerian pharmacies. *Lancet* 2001;**357**:1933–6.

14 Kolawole JA, Olorunfemi PO, Okeniyi SO, *et al.* Quality and availability of ampicillin products (capsules and suspensions) in Nigeria. *Niger J Pharm* 2002;**33**:27–41.

15 Prazuck T, Falconi I, Morineau G, *et al.* Quality control of antibiotics before the implementation of an STD program in Northern Myanmar. *Sex Transm Dis* 2002;**29**:624–7. doi:10.1097/00007435-200211000-00002

16 Sow PS, Gueye TSN, Sy E, *et al.* Drugs in the parallel market for the treatment of urethral discharge in Dakar: epidemiologic investigation and physicochemical tests. *Int J Infect Dis* 2002;**6**:108–12. doi:10.1016/s1201-9712(02)90070-6

17 Ahmed F, Das AK, Karmakar UK, *et al.* Quality of marketed metronidazole preparations in Bangladesh - An analytical overview. *J Biol Sci* 2003;**3**:940–50.

18 Kamau FN, Thoithi G., Ngugi JK, *et al.* Quality of amoxycillin preparations on the Kenyan market. *East Cent African J Pharm Sci* 2003;**6**:57–60.

19 Risha PG. *Quality of essential drugs on the Tanzanian market: influence of tropical climate on in vitro dissolution and bioavailability*. 2003.

20 Basco LK. Molecular epidemiology of malaria in Cameroon. XIX. Quality of antimalarial drugs used for self-medication. *Am J Trop Med Hyg* 2004;**70**:245–50.

21 Kayumba PC, Risha PG, Shewiyo D, *et al.* The quality of essential antimicrobial and antimalarial drugs marketed in Rwanda and Tanzania: influence of tropical storage conditions on in vitro dissolution. *J Clin Pharm Ther* 2004;**29**:331–8. doi:10.1111/j.1365-2710.2004.00568.x

22 Syhakhang L, Lundborg CS, Lindgren B, *et al.* The quality of drugs in private pharmacies in Lao PDR: a repeat study in 1997 and 1999. *Pharm World Sci* 2004;**26**:333–8. doi:10.1007/s11096-004-0558-3

23 Legris C. *La détection des médicaments contrefaits par investigation de leur authenticité: Étude pilote sur le marché pharmaceutique illicite de Côte d'Ivoire*. 2005.

24 Phanouvong S, Reiss S, Smine A. Why be concerned about the quality of antimalarial and ARV drugs? Maryland: : United States Pharmacopeia 2005.

25 Weir RE, Zaidi FH, Charteris DG, *et al.* Variability in the content of Indian generic ciprofloxacin eye drops. *Br J Ophthalmol* 2005;**89**:1094–6. doi:10.1136/bjo.2004.059519

26 Lon CT, Tsuyuoka R, Phanouvong S, *et al.* Counterfeit and substandard antimalarial drugs in Cambodia. *Trans R Soc Trop Med Hyg* 2006;**100**:1019–24. doi:10.1016/j.trstmh.2006.01.003

27 Obodozie OO, Mustapha KB, Ebeshi BU, *et al.* A comparative study on the prevalence of substandard ampicillin/cloxacillin preparations in the Nigerian market: Mid 1990's and present. *J Phytomedicine Ther* 2006;**11**:1–8. doi:10.4314/jopat.v11i1.48619

28 Vijaykadga S, Cholpol S, Sitthimongkol S, *et al.* Strengthening of national capacity in implementation of antimalarial drug quality assurance in Thailand. *Southeast Asian J Trop Med Public Health* 2006;**37 Suppl 3**:5–10.

29 Okoro IA, Ejike EN. Quality control assay of ampicillin brands of capsules in Onitsha market, Southeastern Nigeria. *Res J Pharmacol* 2007;**1**:9–11.

30 Sheth PD, Siva Prasada Reddy M V, Regal B, *et al.* Extent of Spurious (Counterfeit) Medicines in India. New Delhi: 2007.

31 Camara I. *Étude de la qualité des médicaments génériques DCI achetés par la Pharmacie Populaire du Mali dans le cadre des appels d'offres de 2002 à 2005*. 2008.

32 Kyriacos S, Mroueh M, Chahine RP, *et al.* Quality of amoxicillin formulations in some Arab countries. *J Clin Pharm Ther* 2008;**33**:375–9. doi:10.1111/j.1365-2710.2008.00926.x

33 Meos A, Paap M, Matto V, *et al.* Comparative evaluation of quality of doxycycline formulations registered in Estonia to those registered in the Russian Federation. *Drug Dev Res* 2008;**69**:58–68. doi:10.1002/ddr.20228

34 Pouillot R, Bilong C, Boisier P, *et al.* Le circuit informel des médicaments à Yaoundé et à Niamey : étude de la population des vendeurs et de la qualité des médicaments distribués. *Bull la Soc Pathol Exot* 2008;**101**:113–8.

35 Bate R, Tren R, Mooney L, *et al.* Pilot study of essential drug quality in two major cities in India. *PLoS One* 2009;**4**:e6003. doi:10.1371/journal.pone.0006003

36 Central Drugs Standard Control Organization. Report on countrywide survey for spurious drugs. New Delhi: 2009.

37 Diop A, Sarr SO, Diop YM, *et al.* Contrôle de la qualité de quelques molécules antibiotiques utilisées au Sénégal. *Med Trop* 2009;**69**:251–4.

38 Mak S. *Evaluation de la qualite de l'amoxicilline au Cambodge*. 2009.

39 Obaid A. Quality of ceftriaxone in Pakistan: reality and resonance. *Pak J Pharm Sci* 2009;**22**:220–9.

40 Zaheer M, Rahman S, Mahmood S, *et al.* In vitro analysis and data comparison of market brands of ciprofloxacin, ofloxacin and levofloxacin. *Pak J Sci Ind Res* 2009;**52**:186–90.

41 Hadi U, van den Broek P, Kolopaking EP, *et al.* Cross-sectional study of availability and pharmaceutical quality of antibiotics requested with or without prescription (Over The Counter) in Surabaya, Indonesia. *BMC Infect Dis* 2010;**10**:1–10. doi:10.1186/1471-2334-10-203

42 Khan MH, Okumura J, Sovannarith T, *et al.* Prevalence of counterfeit anthelminthic medicines: A cross-sectional survey in Cambodia. *Trop Med Int Heal* 2010;**15**:639–44. doi:10.1111/j.1365-3156.2010.02494.x

43 Bate R, Zhe Jin G, Mathur A. Does price reveal poor-quality drugs? Evidence from 17 countries. Massachusetts: : National Bureau of Economic Research 2011. doi:10.1016/j.jhealeco.2011.08.006

44 Haider SS, Nasrin N, Apu AS, *et al.* Accelerated stability and antimicrobial sensitivity studies of amoxicillin dry suspensions marketed in Bangladesh. *J Appl Pharm Sci* 2011;**01**:51–5.

45 Idries A. Incidence of substandard medicines in Khartoum, Sudan in 2009-2010: Results of medicines quality monitoring survey. Khartoum: 2011.

46 Kamuhabwa AA. R, Saifuddin MH, Maganda B, *et al.* Quality of co-trimoxazole tablets used for management of opportunistic infections in HIV/AIDS Patients. *Int J Pharm Res* 2011;**3**:79–84.

47 Nair A, Strauch S, Lauwo J, *et al.* Are counterfeit or substandard anti-infective products the cause of treatment failure in Papua New Guinea? *J Pharm Sci* 2011;**100**:5059–68. doi:10.1002/jps.22691

48 Seear M, Gandhi D, Carr R, *et al.* The need for better data about counterfeit drugs in developing countries: a proposed standard research methodology tested in Chennai, India. *J Clin Pharm Ther* 2011;**36**:488–95. doi:10.1111/j.1365-2710.2010.01198.x

49 World Health Organization. Survey of the quality of anti-tuberculosis medicines circulating in selected newly independent states of the former Soviet Union. Geneva: 2011. https://digicollections.net/medicinedocs/#d/s19053en

50 Yusuf I, Lee D, Fatehzada Z, *et al.* Afghanistan Medicines Sampling and Testing – A Quantitative Survey. Virginia: 2011.

51 Karlage KL, Franklin SJ, Mufich WC, *et al.* Comparative evaluation of pharmaceutical products obtained in Mexico: Augmenting existing scientific data. *Drug Dev Ind Pharm* 2012;**38**:808–14. doi:10.3109/03639045.2011.628678

52 Patel A, Gauld R, Norris P, *et al.* Quality of generic medicines in South Africa: perceptions versus reality - a qualitative study. *BMC Health Serv Res* 2012;**12**:1–8. doi:10.1186/1472-6963-12-297

53 Akinkunmi EO. An evaluation of the pharmaceutical quality and antimicrobial effectiveness of some frequently used eye drop products available for sale in Nigeria. *Ann Trop Med Public Heal* 2013;**6**:221–6. doi:10.4103/1755-6783.116509

54 Khan MH, Hatanaka K, Sovannarith T, *et al.* Effects of packaging and storage conditions on the quality of amoxicillin-clavulanic acid - an analysis of Cambodian samples. *BMC Pharmacol Toxicol* 2013;**14**:1–7.

55 Khojah HMJ, Pallos H, Yoshida N, *et al.* The quality of medicines in community pharmacies in Riyadh, Saudi Arabia: A Lot Quality Assurance Sampling (LQAS)-based survey. *Pharmacol Pharm* 2013;**04**:511–9. doi:10.4236/pp.2013.47074

56 Phanouvong S, Dijiba Y, Vijaykadga S, *et al.* The quality of antimalarial medicines in Eastern Thailand : a case study along the Thai-Cambodian border. *Southeast Asian J Trop Med Public Health* 2013;**44**:363–73.

57 Phanouvong S, Raymond C, Krech L, *et al.* The quality of antimalarial medicines in Western Cambodia : a case study along the Thai-Cambodian border. *Southeast Asian J Trop Med Public Health* 2013;**44**:349–62.

58 Ramachandran G, Chandrasekaran V, Hemanth Kumar AK, *et al.* Estimation of content of anti-TB drugs supplied at centres of the Revised National TB Control Programme in Tamil Nadu, India. *Trop Med Int Heal* 2013;**18**:1141–4. doi:10.1111/tmi.12158

59 Food and Drug Quality Control Center. Comparative study of the quality , availability, and source of antimalarial medicines in Cambodia, Laos, Thailand, and Vietnam in PQM-MQM covered and non-covered areas in Mekong Sub-Region. Vientiane: 2014.

60 Hetzel MW, Page-Sharp M, Bala N, *et al.* Quality of Antimalarial Drugs and Antibiotics in Papua New Guinea: A Survey of the Health Facility Supply Chain. *PLoS One* 2014;**9**:e96810. doi:10.1371/journal.pone.0096810

61 Khuluza F. In-vitro evaluation of the quality of paracetamol and co-trimoxazole tablets used in Malawi based on pharmacopoeial standards. *Malawi Med J* 2014;**26**:38–41.

62 Khurelbat D, Dorj G, Bayarsaikhan E, *et al.* Prevalence estimates of substandard drugs in Mongolia using a random sample survey. *Springerplus* 2014;**3**:1–10. doi:10.1186/2193-1801-3-709

63 Nagaraj A, Tambi S, Shravani G, *et al.* Counterfeit medicines sale on online pharmacies in India. *J Res Pharm Pract* 2014;**3**:145–6. doi:10.4103/2279-042X.145397

64 Yoshida N, Khan MH, Tabata H, *et al.* A cross-sectional investigation of the quality of selected medicines in Cambodia in 2010. *BMC Pharmacol Toxicol* 2014;**15**:1–8. doi:10.1186/2050-6511-15-13

65 Bate R, Jin GZ, Mathur A. Falsified or Substandard? Assessing Price and Non-price Signals of Drug Quality. *J Econ Manag Strateg* 2015;**24**:687–711. doi:10.1111/jems.12114

66 Boadu RF, Agyare C, Adarkwa-Yiadom M, *et al.* In vitro activity and evaluation of quality of some selected penicillins on the Ghanaian market using developed HPLC methods. *Med Chem (Los Angeles)* 2015;**5**:1–14. doi:10.4172/2161-0444.1000235

67 Fadeyi I, Lalani M, Mailk N, *et al.* Quality of the antibiotics—amoxicillin and co-Trimoxazole from Ghana, Nigeria, and the United Kingdom. *Am J Trop Med Hyg* 2015;**92**:87–94. doi:10.4269/ajtmh.14-0539

68 Tshilumba PM, Amuri SB, Kaghowa ER, *et al.* Enquête sur la contrefaçon de quelques anti-infectieux administrés per os commercialisés dans la ville de Lubumbashi. *Pan Afr Med J* 2015;**22**. doi:10.11604/pamj.2015.22.318.7302

69 Wang T, Hoag SW, Eng ML, *et al.* Quality of antiretroviral and opportunistic infection medications dispensed from developing countries and internet pharmacies. *J Clin Pharm Ther* 2015;**40**:68–75. doi:10.1111/jcpt.12226

70 World Health Organization. Medicines for women and children: quality and availability of selected life-saving reproductive health medicines in developing countries. *WHO Drug Inf* 2015;**29**:324–33.

71 Kaale E, Manyanga V, Chambuso M, *et al.* The quality of selected essential medicines sold in accredited drug dispensing outlets and pharmacies in Tanzania. *PLoS One* 2016;**11**:e0165785. doi:10.1371/journal.pone.0165785

72 Khan AN, Khar RK, Udayabanu M. Quality and affordability of amoxicillin generic products: A patient concern. *Int J Pharm Pharm Sci* 2016;**8**:386–90.

73 Nga EN, Tabi Ombga YLT, Kidik Pouka MC, *et al.* Evaluation de la qualité du cotrimoxazole 480mg comprimé générique vendu dans la ville de Douala (Cameroun). *Int J Innov Appl Sci* 2016;**15**:926–35.

74 Osei-Safo D, Egbo HA, Nettey H, *et al.* Evaluation of the quality of some antibiotics distributed in Accra and Lagos. *Int J Pharm Sci Res* 2016;**7**:1991–2000. doi:10.13040/IJPSR.0975-8232.7(5).1991-00

75 Tshilumba PM, Duez P, Ndoumba JBK. Preliminary survey of counterfeiting of albendazole and metronidazole marketed in Lubumbashi. *Int J Pharm Pharm Sci*

2016;**8**:282–7.https://innovareacademics.in/journals/index.php/ijpps

76 Wafula F, Dolinger A, Daniels B, *et al.* Examining the quality of medicines at Kenyan healthcare facilities: A validation of an alternative post-market surveillance model that uses standardized patients. *Drugs - Real World Outcomes* 2016;**4**:53–63. doi:10.1007/s40801-016-0100-7

77 Alhedethe A, Alhudaithy K, Zloh M. An evaluation of prevalence of low quality of medicines in Saudi Arabia and factors associated an analytical comparative study. *Arch Chem Res* 2017;**02**:1–18. doi:10.21767/2572-4657.100014

78 Bate R. Web pharmacies and ciprofloxacin: a field study. Safe Med. Coalit. 2017;:1–2.http://safemedicinescoalition.org/ (accessed 17 Mar 2021).

79 Islam R, Yoshida N, Tsuboi H, *et al.* Four-year survey of the quality of antimicrobial medicines in Cambodia. *J Int Heal* 2017;**32**:233–42. doi:10.11197/jaih.32.233

80 Islam R. *Investigations of the quality medicines distributed in Myanmar and Cambodia, through different surveys*. 2017.

81 Khuluza F, Kigera S, Heide L. Low prevalence of substandard and falsified antimalarial and antibiotic medicines in public and faith-based health facilities of Southern Malawi. *Am J Trop Med Hyg* 2017;**96**:1124–35. doi:10.4269/ajtmh.16-1008

82 Nabirova D, Schmid G, Yusupova R, *et al.* Assessment of the quality of anti-tuberculosis medicines in Almaty, Kazakhstan, 2014. *Int J Tuberc Lung Dis* 2017;**21**:1161–8. doi:10.5588/ijtld.17.0074

83 Ononna HF, Al Hossain ASMM, Ganguly A, *et al.* Potency determination of essential drug samples manufactured by small and medium pharmaceutical industries in Bangladesh. *Bangladesh Med Res Counc Bull* 2017;**43**:87–93. doi:10.3329/bmrcb.v43i2.35195

84 Petersen A, Held N, Heide L. Surveillance for falsified and substandard medicines in Africa and Asia by local organizations using the low-cost GPHF Minilab. *PLoS One* 2017;**12**:e0184165. doi:10.1371/journal.pone.0184165

85 Bate R, Mathur A. Corruption and medicine quality in Latin America: a pilot study. *BE J Econ Anal Policy* 2018;**18**. doi:10.1515/bejeap-2017-0076

86 Chikowe I, Bliese SL, Lucas S, *et al.* Amoxicillin quality and selling practices in urban pharmacies and drug stores of Blantyre, Malawi. *Am J Trop Med Hyg* 2018;**99**:233–8. doi:10.4269/ajtmh.18-0003

87 Ernest D, Gislaine N, Michel TJ. Contrôle de qualité des imidazolés antiparasitaires distribués a Bafoussam: albendazole, mebendazole et metronidazole. *Int J Recent Innov Acad Res* 2108;**2**:79–90.

88 Frimpong G, Ofori-Kwakye K, Kuntworbe N, *et al.* Quality assessment of some essential children's medicines sold in licensed outlets in Ashanti region, Ghana. *J Trop Med* Published Online First: 2018. doi:10.1155/2018/1494957

89 Joda AE, Tayo F, Aina BA. Quality assessment of ciprofloxacin tablets obtained from community pharmacies in Lagos, Nigeria. *Ife J Sci* 2018;**20**:155–68. doi:10.4314/ijs.v20i1.16

90 Lehmann A, Katerere DR, Dressman J. Drug quality in South Africa: a field test. *J Pharm Sci* 2018;**107**:2720–30. doi:10.1016/j.xphs.2018.06.012

91 Lehmann A, Hofsäss M, Dressman J. Differences in drug quality between South Africa and Germany. *J Pharm Pharmacol* 2018;**70**:1301–14. doi:10.1111/jphp.12985

92 Nga EN, Tanga RT, Lobe VS, *et al.* Évaluation de la Stabilité du Cotrimoxazole 240 mg/5 ml Suspension Commercialisé dans les Circuits Formel et Informel de la Ville de Douala. *Heal Sci Dis* 2018;**19**:66–71.

93 Schäfermann S, Wemakor E, Hauk C, *et al.* Quality of medicines in southern Togo: Investigation of antibiotics and of medicines for non-communicable diseases from pharmacies and informal vendors. *PLoS One* 2018;**13**:e0207911. doi:10.1371/journal.pone.0207911

94 Schiavetti B, Wynendaele E, De Spiegeleer B, *et al.* The quality of medicines used in children and supplied by private pharmaceutical wholesalers in Kinshasa, Democratic Republic of Congo: A prospective survey. *Am J Trop Med Hyg* 2018;**98**:894–903. doi:10.4269/ajtmh.17-0732

95 Tshilombo NK, Hamuli PC, Mbinze JK, *et al.* Investigation of the quality of antibiotics-based amoxicillin for monitoring of some different medicine markets of Democratic Republic of Congo. *Am J Anal Chem* 2018;**9**:366–85. doi:10.4236/ajac.2018.98029

96 Lawal MG, Mukhtar MD, Magashi AM. Quality assessment of antibiotic oral drug formulations marketed in Katsina State, Nigeria. *Asian J Pharm Res Dev* 2019;**7**:6–10. doi:10.22270/ajprd.v7i6.615

97 Myers NM, Maina MW, Were PM, *et al.* Lab on paper: assay of beta-lactam pharmaceuticals by redox titration. *Anal Methods* 2019;**11**:4741–50. doi:10.1039/C9AY01547G

98 Scrimgeour S, Nunan M, Sanburg ALC, *et al.* Pharmaceutical quality of antibiotics in Small Island Nations in the Western Pacific region: a pilot survey. *J Pharm Pract Res* 2019;**49**:426–32. doi:10.1002/jppr.1540

99 Tabernero P, Swamidoss I, Mayxay M, *et al.* A random survey of the prevalence of falsified and substandard antibiotics in the Lao PDR. *J Antimicrob Chemother* 2019;**74**:2417–25. doi:10.1093/jac/dkz164

100 Bekoe SO, Ahiabu M-A, Orman E, *et al.* Exposure of consumers to substandard antibiotics from selected authorised and unauthorised medicine sales outlets in Ghana. *Trop Med Int Heal* 2020;**25**:962–75. doi:10.1111/tmi.13442

101 Hand RM, Senarathna SMDKG, Page-Sharp M, *et al.* Quality of benzathine penicillin G: A multinational cross-sectional study. *Pharmacol Res Perspect* 2020;**8**:e00668. doi:10.1002/prp2.668

102 Husaini DC, Kishan UJV, Wu C-Y, *et al.* Field-based screening of selected oral antibiotics in Belize. *PLoS One* 2020;**15**:e0234814. doi:10.1371/journal.pone.0234814

103 Jean-Baptiste T, Carpenter JF, Dahl K, *et al.* Substandard quality of the antimicrobials sold in the street markets in Haiti. *Antibiotics* 2020;**9**:407. doi:10.3390/antibiotics9070407

104 Khurelbat D, Dorj G, Sunderland B, *et al.* A cross-sectional analysis of falsified, counterfeit and substandard medicines in a low-middle income country. *BMC Public Health* 2020;**20**:1–9.

105 Koech LC, Irungu BN, Ng'ang'a MM, *et al.* Quality and brands of amoxicillin formulations in Nairobi, Kenya. *Biomed Res Int* Published Online First: 2020. doi:10.1155/2020/7091278

106 Sakuda M, Yoshida N, Takaoka T, *et al.* Substandard and falsified medicines in Myanmar. *Pharmacy* 2020;**8**. doi:10.3390/pharmacy8010045

107 Schäfermann S, Hauk C, Wemakor E, *et al.* Substandard and falsified antibiotics and medicines against noncommunicable diseases in Western Cameroon and Northeastern Democratic Republic of Congo. *Am J Trop Med Hyg* 2020;**103**:894–908. doi:10.4269/ajtmh.20-0184
