# Supplementary material for: Substandard and falsified antibiotics: neglected drivers of antimicrobial resistance?
Source: BMJ Glob Health. 2022 Aug 18;7(8):e008587. doi: 10.1136/bmjgh-2022-008587 (PMC9394205; doi:10.1136/bmjgh-2022-008587)
Supplement: Supplementary data [file bmjgh-2022-008587supp007.pdf]

Substandard and falsified antibiotics: neglected drivers of antimicrobial resistance?

Supplementary file 7. Failure frequency of antibiotics by country, region and AWaRe group in prevalence surveys included in the review*.

Country/Region	Income	AWARE group	AMC**[1]	Data points	Failed samples	Total samples	% FF
**Africa**				**267**	**1,090**	**3,836**	**28.4**
Burkina Faso	LIC	*ACCESS*	10.4	3	2	7	28.6
		WATCH	3.3	1	0	3	0.0
Cameroon	LMIC	*ACCESS*	Not available	24	136	436	31.2
		WATCH	Not available	1	2	25	8.0
Chad	LIC	*ACCESS*	Not available	7	3	19	15.8
		WATCH	Not available	n/a	n/a	n/a	n/a
Côte d'Ivoire	LMIC	*ACCESS*	8.8	4	33	65	50.8
		WATCH	1.7	n/a	n/a	n/a	n/a
Democratic Republic of the Congo	LIC	*ACCESS*	Not available	13	96	495	19.4
		WATCH	Not available	2	6	43	14.0
Ethiopia	LIC	*ACCESS*	Not available	2	0	2	0.0
		WATCH	Not available	n/a	n/a	n/a	n/a
Ghana	LMIC	*ACCESS*	Not available	19	202	332	60.8
		WATCH	Not available	8	88	119	74.0
Kenya	LMIC	*ACCESS*	Not available	16	83	430	19.3
		WATCH	Not available	2	1	6	16.7
Madagascar	LIC	*ACCESS*	Not available	10	14	59	23.7
		WATCH	Not available	1	0	3	0.0
Malawi	LIC	*ACCESS*	Not available	6	8	70	11.4
		WATCH	Not available	2	0	6	0.0
Mali	LIC	*ACCESS*	Not available	2	2	20	10.0
		WATCH	Not available	1	4	10	40.0
Niger	LIC	*ACCESS*	Not available	8	14	32	43.8
		WATCH	Not available	1	0	1	0.0

Nigeria	LMIC	*ACCESS*	Not available	37	252	544	46.3
		WATCH	Not available	6	46	88	52.3
Rwanda	LIC	*ACCESS*	Not available	3	1	8	12.5
		WATCH	Not available	n/a	n/a	n/a	n/a
Senegal	LMIC	*ACCESS*	Not available	7	31	69	44.9
		WATCH	Not available	2	6	12	50.0
Sierra Leone	LIC	*ACCESS*	Not available	1	0	9	0.0
		WATCH	Not available	n/a	n/a	n/a	n/a
South Africa	UMIC	*ACCESS*	Not available	7	25	186	13.4
		WATCH	Not available	n/a	n/a	n/a	n/a
Sudan	LIC	*ACCESS*	23.1	2	0	6	0.0
		WATCH	6.1	2	1	5	20.0
Tanzania	LMIC	*ACCESS*	15.3	12	10	182	5.5
		WATCH	6.1	2	0	11	0.0
Togo	LIC	*ACCESS*	Not available	21	3	51	5.9
		WATCH	Not available	4	3	13	23.1
Uganda	LIC	*ACCESS*	Not available	6	2	12	16.7
		WATCH	Not available	1	0	3	0.0
Zimbabwe	LMIC	*ACCESS*	Not available	19	16	450	3.6
		WATCH		2	0	4	0.0
**Americas**				**31**	**112**	**898**	**12.5**
Argentina	UMIC	*ACCESS*	Not available	n/a	n/a	n/a	n/a
		WATCH	Not available	1	2	75	2.7
Belize	LMIC	*ACCESS*	Not available	2	7	12	58.3
		WATCH	Not available	1	5	5	100.0
Bolivia	LMIC	*ACCESS*	15.1	n/a	n/a	n/a	n/a
		WATCH	4.4	1	3	34	8.8
Brazil	UMIC	*ACCESS*	14.7	n/a	n/a	n/a	n/a
		WATCH	6.7	1	6	157	3.8
Ecuador	UMIC	*ACCESS*	Not available	n/a	n/a	n/a	n/a
		WATCH	Not available	1	2	43	4.7
Guatemala	UMIC	*ACCESS*	Not available	n/a	n/a	n/a	n/a

		WATCH	Not available	1	4	52	7.7
Haiti	LMIC	*ACCESS*	Not available	6	28	153	18.3
		WATCH	Not available	4	17	27	63.0
Honduras	LMIC	*ACCESS*	Not available	n/a	n/a	n/a	n/a
		WATCH	Not available	1	3	45	6.7
Mexico	UMIC	*ACCESS*	Not available	4	6	9	66.7
		WATCH	Not available	1	0	2	0.0
Paraguay	UMIC	*ACCESS*	8.7	n/a	n/a	n/a	n/a
		WATCH	10.3	1	14	111	12.6
Peru	UMIC	*ACCESS*	7.2	n/a	n/a	n/a	n/a
		WATCH	3.0	1	4	56	7.1
United States	HIC	*ACCESS*	Not available	3	1	3	33.3
		WATCH	Not available	n/a	n/a	n/a	n/a
Uruguay	HIC	*ACCESS*	Not available	n/a	n/a	n/a	n/a
		WATCH	Not available	1	4	78	5.1
Venezuela	N/A	*ACCESS*	Not available	n/a	n/a	n/a	n/a
		WATCH	Not available	1	6	36	16.7
**Asia**				**185**	**932**	**6,202**	**15.0**
Afghanistan	LIC	*ACCESS*	Not available	2	2	74	2.7
		WATCH	Not available	2	3	51	5.9
Armenia	UMIC	*ACCESS*	Not available	n/a	n/a	n/a	n/a
		WATCH	Not available	2	1	24	4.2
Azerbaijan	UMIC	*ACCESS*	Not available	n/a	n/a	n/a	n/a
		WATCH	Not available	2	0	10	0.0
Bangladesh	LMIC	*ACCESS*	Not available	5	31	69	44.9
		WATCH	Not available	3	17	20	85.0
Cambodia	LMIC	*ACCESS*	Not available	19	125	697	17.9
		WATCH	Not available	9	106	502	21.1
		Non-AWARE	Not available	1	6	9	66.7
China	UMIC	*ACCESS*	Not available	1	0	3	0.0
		WATCH	Not available	n/a	n/a	n/a	n/a
India	LMIC	*ACCESS*	Not available	11	17	380	4.5

		WATCH	Not available	16	69	675	10.2
Indonesia	LMIC	*ACCESS*	Not available	4	19	85	22.4
		WATCH	Not available	1	0	19	0.0
Kazakhstan	UMIC	*ACCESS*	Not available	1	0	3	0.0
		WATCH	Not available	6	4	32	12.5
Lao People's Democratic Republic	LMIC	*ACCESS*	Not available	22	289	1242	23.3
		WATCH	Not available	3	8	92	8.7
Mongolia	LMIC	*ACCESS*	46.2	12	85	935	9.1
		WATCH	18.1	1	17	118	14.4
Myanmar	LMIC	*ACCESS*	Not available	11	28	193	14.5
		WATCH	Not available	7	57	202	28.2
Nepal	LMIC	*ACCESS*	Not available	5	2	8	25.0
		WATCH	Not available	1	0	3	0.0
Pakistan	LMIC	*ACCESS*	Not available	n/a	n/a	n/a	n/a
		WATCH	Not available	4	17	108	15.7
Saudi Arabia	HIC	*ACCESS*	Not available	1	9	83	10.8
		WATCH	Not available	n/a	n/a	n/a	n/a
Tajikistan	LMIC	*ACCESS*	Not available	3	2	9	22.2
		WATCH	Not available	1	0	3	0.0
Thailand	UMIC	*ACCESS*	Not available	8	3	331	0.9
		WATCH	Not available	n/a	n/a	n/a	n/a
Uzbekistan	LMIC	*ACCESS*	Not available	n/a	n/a	n/a	n/a
		WATCH	Not available	2	0	15	0.0
Viet Nam	LMIC	*ACCESS*	Not available	10	12	177	6.8
		WATCH	Not available	4	0	6	0.0
Unknown***		*ACCESS*	Not available	5	12	32	43.8
		WATCH	Not available	n/a	n/a	n/a	n/a
**Europe**				**9**	**5**	**65**	**7.7**
Belarus	UMIC	*ACCESS*	9.8	n/a	n/a	n/a	n/a
		WATCH	6.3	2	3	24	12.5
Estonia	HIC	*ACCESS*	6.7	1	0	4	0.0
		WATCH	3.6	n/a	n/a	n/a	n/a

Germany	HIC	*ACCESS*	5.7	1	0	1	0.0
		WATCH	3.0	n/a	n/a	n/a	n/a
Russian Federation	UMIC	*ACCESS*	7.1	1	2	4	50.0
		WATCH	6.5	n/a	n/a	n/a	n/a
Ukraine	LMIC	*ACCESS*	Not available	n/a	n/a	n/a	n/a
		WATCH	Not available	2	0	23	0.0
United Kingdom	HIC	*ACCESS*	13.2	2	0	9	0.0
		WATCH	4.2	n/a	n/a	n/a	n/a
**Oceania**				14	15	100	15.0
Papua New Guinea	LMIC	*ACCESS*	Not available	3	9	67	13.4
		WATCH	Not available	n/a	n/a	n/a	n/a
Unknown****		*ACCESS*	Not available	10	5	30	16.7
		WATCH	Not available	1	0	3	0.0
**Unknown*******		*ACCESS*		12	6	257	2.3
		WATCH		15	189	2188	8.6
		Non-AWARE		1	0	1	0.0

HIC: High income, LIC: Low income, UMIC: Upper middle income, LMIC: Lower middle

* Due to the limited number of samples tested for quality in the studies included in this review, the figures should not be interpreted as representative of the prevalence of specific SF antibiotics (please refer to the discussion section of the article for more details).

** Median Antimicrobial Consumption (AMC) between 2016-2018, in Defined Daily Doses per 1,000 inhabitants per day (DID).

*** Multicountry study (Egypt, Jordan, Lebanon, Saudi Arabia) with no breakdown of the results by country

**** Multicountry study (Papua New Guinea, Solomon Islands, Vanuatu) with no breakdown of the results by country

***** Aggregated data without further details of continent and/or countries

Source of AMD data = World Health Organization. WHO report on surveillance of antibiotic consumption: 2016-2018 early implementation. 2018;:1–127.https://www.who.int/medicines/areas/rational_use/oms-amr-amc-report-2016-2018/en/ (accessed 8 Sep 2019).

BMJ Publishing Group Limited (BMJ) disclaims all liability and responsibility arising from any reliance placed on this supplemental material which has been supplied by the author(s)
