# Supplementary material for: Substandard and falsified antibiotics: neglected drivers of antimicrobial resistance?
Source: BMJ Glob Health. 2022 Aug 18;7(8):e008587. doi: 10.1136/bmjgh-2022-008587 (PMC9394205; doi:10.1136/bmjgh-2022-008587)
Supplement: Supplementary data [file bmjgh-2022-008587supp008.pdf]

Substandard and falsified antibiotics: neglected drivers of antimicrobial resistance?

Supplementary file 8. Antibiotic quality failure frequency by WHO AWaRe groups.

WHO group/API	DID *[1]	Median FF (%)	IQR FF (%)	No. samples	No. SF	Median N° samples per data point	IQR N° Samples per data point	Data points	Countries
**ACCESS**	5.6-12.4	10	0.0-40.0	8,354	1,633	8	3-25	394	
Amikacin		0	N/A	17	0	17	N/A	1	India
Amoxicillin		3	0.0-8.3	2,208	355	25	5-52	91	Afghanistan, Bangladesh, Belize, Cambodia, Cameroon, Chad, Côte d'Ivoire, Democratic Republic of the Congo, Ghana, Haiti, India, Indonesia, Kenya, Lao People's Democratic Republic, Madagascar, Malawi, Mexico, Mongolia, Myanmar, Nepal, Niger, Nigeria, Papua New Guinea, Rwanda, Saudi Arabia, Senegal, Sierra Leone, South Africa, Sudan, Tajikistan, Tanzania, Thailand, Togo, Uganda, United Kingdom, United States, Unknown, Viet Nam, Zimbabwe
Amoxicillin-Clavulanate		21.3	5.8-38.6	437	130	13	5-42	21	Cambodia, Cameroon, Democratic Republic of the Congo, Germany, Ghana, Haiti, India, Kazakhstan, Kenya, Malawi, Nigeria, South Africa, Togo, Unknown

Ampicillin	5	2.0-9.0	1,010	211	19	9-45	44	Burkina Faso, Cameroon, Chad, Côte d'Ivoire, Democratic Republic of the Congo, Ghana, Kenya, Lao People's Democratic Republic, Madagascar, Mexico, Mongolia, Myanmar, Nepal, Niger, Nigeria, Senegal, Tajikistan, Tanzania, Uganda, Unknown, Viet Nam, Zimbabwe
Ampicillin - Cloxacillin	6.5	1.3-19.3	110	62	12	8-32	8	India, Nigeria, Unknown
Cefadroxil	0	N/A	14	0	14	N/A	1	India
Cefalexin	0	N/A	21	0	10	N/A	2	India, Unknown
Cephadrine	100	N/A	6	6	6	N/A	1	Bangladesh
Chloramphenicol	1.5	0.0-5.8	132	32	13	4-24	11	Cambodia, Cameroon, Chad, Indonesia, Madagascar, Malawi, Myanmar, Niger, Nigeria, Unknown, Viet Nam
Clindamycin	83.3	N/A	6	5	6	N/A	2	Ghana, Nigeria
Cloxacillin	2.5	0.5-3.0	54	13	7	4-9	10	Ghana, Haiti, Lao People's Democratic Republic, Nigeria, Unknown
Doxycycline	1	0.0-8.5	347	73	12	9-35	18	Cameroon, Democratic Republic of the Congo, Estonia, Lao People's Democratic Republic, Mongolia, Myanmar, Nigeria, Papua New Guinea, Russian Federation, Togo, Unknown, Zimbabwe
Flucloxacillin	100	90.6-100.0	21	18	4	3-10	3	Ghana, Unknown

Gentamicin	1	0.0-3.0	226	27	22	10-35	21	Afghanistan, Burkina Faso, Cambodia, Ghana, India, Kenya, Madagascar, Mali, Myanmar, Nepal, Niger, Nigeria, Tajikistan, Tanzania, Uganda, Viet Nam, Zimbabwe
Metronidazole	3.5	0.3-7.8	838	166	21	10-42	38	Bangladesh, Cambodia, Cameroon, Chad, Côte d'Ivoire, Democratic Republic of the Congo, Ghana, Haiti, Kenya, Madagascar, Mongolia, Myanmar, Niger, Nigeria, Rwanda, Sudan, Tanzania, Togo, Unknown, Viet Nam
Oxacillin	0	N/A	2	0	2	N/A	1	Niger
Penicillin G (IV/IM)	0	0.0-3.0	209	17	6	5-28	13	Burkina Faso, Ghana, Madagascar, Myanmar, Nigeria, Tanzania, Uganda, Unknown, Zimbabwe
Penicillin V (PO)	0	0.0-1.0	230	43	8	3-41	12	Cambodia, Cameroon, Democratic Republic of the Congo, Ghana, Malawi, Senegal, Togo, Unknown, Zimbabwe
Penicillin-unspecified formulation	1	0.5-1.5	20	2	10	8-12	4	Cameroon, Chad, Madagascar, Senegal

Co-trimoxazole		5	2.0-12.0	1,255	329	20	6-44	62	Belize, Cambodia, Cameroon, Chad, China, Côte d'Ivoire, Democratic Republic of the Congo, Ethiopia, Ghana, Haiti, India, Indonesia, Kenya, Lao People's Democratic Republic, Madagascar, Malawi, Mexico, Mongolia, Myanmar, Niger, Nigeria, Rwanda, Senegal, South Africa, Tanzania, Thailand, Togo, United Kingdom, United States, Unknown
Tetracycline		1	0.0-6..0	1,191	144	22	11-101	30	Cambodia, Cameroon, Chad, Ghana, Haiti, Indonesia, Kenya, Lao People's Democratic Republic, Madagascar, Mali, Myanmar, Niger, Nigeria, Senegal, Thailand, Unknown, Viet Nam, Zimbabwe
**WATCH**	1.3-6.1	8.2	0.0-30.0	5,191	718	12	3-43	138	
Azithromycin		3.5	2.5-7.8	61	27	16	6-25	5	Bangladesh, Ghana, Haiti, Nigeria, Unknown
Cefixime		6	3.0-6.5	104	13	37	22-49	3	Bangladesh, Cambodia, Unknown
Cefotaxime		0	N/A	21	0	21	N/A	1	India
Ceftazidime		0	N/A	7	0	7	N/A	1	India

Ceftriaxone	2	0.0-6.5	309	49	26	3-42	24	Afghanistan, Burkina Faso, Cambodia, Ghana, Kenya, Lao People's Democratic Republic, Madagascar, Myanmar, Nepal, Nigeria, Pakistan, Sudan, Tajikistan, Tanzania, Uganda, Unknown, Viet Nam, Zimbabwe
Cefuroxime	2.5	0.0-13.5	188	71	11	6-44	8	Cambodia, Ghana, India, Malawi, Myanmar, Unknown
Ciprofloxacin	3	0.0-8.0	3,511	366	19	8-57	47	Afghanistan, Argentina, Bangladesh, Belize, Bolivia, Brazil, Cambodia, Cameroon, Democratic Republic of the Congo, Ecuador, Ghana, Guatemala, Haiti, Honduras, India, Indonesia, Lao People's Democratic Republic, Malawi, Mexico, Mongolia, Myanmar, Nigeria, Pakistan, Paraguay, Peru, Senegal, Sudan, Tanzania, Togo, Unknown, Uruguay, Venezuela
Clarithromycin	6	0.8-11.8	102	26	26	3-49	4	Cambodia, Haiti, Unknown
Erythromycin	3.5	0.8-9.8	345	66	12	4-23	14	Ghana, Haiti, India, Kenya, Mali, Myanmar, Nigeria, Senegal, Unknown
Kanamycin	0.5	0.3-0.8	70	1	35	19-51	7	Armenia, Azerbaijan, Belarus, Kazakhstan, Ukraine, Uzbekistan
Levofloxacin	5	0.3-12.8	185	42	26	6-56	6	Cambodia, India, Kazakhstan, Myanmar, Pakistan, Unknown
Moxifloxacin	0	0.0-0.0	5	0	3	2-3	2	Kazakhstan, Unknown

Norfloxacin		0	N/A	1	0	1	N/A	1	Niger
Ofloxacin		1	0.0-6.0	192	26	7	3-55	12	Armenia, Azerbaijan, Belarus, Cambodia, India, Kazakhstan, Lao People's Democratic Republic, Pakistan, Ukraine, Unknown, Uzbekistan
Roxithromycin		10.5	7.3-13.8	71	21	36	25-46	2	Cambodia, India
Streptomycin		52.7	N/A	19	10	19	N/A	1	Nigeria
**RESERVE**	Not available								
No data									
**Antibiotics not in AWaRe**	Not available	33.3	16.7-50.0	10	6	5	3-7	2	
Nalidixic acid		66.7	N/A	9	6	9	N/A	1	Cambodia
Sulfamethoxazole (without trimethoprim)		0	N/A	1	0	1	N/A	1	Unknown (Africa and Asia)

*Median Antimicrobial Consumption (AMC) in 2015 in LMIC and HIC, respectively, as Defined Daily Doses per 1,000 inhabitants per day (DID).

1 Klein EY, Milkowska-Shibata M, Tseng KK, *et al.* Assessment of WHO antibiotic consumption and access targets in 76 countries, 2000–15: an analysis of pharmaceutical sales data. *Lancet Infect Dis* 2021;**21**:107–15. doi:10.1016/S1473-3099(20)30332-7
