# Supplementary material for: Substandard and falsified antibiotics: neglected drivers of antimicrobial resistance?
Source: BMJ Glob Health. 2022 Aug 18;7(8):e008587. doi: 10.1136/bmjgh-2022-008587 (PMC9394205; doi:10.1136/bmjgh-2022-008587)
Supplement: Supplementary data [file bmjgh-2022-008587supp009.pdf]

Substandard and falsified antibiotics: neglected drivers of antimicrobial resistance?

Supplementary file 9. Failure frequency per type of quality test by API in prevalence surveys of antibiotic quality*.

Colour gradient: grey (no data), orange (<20% FF), light red (21-40% FF), red (41-70% FF), brown (>70% FF).

Type of defect % (n/N)

API name	API Content	API ID & Semi-quantitation	Dissolution	Impurities/ contaminant/related substance	Uniformity of units	Packaging/ label/physical appearance inspection	Other physical analysis***	Other chemical analysis****
**Total**	16.5 (1,701/10,307)	7.5 (210/2,783)	9.1 (296/3,261)	3.5 (12/346)	6.5 (255/3,916)	2.8 (129/4,612)	2.2 (71/3,290)	4.4 (187/4,212)
**Amikacin**	0 (0/17)	n/a	n/a	n/a	n/a	n/a	n/a	n/a
**Amoxicillin**	14.7 (268/1,829)	8.3 (3/36)	6.7 (31/461)	17.7 (3/17)	2.8 (16/581)	2.1 (13/609)	0.2 (1/541)	0.8 (5/599)
**Amoxicillin-Clavulanic acid****	26.6 (98/369)	0 (0/22)	14.3 (62/432)	n/a	4.5 (13/292)	8.4 (12/143)	0 (0/82)	4.8 (4/84)
**Ampicillin**	12.0 (114/951)	4.2 (1/24)	0 (0/147)	30.8 (8/26)	9.6 (50/522)	23.3 (27/116)	0.5 (1/198)	9.4 (43/459)
**Ampicillin-Cloxacillin****	41.7 (80/192)	0 (0/2)	n/a	n/a	n/a	n/a	0 (0/2)	0 (0/2)
**Azithromycin**	65.6 (21/32)	0 (0/25)	0 (0/20)	n/a	n/a	0 (0/25)	0 (0/25)	10.7 (3/28)
**Benzylpenicillin (penicillin G)**	13.1 (14/107)	n/a	n/a	0 (0/11)	n/a	0 (0/3)	0 (0/31)	0 (0/1)
**Cefadroxil**	0 (0/14)	n/a	n/a	n/a	n/a	n/a	n/a	n/a
**Cefalexin**	0 (0/10)	0 (0/11)	n/a	n/a	n/a	n/a	0 (0/11)	0 (0/11)
**Cefixime**	15.2 (10/66)	0 (0/37)	5.6 (3/54)	n/a	0 (0/55)	n/a	0 (0/37)	0 (0/37)
**Cefotaxime**	0 (0/21)	n/a	n/a	n/a	n/a	n/a	n/a	n/a

**Ceftazidime**	0 (0/7)	n/a	n/a	n/a	n/a	n/a	n/a	n/a
**Ceftriaxone**	16.5 (47/284)	0 (0/26)	n/a	0 (0/35)	12.4 (18/145)	0 (0/38)	n/a	0.7 (1/149)
**Cefuroxime**	28.3 (51/180)	0 (0/8)	8.7 (10/115)	n/a	26.6 (30/113)	n/a	0 (0/8)	8.3 (1/12)
**Cephradine**	100 (6/6)	n/a	n/a	n/a	n/a	n/a	n/a	n/a
**Chloramphenicol**	31.7 (40/126)	18.2 (2/11)	14.3 (1/7)	n/a	0 (0/27)	0 (0/1)	5.3 (2/38)	12.1 (4/33)
**Ciprofloxacin**	14.8 (116/783)	8.3 (152/1,836)	1.1 (3/276)	n/a	5.5 (12/220)	1.0 (18/1,808)	2.4 (13/538)	3.2 (10/311)
**Clarithromycin**	5.1 (5/98)	0 (0/3)	28.4 (25/88)	n/a	8.6 (8/93)	n/a	0 (0/3)	25.0 (1/4)
**Clindamycin**	66.7 (4/6)	n/a	16.7 (1/6)	n/a	n/a	0 (0/6)	n/a	n/a
**Cloxacillin**	40 (10/25)	0 (0/26)	0 (0/5)	n/a	0 (0/7)	28.6 (2/7)	0 (0/31)	8.8 (3/34)
**Doxycycline**	20.1 (68/339)	0 (0/10)	1.8 (4/217)	0 (0/8)	3.1 (8/255)	0 (0/182)	1.3 (2/157)	0.6 (1/180)
**Erythromycin**	48.3 (28/58)	8.4 (13/154)	69.2 (9/13)	n/a	0 (0/5)	5.0 (1/20)	5.8 (9/154)	18.6 (8/43)
**Flucloxacillin**	85.7 (18/21)	n/a	n/a	n/a	n/a	0 (0/1)	n/a	0 (0/4)
**Gentamicin**	9.8 (22225)	n/a	0 (0/1)	n/a	0 (0/1)	0 (0/30)	0 (0/1)	1.9 (3/157)
**Kanamycin**	0 (0/67)	33.3 (1/3)	n/a	n/a	n/a	0 (0/67)	n/a	0 (0/67)
**Levofloxacin**	14.7 (26/177)	0 (0/12)	29.7 (19/64)	n/a	n/a	0 (0/2)	6.3 (1/16)	0 (0/10)
**Metronidazole**	19.7 (146/741)	0 (0/40)	5.5 (15/274)	n/a	8.4 (36/430)	1.2 (5/416)	3.0 (11/363)	0.6 (2/351)
**Moxifloxacin**	n/a	0 (0/5)	n/a	n/a	n/a	0 (0/2)	0 (0/5)	0 (0/3)
**Nalidixic acid**	0 (0/9)	n/a	66.7 (6/9)	n/a	0 (0/9)	n/a	n/a	n/a
**Norfloxacin**	0 (0/1)	n/a	0 (0/1)	n/a	0 (0/1)	n/a	0 (0/1)	0 (0/1)
**Ofloxacin**	10.7 (20/187)	0 (0/8)	14.9 (15/101)	1.9 (1/53)	18.0 (16/89)	0 (0/54)	8.3 (1/12)	0 (0/20)
**Oxacillin**	0 (0/2)	n/a	0 (0/2)	n/a	0 (0/2)	n/a	0 (0/2)	0 (0/2)
**Penicillin - unspecified formulation**	7.1 (1/14)	0 (0/6)	n/a	n/a	7.1 (1/14)	0 (0/6)	5.0 (1/20)	0 (0/14)

**Phenoxymethylpenicillin (penicillin V)**	19.8 (43/217)	0 (0/11)	1.0 (1/103)	n/a	0 (0/3)	2.4 (1/42)	0 (0/45)	0 (0/44)
**Procaine Benzylpenicillin (penicillin G) - intramuscular**	2.9 (3/102)	n/a	n/a	0 (0/5)	0 (0/5)	0 (0/5)	n/a	0 (0/10)
**Roxithromycin**	5.6 (4/71)	n/a	29.8 (17/57)	n/a	0 (0/57)	n/a	n/a	n/a
**Streptomycin**	52.6 (10/19)	n/a	n/a	n/a	n/a	n/a	n/a	n/a
**Sulfamethoxazole-Trimethoprim****	19.9 (397/2,093)	4.8 (4/84)	12.9 (72/558)	0 (0/180)	0.5 (4/774)	1.6 (10/647)	2.4 (19/781)	5.8 (75/1,286)
**Sulfamethoxazole-Uncombined**	n/a	0 (0/1)	n/a	n/a	n/a	n/a	0 (0/1)	0 (0/1)
**Tetracycline**	3.4 (29/842)	6.3 (24/382)	0.8 (2/250)	0 (0/11)	19.9 (43/216)	10.5 (40/382)	5.4 (10/187)	9.1 (22/242)

* Samples are often subjected to more than one test technique within each category. Numbers represent testing events per API sample

** Combination antibiotics were usually tested twice for API content, once for each ingredient. However, some authors analysed only one of the APIs or an uneven number of them

*** Includes disintegration, friability, hardness, thickness, wetting time and water absorption testing

**** Includes API identification, degradation products, pH, and other undeclared chemical tests
