# Supplementary material for: Substandard and falsified antibiotics: neglected drivers of antimicrobial resistance?
Source: BMJ Glob Health. 2022 Aug 18;7(8):e008587. doi: 10.1136/bmjgh-2022-008587 (PMC9394205; doi:10.1136/bmjgh-2022-008587)
Supplement: Supplementary data [file bmjgh-2022-008587supp010.pdf]

Substandard and falsified antibiotics: neglected drivers of antimicrobial resistance?

Supplementary file 10. Concordance with the 26 items of the MEDQUARG checklist of the prevalence surveys of antibiotics quality.

Item	Description	Iwuagwu, 1992	Okeke et al, 1995	Gimenez et al, 1997	Shakoor et al, 1997	Kibwage et al, 1998	Stenson et al, 1998	Wondemagegnehu (WHO), 1999	Kamau et al, 2001
1	Identify as MQ paper with abstract of what was done and found, describing survey and analytical methods	N	Y	N	Y	N	Y	Y	N
2	Background and objectives	Y	Y	Y	Y	Y	Y	Y	Y
3	Survey details (when and where collected & analysed)	N	N	N	N	N	N	N	N
4	Definitions (Substandard, falsified...)	N	N	Y	Y	N	Y	Y	N
5	Outlets and outlet size indicator (sales, turnover, etc.)	N	N	N	N	N	N	N	N
6	Sampling design, size calculation, definition of sample	N	N	N	N	N	N	N	N
7	Samplers and method of randomization	N	N	N	Y	N	N	Y	N
8	Statistical methods	N	N	N	N	N	N	N	N
9	Ethical issues	N	N	N	N	N	N	N	N
10	Packaging and reference standard	N	N	N	N	N	N	Y	N
11	Chemical analysis SOPs, location of laboratory and reference standards	Y	Y	N	N	N	Y	Y	Y
12	Method validation against reference or study	N	N	N	Y	Y	Y	Y	N
13	Blinding between chemistry analysis and packaging	N	N	N	N	N	N	N	N
14	Outlets: details of outlets actually sampled	N	N	Y	Y	N	N	N	N
15	Missing samples (ie why an outlet did not procure samples, lost, etc.)	N	N	N	N	N	N	N	N
16	Packaging & chemistry results and correlation. Include details of products sampled	N	N	N	N	N	N	N	N
17	Category of poor-quality medicine (stating if SF in results)	N	N	N	N	N	N	Y	N
18	State company and address as given on packaging	N	N	N	N	Y	N	N	Y
19	Sharing data with MRA	N	N	Y	N	N	Y	Y	N
20	Dissemination: packaging features that allows ID of falsified?	N	N	N	N	N	N	N	N
21	Key results in relation to objectives	Y	Y	N	Y	Y	Y	Y	Y
22	Limitations	N	N	Y	N	N	N	N	N
23	Interpretation in conjunction with prior studies and in relation to public health	N	Y	N	Y	N	Y	N	N
24	Intervention	Y	Y	N	Y	Y	Y	Y	N
25	Conflict of interest	N	N	N	N	N	N	N	N
26	Funding	N	N	Y	N	Y	Y	Y	N

Y= Reported; N=Not reported

Item	Taylor et al, 2001	Kolawole et al, 2002	Sow et al, 2002	Prazuck et al, 2002	Ahmed et al, 2003	Kamau et al, 2003	Risha, 2003	Basco, 2004	Syhakhang et al, 2004	Weir et al, 2005	Lon et al, 2006	Obodozie et al, 2006
**1**	Y	N	Y	Y	N	N	Y	Y	Y	Y	Y	N
**2**	Y	Y	N	Y	N	Y	Y	Y	Y	Y	Y	Y
**3**	N	N	N	N	N	N	N	N	N	N	N	N
**4**	Y	Y	Y	N	N	N	N	Y	N	N	N	N
**5**	N	N	N	N	N	N	N	N	N	N	N	N
**6**	N	N	N	N	N	N	N	N	N	N	N	N
**7**	Y	N	Y	N	N	N	N	Y	Y	N	Y	N
**8**	N	Y	Y	N	N	N	Y	N	Y	N	Y	N
**9**	N	N	N	N	N	N	N	N	Y	Y	N	N
**10**	N	N	N	N	N	N	N	N	N	N	Y	N
**11**	Y	N	Y	Y	Y	Y	Y	Y	Y	Y	Y	Y
**12**	Y	N	N	N	N	N	Y	Y	Y	Y	Y	Y
**13**	N	N	N	N	N	N	N	N	Y	N	N	N
**14**	Y	N	Y	N	N	N	N	Y	N	N	Y	Y
**15**	N	N	N	N	N	N	N	N	N	N	N	N
**16**	N	N	N	N	N	N	N	N	N	N	Y	N
**17**	N	N	N	N	N	N	N	Y	N	N	Y	N
**18**	N	N	N	Y	N	Y	Y	N	N	N	N	N
**19**	N	N	N	N	N	N	N	N	Y	N	Y	N
**20**	N	N	N	N	N	N	N	N	N	N	Y	N
**21**	Y	Y	Y	Y	Y	Y	Y	Y	Y	Y	Y	Y
**22**	N	N	N	Y	N	N	N	N	Y	Y	Y	Y
**23**	Y	Y	Y	Y	N	N	Y	Y	Y	Y	Y	Y
**24**	N	N	Y	Y	Y	Y	Y	Y	Y	Y	Y	N
**25**	N	N	N	N	N	N	N	N	Y	Y	Y	N
**26**	Y	N	N	N	N	N	Y	Y	Y	N	Y	N

Y= Reported; N=Not reported

Item	Vijaykadga et al, 2006	Meos et al, 2008	Kyriacos et al, 2008	Pouillot et al, 2008	Bate et al, 2009	Obaid, 2009	Zaheer et al, 2009	Hadi et al, 2010	Khan et al, 2010	Yoshida et al, 2010	Bate et al, 2011	Haider et al, 2011	Kamuhabwa et al, 2011
**1**	Y	Y	Y	N	Y	N	N	Y	Y	Y	Y	N	Y
**2**	Y	Y	Y	Y	Y	Y	Y	Y	Y	Y	Y	N	Y
**3**	N	N	N	N	Y	N	N	N	N	Y	N	N	N
**4**	Y	Y	Y	N	N	Y	N	Y	Y	Y	Y	N	N
**5**	N	N	N	N	N	N	N	N	N	N	N	N	N
**6**	N	N	N	N	N	N	N	N	N	Y	N	N	N
**7**	Y	N	N	N	Y	N	N	Y	N	N	Y	N	N
**8**	N	N	N	N	N	N	N	Y	Y	Y	Y	N	N
**9**	N	N	N	N	N	N	N	Y	N	N	N	N	N
**10**	Y	N	N	N	N	N	N	N	Y	Y	Y	N	N
**11**	Y	N	Y	N	Y	Y	Y	Y	Y	Y	Y	N	N
**12**	Y	N	Y	N	Y	N	N	Y	N	Y	Y	N	N
**13**	N	N	N	N	N	N	N	N	N	N	N	N	N
**14**	Y	N	N	N	Y	N	N	Y	Y	Y	Y	N	N
**15**	N	N	N	N	N	N	N	Y	N	N	N	N	N
**16**	N	N	N	N	N	N	N	N	N	N	N	N	N
**17**	Y	N	Y	N	N	N	N	Y	Y	N	N	N	N
**18**	N	Y	N	N	N	N	N	Y	N	N	N	N	N
**19**	Y	N	N	N	N	N	N	N	Y	Y	N	N	N
**20**	N	N	N	N	N	N	N	N	N	Y	N	N	N
**21**	Y	Y	Y	Y	Y	Y	Y	Y	Y	Y	Y	Y	Y
**22**	Y	Y	N	N	N	N	N	Y	Y	Y	N	N	N
**23**	Y	N	Y	Y	Y	Y	N	Y	Y	Y	Y	N	Y
**24**	Y	N	Y	Y	Y	Y	N	Y	Y	Y	Y	Y	Y
**25**	N	N	N	N	Y	N	N	Y	N	Y	N	N	N
**26**	Y	Y	N	N	Y	N	N	Y	Y	Y	Y	N	N

Y= Reported; N=Not reported

Item	Nair et al, 2011	Karlage et al, 2012	Akinkunmi, 2013	Egbo, 2013	Khan et al, 2013	Phanouvong et al, 2013a	Phanouvong et al, 2013b	Ramachandran et al, 2013	Hetzel, 2014	Khurelbat et al, 2014	Khuluza, 2014
**1**	Y	Y	N	Y	Y	Y	Y	Y	Y	N	Y
**2**	Y	Y	Y	Y	N	Y	Y	Y	Y	Y	Y
**3**	N	N	N	N	N	N	N	N	N	N	N
**4**	Y	Y	N	Y	Y	Y	Y	N	Y	Y	Y
**5**	N	N	N	N	N	N	N	N	N	N	N
**6**	N	N	N	N	N	Y	Y	Y	N	N	N
**7**	Y	N	N	N	Y	Y	Y	N	Y	Y	N
**8**	N	N	N	Y	Y	N	Y	Y	Y	Y	Y
**9**	N	N	N	N	Y	N	N	N	Y	Y	N
**10**	Y	N	N	Y	Y	Y	Y	N	N	Y	N
**11**	N	Y	N	Y	Y	Y	Y	Y	Y	Y	Y
**12**	Y	Y	N	Y	N	Y	Y	Y	Y	Y	Y
**13**	N	N	N	N	N	N	N	Y	Y	N	N
**14**	Y	N	Y	N	Y	Y	Y	N	Y	Y	Y
**15**	N	N	N	N	N	N	N	N	N	N	N
**16**	Y	N	N	Y	Y	N	N	N	N	N	N
**17**	Y	N	N	N	Y	N	N	N	N	Y	N
**18**	Y	Y	N	Y	N	N	N	N	Y	N	N
**19**	N	N	N	N	Y	N	Y	N	N	Y	N
**20**	Y	N	Y	Y	N	N	N	N	Y	N	N
**21**	Y	Y	Y	Y	Y	Y	N	Y	Y	Y	Y
**22**	Y	Y	Y	N	Y	Y	Y	N	Y	Y	N
**23**	Y	N	Y	N	Y	Y	N	Y	Y	Y	Y
**24**	Y	N	Y	Y	Y	Y	Y	Y	Y	Y	Y
**25**	N	Y	Y	N	Y	N	N	N	Y	Y	N
**26**	N	N	Y	Y	Y	Y	Y	Y	Y	Y	N

Y= Reported; N=Not reported

Item	Bate et al, 2015	Boadu et al, 2015	Fadeyi, 2015	Tshilumba et al, 2015	Wang et al, 2015	Khan et al, 2016	Kaale et al, 2016	Mwamba et al, 2016	Nga et al, 2016	Osei-Safo et al, 2016	Islam, 2017	Islam et al, 2017	Khuluza et al, 2017
1	Y	Y	Y	Y	Y	Y	Y	N	Y	N	Y	Y	Y
2	N	N	Y	Y	Y	Y	Y	Y	Y	Y	Y	Y	Y
3	N	N	N	N	N	N	N	N	N	N	N	N	N
4	Y	Y	N	Y	Y	Y	N	N	N	Y	N	N	Y
5	N	N	N	N	N	N	N	N	N	N	N	N	N
6	N	N	N	N	N	N	N	N	N	N	N	N	N
7	Y	N	N	N	N	N	Y	N	N	Y	N	Y	Y
8	N	Y	N	N	N	Y	Y	Y	Y	N	Y	Y	Y
9	N	N	Y	N	N	N	N	N	N	N	N	N	Y
10	Y	N	Y	Y	N	N	N	Y	N	N	Y	Y	Y
11	Y	Y	Y	N	Y	N	N	Y	N	Y	Y	N	Y
12	Y	Y	Y	N	N	Y	N	N	N	Y	Y	N	Y
13	N	N	N	N	N	N	N	N	N	N	N	N	N
14	N	N	N	Y	Y	N	Y	Y	Y	N	Y	Y	Y
15	N	N	N	N	N	N	N	N	N	N	N	N	Y
16	N	N	N	N	N	N	N	N	N	N	N	N	Y
17	Y	N	Y	N	N	N	N	N	N	Y	N	N	Y
18	N	N	Y	N	Y	N	N	N	N	N	N	N	N
19	N	Y	N	N	N	N	Y	Y	N	N	Y	Y	Y
20	Y	N	N	Y	N	N	Y	Y	N	N	Y	N	Y
21	Y	Y	Y	Y	Y	Y	Y	Y	Y	Y	Y	Y	Y
22	Y	N	Y	N	N	N	N	Y	N	N	Y	N	Y
23	Y	Y	Y	Y	N	N	Y	Y	Y	Y	Y	Y	Y
24	Y	Y	Y	Y	N	Y	Y	Y	N	Y	Y	Y	Y
25	N	N	N	Y	Y	Y	Y	Y	Y	N	N	Y	N
26	N	N	Y	N	Y	N	Y	Y	N	N	N	Y	Y

Y= Reported; N=Not reported

Item	Nabirova et al, 2017	Ononna et al, 2017	Petersen et al, 2017	Schiavetti et al, 2018	Bate et al, 2018	Ernest et al, 2018	Frimpong et al, 2018	Islam et al, 2018	Joda et al, 2018	Lehmann et al, 2018a	Lehmann et al, 2018b	Schafermann et al, 2018	Tshilombo et al, 2018
**1**	Y	N	Y	Y	N	Y	Y	Y	Y	Y	Y	Y	N
**2**	Y	Y	Y	Y	Y	Y	Y	N	Y	Y	Y	Y	Y
**3**	Y	N	N	N	N	N	N	N	N	Y	N	N	N
**4**	Y	N	Y	Y	Y	N	Y	Y	Y	Y	Y	Y	Y
**5**	Y	Y	N	Y	N	N	N	N	N	N	N	N	N
**6**	Y	N	N	Y	N	N	N	N	N	N	N	Y	N
**7**	N	N	Y	Y	Y	Y	N	Y	N	Y	N	Y	Y
**8**	Y	N	N	Y	N	N	N	Y	Y	Y	Y	Y	Y
**9**	Y	N	N	Y	N	N	N	Y	N	N	N	N	N
**10**	Y	N	Y	Y	Y	N	N	Y	N	Y	N	N	N
**11**	Y	Y	Y	Y	Y	N	Y	Y	Y	Y	Y	Y	Y
**12**	Y	N	Y	Y	Y	N	N	N	Y	Y	Y	Y	Y
**13**	N	N	N	Y	N	N	N	N	N	Y	N	N	N
**14**	Y	N	N	Y	N	Y	Y	Y	N	Y	Y	Y	N
**15**	Y	N	Y	Y	Y	N	N	N	N	Y	N	Y	N
**16**	Y	N	N	Y	N	N	Y	N	N	Y	N	Y	N
**17**	N	N	Y	Y	N	N	Y	Y	N	N	N	Y	N
**18**	Y	N	Y	N	N	N	N	N	N	N	N	N	N
**19**	Y	N	Y	Y	N	N	N	Y	N	N	Y	N	N
**20**	Y	N	Y	Y	N	N	N	N	N	N	N	N	N
**21**	Y	Y	Y	Y	Y	Y	Y	Y	Y	Y	Y	Y	Y
**22**	N	N	Y	Y	Y	N	N	Y	Y	Y	N	Y	N
**23**	Y	Y	Y	Y	Y	Y	Y	Y	Y	Y	Y	Y	Y
**24**	Y	Y	Y	Y	Y	N	Y	Y	Y	Y	Y	Y	Y
**25**	Y	N	Y	N	N	N	Y	Y	N	N	N	Y	Y
**26**	Y	N	Y	Y	Y	N	Y	Y	N	N	N	Y	Y

Y= Reported; N=Not reported

**Item**	**Lawal et al, 2019**	**Scrimgeour et al, 2019**	**Tabernero et al, 2019**	**Bekoe et al, 2020**	**Jean-Baptiste et al, 2020**	**Hand et al, 2020**	**Husaini et al, 2020**	**Koech et al, 2020**	**Khurelbat et al, 2020**	**Schafermann et al, 2020**	**Sakuda et al, 2020**
**1**	N	N	Y	N	N	Y	N	Y	Y	N	N
**2**	Y	Y	Y	Y	N	Y	Y	Y	Y	Y	Y
**3**	N	N	Y	Y	Y	N	N	N	Y	N	N
**4**	N	N	Y	Y	N	N	N	N	Y	Y	N
**5**	N	N	N	Y	N	N	N	N	N	N	N
**6**	N	N	N	N	N	N	N	N	Y	N	N
**7**	N	N	Y	N	Y	N	N	N	N	Y	N
**8**	N	N	Y	Y	N	Y	Y	Y	Y	Y	Y
**9**	N	Y	Y	Y	N	Y	N	Y	Y	Y	Y
**10**	N	N	Y	N	N	N	N	N	Y	N	Y
**11**	N	N	Y	Y	N	N	Y	N	Y	Y	N
**12**	N	N	Y	N	N	Y	Y	N	N	N	N
**13**	N	N	Y	Y	N	N	N	N	N	N	N
**14**	N	Y	Y	Y	Y	N	N	N	Y	Y	Y
**15**	N	Y	Y	N	N	N	N	N	N	Y	N
**16**	N	N	N	N	N	N	N	N	N	Y	N
**17**	N	N	Y	Y	N	N	N	N	N	N	N
**18**	N	N	Y	N	N	N	N	N	N	N	N
**19**	N	Y	Y	N	N	N	N	N	Y	Y	Y
**20**	N	N	N	N	N	N	N	N	N	Y	Y
**21**	Y	Y	Y	Y	Y	Y	Y	Y	Y	Y	Y
**22**	N	Y	Y	Y	Y	Y	Y	Y	Y	Y	Y
**23**	Y	Y	Y	Y	Y	Y	Y	Y	Y	Y	Y
**24**	Y	Y	Y	Y	N	Y	Y	Y	Y	Y	Y
**25**	Y	Y	Y	N	Y	Y	Y	Y	Y	N	Y
**26**	Y	Y	Y	Y	Y	N	Y	Y	Y	Y	Y

Only the prevalence surveys published as original articles in scientific journals or following the Introduction/Methods/Results/Discussion or similar style and published as reports, MSc or PhD thesis, were appraised.

Y= Reported; N=Not reported
