# Supplementary material for: Substandard and falsified antibiotics: neglected drivers of antimicrobial resistance?
Source: BMJ Glob Health. 2022 Aug 18;7(8):e008587. doi: 10.1136/bmjgh-2022-008587 (PMC9394205; doi:10.1136/bmjgh-2022-008587)
Supplement: Supplementary data [file bmjgh-2022-008587supp011.pdf]

Substandard and falsified antibiotics: neglected drivers of antimicrobial resistance?

Supplementary file 11. Main characteristics of equivalence studies on the quality of antibiotics

Reference / country of collection	Active pharmaceutical ingredient (s) (API)	Brand name	Outlet	Reference standard	Test(s) performed and analytical technique for API content/Impurities	Failure rate n/N (%)
Mouton, 1979[1]<br>Netherlands	Cefadrine<br>Cefamandole<br>Cefalotin<br>Cefazolin<br>Cefotaxime<br>Cefoxitin<br>Cefuroxime	Unspecified	Manufacturer	Unspecified	API content - Microbiological assay	0/7 (0.0%)
Van der Bijl, 1988[2]<br>Unknown	Ampicillin	Famicillin, Penbritin, Petercillin	Unknown	Unspecified	API identification - HPLC-MS, microbiological assay, related substances	0/10 (0.0%)
Kibwage, 1991[3]<br>Kenya	Metronidazole	Flagyl, Unknown	Manufacturer<br>Wholesaler	BP	API content - UV-Spectroscopy, crushing strength, disintegration, dissolution, friability, mass uniformity	6/11 (54.5%)
Sakolchai, 1991[4]<br>Thailand	Metronidazole<br>Ampicillin<br>Co-trimoxazole	Unknown	Hospital/Health centre,<br>Private pharmacy	USP	API content - HPLC, dissolution, mass uniformity	6/14 (42.9%)
Antony, 1999[5]<br>Tanzania	Co-trimoxazole<br>Metronidazole	Bactrim, Flagyl, Unknown	Distributor<br>Pharmacy	BP, USP	API content – TLC and UV-Spectroscopy, disintegration, dissolution, friability	3/6 (50.0%)
Nightingale, 2000a[6]<br>Israel, Poland, Slovakia, Slovenia	Clarithromycin	Fromilid, Karin	Unknown	USP, Manufacturer's standard	API content - HPLC, dissolution, impurity	6/11 (54.6%)

Reference / country of collection	Active pharmaceutical ingredient (s) (API)	Brand name	Outlet	Reference standard	Test(s) performed and analytical technique for API content/Impurities	Failure rate n/N (%)
Nightingale, 2000b[7] Brazil, Chile, China, Colombia, Costa Rica, El Salvador, Guatemala, India, Indonesia, Pakistan, Peru, Turkey, Uruguay	Clarithromycin	Bicrolid, Clacine, Clambiotic, Clamicin, Clamycin, Claribac, Claricide, Clarimax, Clarimicin, Clarion, Clarithro-250, Claritrobac, Claritrol, Claritromicina, Claritromicina MK, Comtro, Crixan, Dicupal, Infex, Klarid, Klarolid, Klaromin, Klax, Limaixian, Macrol, Macrolien, Mus, Pre-Clar, Synclar	Unspecified	USP	API content – HPLC, dissolution, impurity – HPLC, visual inspection	34/40 (85.0%)
Mei, 2002[8] China	Cephradine	Unknown	Unknown	Chinese Pharmacopeia	API content – HPLC, dissolution	1/13 (7.7%)
Lambert, 2003[9] United Kingdom	Ceftriaxone	Axon, Broadced, Cefaxona, Cefaxone, Ceftrex, Ceftriaxon, Ceftriaxona GI, Ceftriaxona Sodium, Ceftriaxone Cef 3, Ceftriaxone, Cerixon, Desfin, Elpicef, Keptrix, KGE Ceftriaxone, LGP Ceftriaxone, Mercefex, Novocef, Oframax, Titan, Tpnakcoh, Triceft, Trixone, Unocef, Ventraxin, Zeftrox	Unknown	European Pharmacopoeia, USP	API content - HPLC, impurity - HPLC, pH, sterility	10/34 (29.4%)
Iqbal, 2004[10] Pakistan	Ofloxacin	Albact, Avocin, Bacivid, Ciof, Curitol, Eracin, Exact, Flovix, Floxy, Geoflox, Gyrasid, Gyrex,	Unknown	National Committee for Clinical Laboratory Standards	API content - In-vitro antibacterial susceptibility	15/34 (44.1%)

Reference / country of collection	Active pharmaceutical ingredient (s) (API)	Brand name	Outlet	Reference standard	Test(s) performed and analytical technique for API content/Impurities	Failure rate n/N (%)
		Kapcin, Korvid, Loxat, Oflamac, Oflobid, Oflobiotic, Ofloxin, Ofloquin, Oflox, Oftab, Oxil, Quinox, Rutix, Tabroxacin, Tariflox, Tarivid, Wiloxin				
Mei, 2004[11] China	Ceftriaxone	Unknown	Unregistered outlets	BP, European Pharmacopoeia, USP	API content - HPLC	0/17 (0%)
Nightingale, 2005[12] Argentina, Cyprus, Greece, Hong Kong, India, Indonesia, Malaysia, Mexico, Morocco, Pakistan, Palestine, Republic of Korea, Singapore, Slovenia, Spain, Uruguay	Clarithromycin	Adel, Berafen, Bicrolid, Binoclar, Clacin, Clamont, Clari, Clarion, Claripen, Claritek, Clarithro, Clarithromycin A, Claritromicina Alter, Claritromicina Bexal, Claritromicina, Geminis, Claritromicina Kern, Claromycin, Claros, Clarytas, Cleron, Crixan, E-Clar, Ezumycin, Fromilid, Hammi, Iset, Klaribact, Klaricare, Klermed, Neo-Klar, Pathocin, Ulcecure	Unspecified	USP	API content – HPLC, dissolution, impurity – HPLC, visual inspection	9/65 (13.9%)
Semde, 2005[13] Burkina Faso	Amoxicillin, Ampicillin, Metronidazole, Phenoxymethylpenicillin (Penicillin V), Sulfamethoxazole + Trimethoprim	Amoxicilline, Ampicilline, Bactrim Adultes, Clamoxyl, Co-trimoxazole, Flagyl, Metronidazole, Penicilline V, Starpen, Totapen	Wholesalers/ importer/ distributors	BP, European Pharmacopoeia, USP	API content – UV/Visible spectrophotometry, disintegration, dissolution, friability, hardness	0/20 (0%)
Adegbolagun, 2007[14] Nigeria	Ciprofloxacin	Unknown	Private pharmacy	BP, USP	API content – TLC, hardness, disintegration	4/10 (40.0%)

Reference / country of collection	Active pharmaceutical ingredient (s) (API)	Brand name	Outlet	Reference standard	Test(s) performed and analytical technique for API content/Impurities	Failure rate n/N (%)
Bamiro, 2007[15] Nigeria	Metronidazole	Unknown	Retail outlets - undefined	BP	API content – Titrimetry, disintegration, dissolution, friability, tablet hardness, mass uniformity	2/7 (28.6%)
Ibezim, 2008[16] Nigeria	Metronidazole	Unknown	Unregistered outlet, patent medicine vendor, hospital, private pharmacy	BP, USP	API content – UV/Visible spectrophotometry	9/10 (90.0%)
Mu'az, 2009[17] Nigeria	Ciprofloxacin	Unknown	Manufacturer	BP, USP	API content - LC, disintegration, dissolution, friability, hardness	0/7 (0%)
Nazir Mughal, 2009[18] Pakistan	Ciprofloxacin	Axcin, Ciproxin, Cyrocin, Mercip	Unknown	Unknown/Not specified.	API content - In-vitro antibacterial susceptibility	0/8 (0%)
Ngwuluka, 2009[19] Nigeria	Ciprofloxacin	Cipro-J, Ciprobon, Ciprogem, Ciproval, Ivacip, Vitapro	Private pharmacy	BP, USP	API content - UV-visible spectrophotometry, disintegration, dissolution, friability test, tablet hardness, mass uniformity	3/6 (50.0%)
Shahnaz, 2009[20] Pakistan	Cefixime	Unknown	Private pharmacy	BP, USP	API content – HPLC and microbiological assay, disintegration, dissolution, mass uniformity	0/6 (0%)
Wei, 2009[21] China	Cefteram	Unknown	Unknown	Unknown/Not specified	API content - HPLC	0/2 (0%)
Awofisayo, 2010[22] Nigeria	Ofloxacin	Asflovid, Drovid, Floxan, Floxavid, Gaxin, Oflomed, Tarivid, Traflox, Zanocin	Private pharmacy	USP	API content – UV-Spectroscopy, disintegration, dissolution, friability, tablet hardness, mass uniformity	9/9 (100.0%)
Nayak, 2010[23] India	Ciprofloxacin	Unknown	Private pharmacy	Indian Pharmacopeia, USP	API content – UV-spectrophotometry, disintegration, dissolution, friability, tablet hardness, mass uniformity	0/7 (0%)

Reference / country of collection	Active pharmaceutical ingredient (s) (API)	Brand name	Outlet	Reference standard	Test(s) performed and analytical technique for API content/Impurities	Failure rate n/N (%)
Silva, 2010[24] Colombia	Meropenem, Piperacillin-tazobactam	Unknown	Hospital/Health centres	Colombia National Formulary, United States Pharmacopeia	API content – microbiological assay	0/39 (0%)
Arshad, 2011[25] Pakistan	Gatifloxacin	Unknown	Private pharmacy	BP, USP	API content - HPLC-UV, disintegration, dissolution, tablet hardness, size variability, thickness, mass uniformity	0/5 (0%)
Bano, 2011[26] Pakistan	Levofloxacin	Unknown	Private pharmacy	BP, USP	API content - UV-visible spectrophotometry and In-vitro antibacterial susceptibility, disintegration, dissolution, friability, tablet hardness, thickness, mass uniformity	0/6 (0%)
Diaz, 2011[27] Colombia	Vancomycin	Unknown	Hospital/Health centres	USP	API content - In-vitro antibacterial susceptibility and microbiological assay	0/23 (0%)
Hailu, 2011[28] Ethiopia	Sulfamethoxazole-Trimethoprim	Bactrim, Bisepton, Cotreich, Cotri, Cotrimoxazole, Deprim	Manufacturer	BP, USP	API content – HPLC, disintegration, dissolution, friability, tablet hardness, thickness, mass uniformity	0/6 (0%)
Singal, 2011[29] India	Ciprofloxacin	Ciprobid	Private pharmacy	Indian Pharmacopeia	API content – HPLC and UV-Spectroscopy, API identification – IR spectroscopy, dissolution, mass uniformity	0/20 (0%)
Akpabio, 2011[30] Nigeria	Ciprofloxacin	Cefroden, Cipox, Ciprocare, Cipronol	Manufacturer	USP	API content – Titrimetry, disintegration, dissolution, friability, mass uniformity	1/4 (25.0%)
Mikre, 2011[31] Ethiopia	Amoxicillin	Unknown	Private pharmacy	BP, USP	API content- UV-visible spectrophotometry, API identification-TLC, dissolution, pH, mass uniformity, water content-Karl-Fischer titration	2/13 (15.4%)

Reference / country of collection	Active pharmaceutical ingredient (s) (API)	Brand name	Outlet	Reference standard	Test(s) performed and analytical technique for API content/Impurities	Failure rate n/N (%)
Musa, 2011[32] Nigeria	Metronidazole	Unknown	Hospital, Patent medicine vendor, Private pharmacy	BP	API content – unknown, dissolution, friability, tablet hardness, mass uniformity	10/15 (66.7%)
Omari, 2011[33] Jordan	Ciprofloxacin	Unknown	Manufacturer	US FDA, USP	API content - HPLC	0/2 (0%)
Singhal, 2011[34] India	Ciprofloxacin	Ciprobid, Ciproral	Private and public outlet - undefined	Indian Pharmacopeia	API content and identification – HPLC, content uniformity, mass uniformity	0/2 (0%)
Ashraful Islam, 2012[35] Bangladesh	Ciprofloxacin	Unknown	Private pharmacy	USP	API content – HPLC, disintegration, dissolution, friability, tablet hardness, mass uniformity	0/6 (0%)
Olanrewaju, 2012[36] Nigeria	Amoxicillin plus Clavulanic acid	Unknown	Private pharmacy	BP, USP	API content – HPLC, disintegration, dissolution, friability, tablet hardness, mass uniformity	3/6 (50.0%)
Onanuga, 2012[37] Nigeria	Ciprofloxacin, Gentamicin	Cenox, Cifran, Cipro-J, Ciprotab, Ciproxamed, Cyplox, Depmax, Elcees, Gentamicin Sulphate, Gentaven, Glogent, Paulio, Siprosan, Taman, Tivagenta, Unknown, Vitrapro	Combination of outlets, Private pharmacy	Unknown/Not specified	API content-in-vitro antibacterial susceptibility	11/17 (64.7%)
Nambiar, 2012[38] USA	Vancomycin	Unknown	Wholesaler/ Importer/ distributors	BP	API content and impurity – HPLC	0/6 (0%)
Hadwiger, 2012[39] United States of America	Vancomycin	Unknown	Manufacturer	BP, USP	API content - UHPLC, impurity - UV-MS	0/6 (0%)

**Reference / country of collection**	**Active pharmaceutical ingredient (s) (API)**	**Brand name**	**Outlet**	**Reference standard**	**Test(s) performed and analytical technique for API content/Impurities**	**Failure rate n/N (%)**
Qureshi, 2012[40] Pakistan	Ciprofloxacin	Unknown	Unknown	Unknown/Not specified	API content- HPLC	1/11 (9.1%)
Kahaliw, 2013[41] Ethiopia	Metronidazole	Amrizole, Camezol, Giardyl-125, Metazole, Metrogyl, Metrolag, Metronidazole	Private pharmacy	BP, Indian Pharmacopeia, USP	API content – HPLC, disintegration, dissolution, friability, tablet hardness, mass uniformity, sedimentation, viscometer	1/10 (10.0%)
Rahim, 2013[42] Pakistan	Cefadroxil	Unknown	Retail – undefined, Wholesaler	USP	API content – HPLC, disintegration, dissolution, tablet hardness, mass uniformity, tablet thickness	0/7 (0%)
Hussain, 2013[43] Pakistan	Ciprofloxacin	Unknown	Private pharmacy	USP	API content – UV spectroscopy, disintegration, dissolution, tablet hardness, mass uniformity	1/12 (8.3%)
Birhanu, 2013[44] Ethiopia	Erythromycin	Erycin, Erythromycin, Etocin, Rythro	Drug retail outlets – undefined	BP, USP	API content – unknown, disintegration, dissolution, tablet hardness, identity, mass uniformity	1/4 (25.0%)
Akhtar, 2013[45] Pakistan	Levofloxacin	Cravit, Farleo, Feelix, Lecord, Levodin, Levofin, Levoflox, Levosafe, Qevo	Unknown	BP, USP	Disintegration, dissolution, friability, tablet hardness, mass uniformity	8/9 (88.9%)
Kassaye, 2013[46] Ethiopia	Amoxicillin	Unknown	Unknown	USP	Dissolution	0/8 (0%)
Pais, 2013[47] India	Amoxicillin	Unknown	Government outlets	USP	API content-alkalimetry test and in-vitro antibacterial susceptibility, moisture content-drying oven method, pH, sedimentation test	4/12(33.3%)
Naveed, 2014[48] Pakistan	Doxycycline	Doxycycline, Doxyn, Vibramycin	Unknown	BP, USP	Disintegration, dissolution, mass uniformity	0/3 (0%)
Khamis, 2014[49] Gulf region	Metronidazole	Amrizole, Metrolags, Negazole, Nidazol	Private pharmacy	USP	API content – UV-spectroscopy, disintegration, friability, tablet hardness, mass uniformity	0/4 (0%)

Reference / country of collection	Active pharmaceutical ingredient (s) (API)	Brand name	Outlet	Reference standard	Test(s) performed and analytical technique for API content/Impurities	Failure rate n/N (%)
Al-Tahami, 2014[50] Yemen	Amoxicillin plus Clavulanic acid	Unknown	Private pharmacy	USP	API content – HPLC, pH	0/4 (0%)
Arnet, 2014[51] China, Myanmar	Ceftriaxone	Becef, Cefaxone, Cefin, Ceftriaxon, Incept, Oframax, Triacef	Wholesalers/ importer/ distributors	Manufacturer	API content – HPLC, API identification – NIR, impurity – HPLC, pH, physical appearance, sterility	8/8 (100.0%)
Aliyu, 2014[52] Nigeria	Metronidazole	Bonagyl, Emgyl, Jugyl, Lemetro, Loxagyl, Metrotab, Metrozam, Metrozo, Palagyl, Stargyl, Unigyl, Vamis	Private pharmacy	BP	API content – UV-spectroscopy, disintegration, dissolution, friability, tablet hardness, mass uniformity	7/12 (58.3%)
Humayoon, 2014[53] Pakistan	Doxycycline	Generic	Private pharmacy	BP	API content- UV-visible spectrophotometry, disintegration, dissolution, mass uniformity	0/6 (0%)
Idries, 2014[54] Sudan	Ceftriaxone	Unknown	Unknown	Unknown	API content - microbiological assay	1/2 (50.0%)
Fahmy, 2014[55] United Arab Emirates	Ciprofloxacin	Unknown	Private pharmacy	USP	Bioavailability, dissolution	0/6 (0%)
Fayyaz, 2014[56] Pakistan	Levofloxacin	Unknown	Unknown	USP	API content – HPLC, disintegration, dissolution, tablet hardness, mass uniformity	1/6 (16.7%)
Hamam, 2014[57] Sudan	Ciprofloxacin	Unknown	Unknown	USP	API content – HPLC, disintegration, friability, mass uniformity, tablet thickness	0/4 (0%)
Malele, 2014[58] Tanzania	Ciprofloxacin	Unknown	Combination of outlets	Unknown/Not specified	API content - In-vitro antibacterial susceptibility	4/9 (44.4%)
Nettey, 2014[59] Ghana	Amoxicillin-Clavulanic acid	Unknown	Private pharmacy	BP	API content – HPLC and in-vitro antibacterial susceptibility, disintegration, dissolution	29/38 (76.3%)

Reference / country of collection	Active pharmaceutical ingredient (s) (API)	Brand name	Outlet	Reference standard	Test(s) performed and analytical technique for API content/Impurities	Failure rate n/N (%)
Jaman, 2015[60] Bangladesh	Ciprofloxacin	Unknown	Unknown	USP	API content – UV spectroscopy, disintegration, dissolution, tablet hardness, mass uniformity, tablet thickness	0/5 (0%)
Aljohani, 2015[61] Egypt, India, Jordan, Morocco, United Kingdom, United States	Azithromycin	Unknown, Zithromax	Hospital/health centres, private pharmacy	US FDA, USP	API content - HPLC	5/19 (26.3%)
Bashir, 2015[62] Pakistan	Levofloxacin	Bexus, Cravit, Dynaquin, Effiflox, Floxolev, Glit, Lazer, L-cyn, Lecin, Leflox, Levo, Levocil, Levocure, Levoday, Levomerc, Levoscot, Levotar, Levotas, Levovis, Locus, Mclevo, Protektin, Qumic, Spectrix, Tavanic, Vizor, Voksec, Voxiquin, Xeflox	Unknown	National Committee for Clinical Laboratory Standards	In-vitro antibacterial susceptibility	0/29 (0%)
Bendari, 2015[63] Oman	Metronidazole	Flazole, Klont, Metrolag, Negazole, Nidazole, Suprazole	Unknown	USP	Disintegration, dissolution- UV-visible spectrophotometer, friability, tablet hardness, mass uniformity	2/6 (33.3%)
El Attug, 2015[64] Libya	Amoxicillin-clavulanic acid	Unknown	Unknown	USP	API content – LC and in-vitro antibacterial susceptibility, API ID – LC and FTIR, disintegration, dissolution, tablet hardness, tablet thickness, mass uniformity	0/5 (0%)
Alzomor, 2016[65] Yemen	Sulfamethoxazole plus trimethoprim	Amirtrim, Balkatrim, Batrim, Cotrix, Farcotrim, Omtran, Septram, Septrim, Shatrim, Sinotrim	Unregistered	BP, USP	API content – HPLC, disintegration, dissolution, friability, tablet hardness	2/10 (20.0%)

Reference / country of collection	Active pharmaceutical ingredient (s) (API)	Brand name	Outlet	Reference standard	Test(s) performed and analytical technique for API content/Impurities	Failure rate n/N (%)
Shraim, 2016[66] State of Palestine	Cefuroxime	Unknown	Unregistered	USP	API content – HPLC, dissolution, mass uniformity	0/3 (0%)
Idrees, 2016[67] Pakistan	Amoxicillin	Acamoxil, Amoxascot, Amoxicillin, Effimox, Glomox, HMC, Lapmox, Maxil, Medimox, Namoxil, Werrimox	Unknown	Unspecified	API content – HPLC and capillary electrophoresis	1/11 (9.1%)
Israr, 2016[68] Pakistan	Cefuroxime	Unknown	Manufacturer	USP	API content – HPLC, disintegration, dissolution, mass uniformity, thickness	0/4 (0%)
Osonwa, 2016[69] Nigeria	Ciprofloxacin	Unknown	Private pharmacy	BP	API content - UV-visible spectrophotometry, disintegration, dissolution, friability, Monsanto tablet tablet hardness, mass uniformity	1/15 (6.7%)
Pathak, 2016[70] India	Amoxicillin-clavulanic acid	Unknown	Private pharmacy	Unknown/Not specified	API content - In-vitro antibacterial susceptibility	2/6 (33.3%)
Adeniyi, 2017[71] Nigeria	Ciprofloxacin	Unknown	Private pharmacy	BP	API content - In-vitro antibacterial susceptibility, mass uniformity	0/5 (0%)
Al-Tabakha, 2017[72] United Arab Emirates	Amoxycillin plus Clavulanic acid	Unknown	Private pharmacy	USP	API content – HPLC, dissolution, friability, mass uniformity	0/5 (0%)
Uddin, 2017[73] Bangladesh	Ciprofloxacin	Unknown	Private pharmacy	BP, USP	API content – UV spectroscopy, disintegration, dissolution, friability, tablet hardness, mass uniformity	4/10 (40.0%)
Manani, 2017[74] Kenya	Clarithromycin	Unknown	Private pharmacy	USP	API content – HPLC, dissolution	0/16 (0%)

Reference / country of collection	Active pharmaceutical ingredient (s) (API)	Brand name	Outlet	Reference standard	Test(s) performed and analytical technique for API content/Impurities	Failure rate n/N (%)
Noor, 2017[75] Bangladesh	Metronidazole	Unknown	Private pharmacy	BP, USP	API content – UV/Visible spectrophotometry, disintegration, dissolution, friability, tablet hardness, mass uniformity, tablet thickness and diameter	0/9 (0%)
Gberindyer, 2017[76] Nigeria	Gentamicin	Unknown	Private pharmacy	USP	API content – HPLC-UV	0/14 (0%)
Fatima, 2017[77] Pakistan	Metronidazole	Unknown	Unknown	USP	Disintegration, dissolution, tablet hardness, mass uniformity, tablet thickness and diameter	2/3 (66.7%)
Alyahawi, 2018[78] Yemen	Ciprofloxacin	Unknown	Private pharmacy	BP, USP	API content - UV spectrophotometry, disintegration, dissolution, friability Roche friabilator, tablet hardness, mass uniformity	0/5 (0%)
Hasin, 2018[79] Bangladesh	Cefuroxime	Unknown	Private pharmacy	BP	API content – HPLC, disintegration, dissolution, friability, tablet hardness, mass uniformity, tablet thickness and diameter	0/3 (0%)
Gogoi, 2018[80] India	Ciprofloxacin	Unknown	Private pharmacy	Indian Pharmacopeia	API content and identification – HPLC, dissolution, mass uniformity	0/2 (0%)
Shaibu, 2018[81] Nigeria	Ciprofloxacin	Unknown	Private pharmacy	BP	API content – UV/Visible - Spectrophotometry, disintegration, friability, impurity, mass uniformity	3/20 (15.0%)
Thamer, 2018[82] Iraq	Cefotaxime	Cefotaxime LDP, Cefotaxime LG, Fortax, Kon-SEFATAX, Loraxime 1000	Private pharmacy	USP	API content – HPLC, API identification – NIR, impurity, pH, physical appearance	0/5 (0%)
Obarisiagbon, 2018[83] Nigeria	Erythromycin	Althrocin-S 500, Donythrocin-500, Enthrox-500, Erotab-500, Eryfast-500 Erythromycin, Erymycin,	Unknown	USP	Disintegration, dissolution, friability, tablet hardness, mass uniformity	5/12 (41.7%)

Reference / country of collection	Active pharmaceutical ingredient (s) (API)	Brand name	Outlet	Reference standard	Test(s) performed and analytical technique for API content/Impurities	Failure rate n/N (%)
		Erythrocin-500, Erythromycin, Erythromycin 500mg, Icethrocin, Labcin-500, Rycin Erythromycin, Zin zine				
Thambavita, 2018[84]<br>Sri Lanka	Metronidazole	Flagyl, Metrogyl	Private pharmacy	BP, USP	API content and Identification- UV-Vis Spectrophotometry disintegration, dissolution, tablet hardness, mass uniformity	0/2 (0%)
Ukwueze, 2018[85]<br>Nigeria	Azithromycin	Unknown	Private pharmacy	BP, USP	API content – UV/Visible spectrophotometry, disintegration, dissolution, friability, tablet hardness, mass uniformity	1/9 (11.1%)
Igboasoiyi, 2018[86]<br>Nigeria	Ciprofloxacin	Unknown	Private pharmacy	BP<br>USP	API content – Titrimetry and UV/Visible spectrophotometry, disintegration, dissolution, friability, tablet hardness, mass uniformity	3/10 (30.0%)
Rafiq, 2018[87]<br>Pakistan	Ofloxacin, Ciprofloxacin	Unknown	Government outlet, Private pharmacy	BP, USP	Disintegration, dissolution-UV/Visible spectrophotometer, friability, tablet hardness, thickness, mass uniformity	0/4 (0%)
Ogochukwu, 2018[88]<br>Nigeria	Azithromycin<br>Clarithromycin	Klabax, Zithromax, Unknown	Private pharmacy	USP	API content – microbiological assay and UV Spectrophotometry, disintegration, dissolution, friability, tablet hardness	0/9 (0%)
Bagbi, 2018[89]<br>Nigeria	Cefuroxime	Axacef, Donacef-500, Kadnat, Microcef-500, Sefzitil, Spizef, Zinnat	Private pharmacy	BP<br>USP	API content – UV/Visible spectrophotometry, disintegration, dissolution, friability, tablet hardness, mass uniformity	3/7 (42.9%)
Anah, 2019[90]<br>Nigeria	Ciprofloxacin	Unknown	Private pharmacy	BP<br>USP	API content – Titrimetry and UV spectrophotometry, disintegration, dissolution, friability, tablet hardness, mass uniformity	11/15 (73.3%)

**Reference / country of collection**	**Active pharmaceutical ingredient (s) (API)**	**Brand name**	**Outlet**	**Reference standard**	**Test(s) performed and analytical technique for API content/Impurities**	**Failure rate n/N (%)**
Avianto, 2019[91] Indonesia	Amoxicillin	Unknown	Hospital/Health centres	Indonesia Pharmacopoeia (Farmakope Indonesia)	API content - microbiological assay	0/5 (0%)
Pereira dos Anjos, 2019[92] Brazil	Amoxicillin, Azithromycin	Unknown	Government outlets - others	Unknown/Not specified	Impurities - LC-MS	1/2 (50.0%)
Rahman, 2019[93] Bangladesh	Ciprofloxacin	Unknown	Private pharmacy	BP, USP	API content - UV spectrophotometry, disintegration, dissolution, tablet hardness, mass uniformity	0/5 (0%)
Shah, 2019[94] Pakistan	Moxifloxacin	Unknown	Private pharmacy	BP, USP	API content – HPLC, disintegration, dissolution, friability, tablet hardness, mass uniformity	0/5 (0%)
Sharif, 2019[95] Pakistan	Azithromycin	Azomin, Ery-Pack, Unknown	Unknown	Unknown/Not specified	API content - Ouchterlony Double Immune Diffusion Technique	1/3 (33.3%)
Uwambajineza, 2019[96] Rwanda	Amoxicillin, Metronidazole	Unknown	Unknown	USP	Disintegration, dissolution, friability, HPLC-DAD, visual inspection, mass uniformity	1/14 (7.1%)
Hambisa, 2019[97] Ethiopia	Norfloxacin	Asnor, Gyrablock, Negaflox, Norbek, Norcin, Norfen, Norflox, Trizolin, Uriflox	Hospital/ Health centres, Private pharmacy	USP	API content – HPLC, dissolution, friability, tablet hardness, mass uniformity	2/9 (22.2%)
Agudelo, 2019[98] Columbia	Imipenem plus cilastatin	Inem, Tienam	Private pharmacy	USP	API content – HPLC-UV and microbiological assay	1/2 (50.0%)
Badulla, 2020[99] Yemen	Ciprofloxacin	Unknown	Unknown	BP	API content - Spectrophotometry-no detail	0/9 (0%)
Desta, 2020[100] Ethiopia	Ciprofloxacin	Unknown	Private pharmacy	USP	API content – HPLC, disintegration, dissolution - UV-visible spectrophotometer, mass uniformity	0/6 (0%)

**Reference / country of collection**	**Active pharmaceutical ingredient (s) (API)**	**Brand name**	**Outlet**	**Reference standard**	**Test(s) performed and analytical technique for API content/Impurities**	**Failure rate n/N (%)**
Hobeika, 2020[101] Lebanon	Amoxicllin, Amoxicillin-Clavulanic acid, Ciprofloxacin	Unknown	Private Pharmacy	USP	API content - HPLC	6/13 (46.2%)
Leficho, 2020[102] Ethiopia	Doxycycline	Doxy denk, Doxycad, Doxycap,Doxylagap, Doxyleb, Epadoxine, Medomycin, Miraclin, Remycin 100, Teradoxin	Private pharmacy	USP	API content and identification - HPLC, dissolution, friability, tablet hardness, visual inspection, mass uniformity	3/10 (30.0%)
Oli, 2020[103] Nigeria	Ciprofloxacin, Metronidazole	Unknown	Private pharmacy	BP	API content - UV-visible spectrophotometry, particulate matter, pH, sterility	12/16 (75.0%)
Rahman, 2020[104] Bangladesh	Metronidazole	Antipro, D-Metro, Filmet, Flagyl, Metryl	Combination of outlets	USP	API content - UV-visible spectrophotometry, disintegration, dissolution, friability, mass uniformity, size variability, tablet hardness, tablet thickness	0/5 (0%)
Tolentino-Hernandez, 2020[105] Mexico	Ciprofloxacin	Cipro XR, Ciproflox DM	Unknown	European Medicines Agency	API content - HPLC	0/2 (0%)

Note: FTIR - Fourier Transform Infrared Spectrometry, HPLC – High performance liquid chromatography, IR – infrared spectroscopy, LC – DAD - Liquid chromatography-diode array detection, NIR – near-infrared spectroscopy, TLC – Thin liquid chromatography, UHPLC – Ultra high-performance liquid chromatography, UV-MS – UV mass spectrometry

References

1 Mouton RP, Bongaerts GPA, Van Gestel M. Comparison of activity and beta-lactamase stability of cefotaxime with those of six other cephalosporins. *Antimicrob Agents Chemother* Published Online First: 1979. doi:10.1128/AAC.16.6.757

2 Van Der Bijl P, Seifart HI, Parkin DP, *et al.* Oligomeric substances in ampicillin preparations. A comparison of Penbritin, Famicillin and Petercillin. *South African Med J* 1988;**73**:453–5.

3 Kibwage IO, Thuranira J, Migosi D. Quality performance of metronidazole tablet products on the Kenyan market. *East Afr Med J* 1991;**68**:365–71.

4 Sakolchai S, Chaiyakum A, Wiyakrutta S, *et al.* A Survey on Qualities of Drugs Commercially Avilable in Thailand. 2010;**6**.

5 Antony PGR, Temu-Justin M, P.G.R. Antony MT-J. The Quality of Drugs Manufactured in Tanzania. *East Cent African J Pharm Sci* 1999;**2**:45–9.

6 Nightingale CH. A survey of the quality of generic clarithromycin products manufactured in Slovenia and Israel. *Adv Ther* 2000;**17**:167–78. doi:10.1007/BF02853159

7 Nightingale CH. A survey of the quality of generic clarithromycin products from 13 countries. *Clin Drug Investig* 2000;**19**:293–305. doi:10.2165/00044011-200019040-00007

8 Mei D, Zhao W, Qiang F, *et al.* Quality evaluation of different cefradine capsules. *Chinese J Antibiot* 2002;**1**.

9 Lambert PA, Conway BR. Pharmaceutical quality of ceftriaxone generic drug products compared with Rocephin®. *J Chemother* 2003;**15**:357–68. doi:10.1179/joc.2003.15.4.357

10 Iqbal M, Hakim ST, Hussain A, *et al.* Ofloxacin: Laboratory evaluation of the antibacterial activity of 34 brands representing 31 manufacturers available in Pakistan. *Pakistan J Med Sci* 2004;**20**:349–56.

11 Mei D, Du X, Li D. Pharmaceutical evaluation of different sterile ceftriaxone sodium products. *Chinese Pharm J* 2004;**39**:463–6.

12 Nightingale CH. A survey of the quality of generic clarithromycin products from 18 countries. *Clin Drug Investig* 2005;**25**:135–52.

13 Semdé R, Ouédraogo HW, Guissou IP, *et al.* Évaluation in vitro de la bio-équivalence de quelques formes génériques d'antibactériens. J. Pharm. Belg. 2005;**60**:51–5.

14 Adegbolagun OA, Olalade OA, Osumah SE. Comparative evaluation of the biopharmaceutical and chemical equivalence of some commercially available brands of ciprofloxacin hydrochloride tablets. *Trop J Pharm Res* 2007;**6**:737–45. doi:10.4314/tjpr.v6i3.14654

15 Bamiro OA, Odeniyi MA, Osonuga OA. Brand variations in the physicochemical properties of metronidazole tablets. *Nig Q J Hosp Med* 2007;**17**:22–5. doi:10.4314/nqjhm.v17i1.12536

16 Ibezim EC, Attama AA, Obitte NC, *et al.* In vitro prediction of in vivo bioavailability and bioequivalence of brands of metronidazole tablets in Eastern Nigerian drug market. *Sci Res Essay* 2008;**3**:552–8.

17 Mu'az J, Gazali LK, Sadiq GU, *et al.* Comparative in vitro evaluation of the pharmaceutical and chemical equivalence of multi-source generic

ciprofloxacin hydrochloride tablets around Maiduguri metropolitan area. *Niger J Pharm Sci* 2009;**8**:102–6.

18 Nazir Mughal MS, Asghar MT, Zia MA, *et al.* Comparison of the antibacterial activities of different brands of Ciprofloxacin. *Rev Científica UDO Agrícola* 2009;**9**:700–4.

19 Ngwuluka NC, Lawal K, Olorunfemi PO, *et al.* Post-market in vitro bioequivalence study of six brands of ciprofloxacin tablets/caplets in Jos, Nigeria. *Sci Res Essay* 2009;**4**:298–305.

20 Shahnaz G, Arshad HM, Shyum NSB, *et al.* Pharmaceutical Evaluation of Commercial Brands of Cefixime 400MG Capsules Marketed in Karachi (Pakistan). *J Pharm Res* 2009;**8**:98–104. doi:10.18579/jpcrkc/2009/8/2/79774

21 Wei F, Zhu R, Zhao W, *et al.* Pharmacokinetics and bioequivalence studies of cefteram pivoxil in healthy Chinese volunteers. *Eur J Drug Metab Pharmacokinet* 2009;**34**:157–62. doi:10.1007/BF03191167

22 Awofisayo SO, Awofisayo OA, Eyen N, *et al.* Comparative assessment of the quality control measurements of multisource ofloxacin tablets marketed in Nigeria. *Dissolution Technol* 2010;**17**:20–5. doi:10.14227/DT170210P20

23 Nayak AK, Pal D. Comparative in vitro bioequivalence analysis of some ciprofloxacin HCl generic tablets. *Int J Pharm Sci Res* 2010;**1**:51–7.

24 Silva E, Díaz JA, Arias MJ, *et al.* Comparative in vitro study of the antimicrobial activities of different commercial antibiotic products for intravenous administration. *BMC Clin Pharmacol* 2010;**10**:1–13. doi:10.1186/1472-6904-10-3

25 Arshad H, Shoaib M, GHAYAS S, *et al.* Pharmaceutical quality control studies on gatifloxacin 200 mg tablets available in the Pakistani market. *Lat Am J Pharm* 2011;**30**:1922–6.

26 Bano R, Gauhar S, Naqvi SBS, *et al.* Pharmaceutical evaluation of different brands of levofloxacin tablets (250 mg) available in local market of Karachi (Pakistan). *Int J Curr Pharm Res* 2011;**3**:15–22.http://www.ijcpr.org/

27 Diaz JA, Silva E, Arias MJ, *et al.* Comparative in vitro study of the antimicrobial activities of different commercial antibiotic products of vancomycin. *BMC Clin Pharmacol* 2011;**11**:1–10. doi:10.1186/1472-6904-11-9

28 Hailu GS, Gutema GB, Asefaw AA, *et al.* Comparative assessment of the physicochemical and in-vitro bioavailability equivalence of co-trimoxazole tablets marketed in Tigray, Ethiopia. *Int J Pharm Sci Res* 2011;**2**:3210–8.

29 Singal G, Nanda A, Kotwani A. A comparative evaluation of price and quality of some branded versus branded-generic medicines of the same manufacturer in India. *Indian J Pharmacol* 2011;**43**:131–6. doi:10.4103/0253-7613.77344

30 Akpabio E, Jackson C, Ugwu C, *et al.* Quality control and in vitro bioequivalence studies on four brands of ciprofloxacin tablets commonly sold in Uyo Metropolis, Nigeria. *J Chem Pharm Res* 2011;**3**:734–41.

31 Mikre W, Mekonnen N. Assessment on the quality and influence of tropical storage conditions on amoxicillin formulations marketed in Ethiopia. *East Cent African J Pharm Sci* 2011;**14**:23–32.

32 Musa H, Sule YZ, Gwarzo MS. Assessment of physicochemical properties of metronidazole tablets marketed in Zaria, Nigeria. *Int J Pharm Pharm Sci* 2011;**3**:27–9.

33 Omari DM, Johary D, Salem II, *et al.* Bioequivalence of two oral extended release formulations of ciprofloxacin tablets in healthy male volunteers under fed and fasting conditions. *J Bioequivalence Bioavailab* 2011;**3**:038–42. doi:10.4172/jbb.1000055

34 Singhal GL, Kotwani A, Nanda A. Jan Aushadhi stores in India and quality of medicines therein. *Int J Pharm Pharm Sci* 2011;**3**:204–7.

35 Ashraful Islam S., Dewan I, Shahriar M, *et al.* Validation and application of a simple HPLC method for the comparative in vitro dissolution study of some multisource ciprofloxacin tablets. *IJPI ' s J Anal Chem* 2012;**2**:1–12.

36 Olanrewaju OJ, Paul AC, Olusola AM. Quality assessment of amoxicillin-clavulanate potassium tablets in Lagos, Nigeria. *J Chem Pharm Res* 2012;**4**:5032–8.

37 Onanuga A, Eboh DD. Biological Activities of Ciprofloxacin tablets and Gentamicin injections commonly used in. *J Pharm Biomed Sci* 2012;**14**:1–6.

38 Nambiar S, Madurawe RD, Zuk SM, *et al.* Product quality of parenteral vancomycin products in the United States. *Antimicrob Agents Chemother* 2012;**56**:2819–23. doi:10.1128/AAC.05344-11

39 Hadwiger ME, Sommers CD, Mans DJ, *et al.* Quality assessment of U.S. marketplace vancomycin for injection products using high-resolution liquid chromatography-mass spectrometry and potency assays. *Antimicrob Agents Chemother* 2012;**56**:2824–30. doi:10.1128/AAC.00164-12

40 Qureshi MN, Rahman IU, Marwat GA. Comparative analysis of ciprofloxacin in different pharmaceutical products by high performance liquid chromatograph. *Sci Technol Dev* 2012;**31**:69–73.

41 Kahaliw W, Ashenef A. Comparative quality evaluation of some metronidazole tablets and metronidazole benzoate oral suspensions available in retail outlets of Addis Ababa, Ethiopia. *Int J Pharm Sci Res* 2013;**4**:1384–91.

42 N. Rahim, S. Baquir Shyum Naqvi, E. Iqbal, S. Nesar, U. Abdul Khaliq SKH. Investigation on Pharmaceutical Quality of Different Brands of Cefadroxil Monohydrate Available in Karachi, Pakistan. *Indo Am J Pharm Res* 2013;**3**:4557–84.

43 Hussain A, Hanif M, Shoaib M, *et al.* Comparative studies of ciprofloxacin 250 mg tablets available in pakistani market. *Lat Am J Pharm* 2013;**32**:484–9.http://www.latamjpharm.org/

44 Birhanu G, Kassa F, Hymete A, *et al.* Comparative in-vitro quality evaluation of erythromycin stearate tablets marketed in Addis Ababa, Ethiopia. *Int J Pharm Sci Res* 2013;**4**:378–85.http://www.ijpsr.com/

45 Akhtar MS, Malik A, Bashir S, *et al.* Pharmaceutical evaluation of different brands of Levofloxacin tablets (500MG) available in Pakistan. *Int J Curr Pharm Res* 2013;**5**:42–5.

46 Kassaye L, Genete G. Evaluation and comparison of in-vitro dissolution profiles for different brands of amoxicillin capsules. *African Heal Sci* 2013;**13**:369–75. doi:10.4314/ahs.v13i2.25

47 Pais KP, Panchmal GS, Shenoy R, *et al.* Comparison of medicine quality of the generic formulation of amoxicillin provided by the government of Karnataka with marketed brands - a public health perspective. *Int J Pharm Sci Rev Res* 2013;**23**:37–42.

48 Naveed S, Waheed N. Comparative study of three different brands of doxycycline capsules available in Karachi. *Open Access Libr J* 2014;**1**:1–7. doi:10.4236/oalib.1100458

49 Khamis ANR, Khan SA. Comparative in-vitro pharmaceutical evaluation of four brands of Metronidazole tablets marketed in gulf region. *Jordan J Pharm Sci* 2014;**7**:144–52. doi:10.12816/0027235

50 Al-Tahamani K. Quality and stability of co-amoxiclav products in Yemeni market. *J Chem Pharm Res* 2014;**6**:565–9.

51 Arnet I, Altermatt M, Roggo Y, *et al.* Pharmaceutical quality of eight generics of ceftriaxone preparation for injection in Eastern Asia. *J Chemother* 2015;**27**:337–42. doi:10.1179/1973947814Y.0000000208

52 Aliyu S. *Comparative bioequivalence studies of some brands of metronidazole tablets marketed in Zaria, Nigeria*. 2014.

53 Humayoon R, Razvi N, Anjum F, *et al.* Quality control testing and equivalence of Doxycycline Hyclate (100 mg) capsule brands under biowaiver conditions. *J Pharm Drug Deliv Res* 2014;**03**. doi:10.4172/2325-9604.1000124

54 Idries AM, Ibrahim KE. Microbiological Assay of Two Selected Products of Ceftriaxone Powder for Injection from Pharmaceuticals' Market in Sudan. *Sudan J Med Sci* 2014;**9**:251–8.

55 Fahmy S, Abu-Gharbieh E. In vitro dissolution and in vivo bioavailability of six brands of ciprofloxacin tablets administered in rabbits and their pharmacokinetic modeling. *Biomed Res Int* Published Online First: 2014. doi:10.1155/2014/590848

56 Fayyaz M, Yousuf RI, Shoaib MH, *et al.* Quality evaluation and in vitro interaction studies between levofloxacin 250mg and diclofenac sodium 50mg tablets. *Pak J Pharm Sci* 2015;**28**:119–28.

57 Hamam SBH. Comparative in vitro evaluation of generic ciprofloxacin hydrochloride tablets. *World J Pharm Pharm Sci* 2014;**3**:388–96.

58 Malele R., Mwambete K., Hassanali L. Antibacterial effects of nine brands of ciprofloxacin tablets available in Dar es Salaam. *East Cent African J Pharm Sci* 2014;**17**:10–7.

59 Nettey H, Allotey-Babington GL, Debrah P, *et al.* The quality and in vitro efficacy of amoxicillin/clavulanic acid formulations in the central region of Ghana. *Pharmacol Pharm* 2014;**5**:49–60. doi:10.4236/pp.2014.51009

60 Jaman M, Chowdhury AA, Rana AA, *et al.* In vitro evaluation of Ciprofloxacin Hydrochloride. *Bangladesh J Sci Ind Res* 2015;**50**:251–6. doi:10.3329/bjsir.v50i4.25833

61 Aljohani BA. *Pharmaceutical bioequivalence studies: Ensuring safety, effectiveness and high quality*. 2015.

62 Bashir S, Nasir SR, Usman F, *et al.* Comparative in-vitro evaluation of antibacterial activity of levofloxacin brands available in Pakistan. *Adv Life Sci* 2015;**2**:165–70.

63 Bendari A, Al-shehi B, Ahuja A. Comparison of pharmaceutical properties of different marketed brands of metronidazole tablets available in Oman. *Int J Pharm Arch* 2015;**4**:9–21.

64 El Attug MN, Ammar A, Tunsi S, *et al.* Pharmaceutical evaluation of an Augmentin brand manufactured in different countries. *Am J Pharm Pharmacol* 2015;**2**:35–44.

65 Alzomor AK, Al-Madhagi Y, Noman NM, *et al.* Quality Assessment of Ten Co-trimoxazole Brands as Tablet Compared to the Original Brand in

Yemeni Market. *Int J Biol Pharm Allied Sci* 2016;**5**:2746–56.

66 Shraim N, Zaid AN, Malkieh N, *et al.* Investigation of the interchangeability between cefuroxime axetil tablets marketed in Palestine. Is there a quality reason behind the price? *Palest Med Pharm J* 2016;**1**:7–14.

67 Idrees M. *Analysis of amoxicillin in counterfeit antibiotics from the Subcontinent and the Middle East*. Unplished MSc by Res. Thesis. 2009.

68 Israr F, Mahmood ZA, Hassan F, *et al.* Pharmaceutical evaluation of cefuroxime axetil tablets available in drug market of Pakistan. *Indian J Pharm Sci* 2016;**78**:17–26. doi:10.4103/0250-474X.180242

69 Osonwa UE, Abali SO, Chukwu KI, *et al.* Quality assessment of fifteen different brands of ciprofloxacin hydrochloride tablets marketed in Anambra State, Nigeria. *African J Pharm Res Dev* 2016;**8**:1–8.

70 Pathak P, Dawane J. In vitro comparison of generic and branded preparations of amoxicillin with potassium clavulanate. *J Clin Diagnostic Res* 2016;**10**:FC07–9. doi:10.7860/JCDR/2016/20009.8466

71 Adeniyi BA, Ayolabi CI, Ukonu EJ. Comparative antimicrobial activity of five brands of ciprofloxacin sold in Lagos state. *J Nat Sci Eng Technol* 2016;**15**:21–32.

72 Al-Tabakha MM, Fahelelbom KMS, Obaid DEE, *et al.* Quality attributes and in vitro bioequivalence of different brands of amoxicillin trihydrate tablets. *Pharmaceutics* 2017;**9**:1–11. doi:10.3390/pharmaceutics9020018

73 Uddin S, Mamun A Al, Hossain S, *et al.* In vitro quality evaluation of leading brands of ciprofloxacin tablets available in Bangladesh. *BMC Res Notes* 2017;**10**:1–9. doi:10.1186/s13104-017-2507-y

74 Manani RO, Abuga KO, Chepkwony HK. Pharmaceutical equivalence of clarithromycin oral dosage forms marketed in Nairobi County, Kenya. *Sci Pharm* 2017;**85**. doi:10.3390/scipharm85020020

75 Noor S, Salam FBA, Hima HN, *et al.* Comparative in-vitro quality evaluation of some brands of metronidazole tablet available in Bangladesh. *Int J Appl Res* 2017;**3**:753–8.

76 Gberindyer FA, Abatan MO, Wiesner L. Composition of gentamicin C components in gentamicin sulphate generics commonly used in small animal practice in Nigeria. *J Pharm Pharmacol* 2017;**5**:20–5. doi:10.17265/2328-2150/2017.01.005

77 Fatima S, Jamil S, Gauhar S, *et al.* In-vitro dissolution testing for therapeutic equivalence of metronidazole immediate release tablets available in Karachi, Pakistan. *Int J Pharm Sci Res* 2017;**8**:3133–7. doi:10.13040/ijpsr.0975-8232.8(7).3133-37

78 Alyahawi A, Alsaifi A. Quality control assessment of different brands of ciprofloxacin 500mg tablets in Yemen. *Univers J Pharm Res* 2018;**3**:29–33. doi:10.22270/ujpr.v3i4.180

79 Hasin F, Hossain M, Ghosh KK, *et al.* Pharmaceutical quality evaluation of cefuroxime axetil tablets available in drug market of Bangladesh. *World J Pharm Pharm Sci* 2018;**7**:1589–99. doi:10.20959/wjpps20181-10886

80 Gogoi B, Baishya H, Bordoloi R, *et al.* Quality control parameters of two different brands of ciprofloxacin tablets IP available commercially as per Indian Pharmacopoeia. *World J Pharm Res* 2018;**7**:1203–11. doi:10.20959/wjpr20182-10794

81 Shaibu AN, Audu AA. Physico-chemical and metal impurity assessment of some brands of ciprofloxacin hydrochloride tablets marketed in Kano Metropolis, Nigeria. *ChemSearch J* 2018;**9**:65–75.

82 Thamer HA, Haddad NS, Jawad AM. Analysis of drug contents of cefotaxime formulations available in the local market and the effect of storage conditions. *Int J Med Pharm Sci* 2018;**8**:11–8. doi:10.24247/ijmpsapr20183

83 Obarisiagbon AJ, Ogunlowo OP. Erythromycin Stearate Tablets: Quality Assurance Assessment. UK Essays. 2018.https://www.ukessays.com/essays/chemistry/erythromycin-stearate-tablets-quality-assurance-4970.php?vref=1 (accessed 24 May 2021).

84 Thambavita D, Fernando S, Galappatthy P, *et al.* Application of biowaiver methodology for a post-marketing study of generic and brand name metronidazole tablets. *Dissolution Technol* 2018;**25**:34–8. doi:10.14227/DT250218P34

85 Ukwueze S, Nwachukwu IP, Ezealisiji K. Ultraviolet spectrophotometric evaluation of different brand of azithromycin dihydrate tablets and capsules sold in Nigeria. *Eur J Pharm Med Res* 2018;**5**:80–4.

86 Igboasoiyi AC, Offor AC, Egeolu AP. Quality assessment of various brands of ciprofloxacin hydrochloride tablets sold in Uyo Metropolis. *Niger J Pharm Appl Sci Res* 2018;**7**:89–93.

87 Rafiq M, Khan TR, Iftikhar S, *et al.* Comparative quality control tests for different medicines supplied by medical store depot to health facilities and similar generics available in the market. *Indo Am J Pharm Sci* 2018;**5**:4914–9. doi:10.5281/ZENODO.1286148

88 Ogochukwu U, Christianah I, Marlene E, *et al.* Quality assessment of some brands of clarithromycin and azithromycin tablets using the concept of dissolution efficiency and similarity factor. *Int J Pharm Sci Res* 2018;**9**:5401–10. doi:10.13040/IJPSR.0975-8232.9(12).5401-10

89 Bagbi BM, Alagala MB, Ovwenahaye O, *et al.* Product quality assessment of brands of cefuroxime axetil tablets marketed in southern and western Nigeria. *Pharma Innov J* 2018;**7**:84–9.

90 Anah VU, Ebong AS, Charles GE, *et al.* Quantitative and qualitative analysis of some brands of ciprofloxacin tablets sold in Uyo metropolis, Akwa Ibom State, Nigeria. *Niger J Pharm Appl Sci Res* 2019;**8**:15–22.

91 Avianto P, Mahfudz M, Suharjono S, *et al.* In vitro equivalence of generic and branded amoxicillin tablet by microbiological assay method. *J Basic Clin Physiol Pharmacol* Published Online First: 2019. doi:10.1515/jbcpp-2019-0247

92 Pereira dos Anjos J, Sherratt S, Viana JD, *et al.* Detection of impurities in anti-infective generic drugs in Brazil by Liquid Chromatography-Mass Spectrometry. *J Bioeng Technol Appl to Heal* 2019;**2**:36–46. doi:10.34178/jbth.v2i2.55

93 Rahman M, Haque MA, Fahim NF. Comparative quality evaluation of different brands of ciprofloxacin tablets available in pharmaceutical market of Bangladesh. *Pharmacol Online* 2019;**1**:43–9.

94 Shah MR, Nasiri MI, Anwer S, *et al.* Pharmaceutical quality assessment of different brands of moxifloxacin 400 mg tablets available in Pakistan. *RADS J Pharm Pharm Sci* 2019;**7**:2–8.

95 Sharif H, Sharif S, Diwan H. Direct analysis of substandard antibiotics sensitivity pattern against common isolates of pathogens via modified Outerlony technique-A vitro study in Karachi. *Int J Curr Sci Multidiscip Res* 2019;**2**:126–34.

96 Uwambajineza T, Tuyishime F, Mugwiza E, *et al.* A survey of the quality of selected antibiotics in Kigali. Academia. 2019.https://www.academia.edu/38187916/A_SURVEY_OF_THE_QUALITY_ON_SELECTED_ANTIBIOTICS_IN_KIGALI_pdf (accessed 24 May 2021).

97 Hambisa S, Belew S, Suleman S. In vitro comparative quality assessment of different brands of norfloxacin tablets available in Jimma, Southwest Ethiopia. *Drug Des Devel Ther* 2019;**13**:1241–9. doi:10.2147/DDDT.S189524

98 Agudelo M, Rodriguez CA, Zuluaga AF, *et al.* Nontherapeutic equivalence of a generic product of imipenem-cilastatin is caused more by chemical instability of the active pharmaceutical ingredient (imipenem) than by its substandard amount of cilastatin. *PLoS One* 2019;**14**. doi:10.1371/journal.pone.0211096

99 Badulla WFS, Al-Omary YST, Ali KS. In vitro antimicrobial activity evaluation for different pharmaceutical dosage forms of ciprofloxacin Aden-Yemen. *Electron J Univ Aden Basic Appl Sci* 2020;**1**:93–9.

100 Desta HK, Teklehaimanot TT. In vitro quality evaluation of generic ciprofloxacin tablets available in community pharmacies of Dessie town, Northeast Ethiopia. *J Generic Med* 2020;**0**:1–8. doi:10.1177/1741134320921929

101 Hobeika E, Farhat J, Saab J, *et al.* Are antibiotics substandard in Lebanon? Quantification of active pharmaceutical ingredients between brand and generics of selected antibiotics. *BMC Pharmacol Toxicol* 2020;**21**:1–12. doi:10.1186/s40360-020-0390-y

102 Leficho AW, Lambore AH, Olbemo KS, *et al.* In-Vitro comparative quality assessment of different brands of doxycycline hyclate finished dosage forms: capsule and tablet in Jimma Town, South West Ethiopia. Reprints. 2020. doi:10.20944/preprints202004.0070.v1

103 Oli AN, Ibeabuchi MU, Enweani IB, *et al.* Pharmaceutical quality of selected metronidazole and ciprofloxacin infusions marketed in South Eastern Nigeria. *Drug Healthc Patient Saf* 2020;**12**:103–12. doi:10.2147/DHPS.S246050

104 Rahman M, Jahan FI, Fahim NF, *et al.* In vitro comparative quality evaluation of leading brands of metronidazole tablets available in Bangladesh. *Pharmacol Online* 2020;**2**:63–72.

105 Tolentino-Hernández SJ, Cruz-Antonio L, Torres-Roque I, *et al.* Bioequivalence of two oral ciprofloxacin extended-release formulations in healthy Mexican volunteers. *Lat Am J Clin Sci Med Technol* 2020;**2**:85–90. doi:10.34141/ljcs1055083
