# Supplementary material for: Substandard and falsified antibiotics: neglected drivers of antimicrobial resistance?
Source: BMJ Glob Health. 2022 Aug 18;7(8):e008587. doi: 10.1136/bmjgh-2022-008587 (PMC9394205; doi:10.1136/bmjgh-2022-008587)
Supplement: Supplementary data [file bmjgh-2022-008587supp012.pdf]

Substandard and falsified antibiotics: neglected drivers of antimicrobial resistance?

Supplementary file 12: Seizures/Recalls/Alerts/Case-reports of substandard and falsified antibiotics

Country	API/combination of APIs	Year	No. Batch/ Case	Quality test failure	Report Type	Ref
Cameroon	Metronidazole	Mar-2015	Unstated	Falsified-"Traffic de médicaments: 35 millions FCFA de médicaments contrefaits détruits dans la ville de Bafoussam […] Entre autres médicaments détruits, l'on a pu dénombrer du Métronidazole intraveineux Metlife 500mg/100ml […]"	Seizure	[1]
	Amoxicillin, Ampicillin	2016	Unstated	Falsified–no API	Recall/Warning/Alert	[2]
	Phenoxymethylpenicillin (Penicillin V)	Nov-2017	1	Falsified–wrong API (tablet contained paracetamol 50mg)	Recall/Warning/Alert	[3]
	Amoxicillin-Clavulanic acid	Mar-2018	1	Falsified–"[…] packaging appears to be a close imitation of the genuine product but GlaxoSmithKline confirmed they did not manufacture it."	Recall/Warning/Alert	[4]
Cambodia	Antibiotics-unspecified	Nov-2018	Unstated	SorF-"[…] illegal goods and substandard medication […] included […] antibiotics"	Seizure	[5]
Canada	Ampicillin, Doxycycline	Sept-2015	Unstated	Unregistered - seized at "Taste of Ukraine" in Burnaby	Seizure	[6]
	Amoxicillin, Ampicillin	May-2018	Unstated	Unregistered - seized from Gigi's Market, Ottawa	Seizure	[7]
Costa Rica	Ceftriaxone	Feb-2018	1	Falsified–contains cefazolin and a low concentration of unidentified contaminants.	Recall/Warning/Alert	[8]
Democratic Republic of Congo	Chloramphenicol, Sulfamethoxazole-Trimethoprim	2012	46	Falsified-"cotrimoxazole bought at illicit points of sale is the main etiology of fixed drug eruption"	Case report	[9]
	Amoxicillin, Ampicillin	Oct-2015	1	Falsified–no API	Recall/Warning/Alert	[10]
	Nalidixic acid	Jan-2016	Unstated	Falsified–no API ("30,000 false tablets")	Recall/Warning/Alert	[11]
	Cefixime	Jan-2018	2	Falsified–one batch contained no API and another batch contain 2.5% cefixime	Recall/Warning/Alert	[12]
Dominican Republic	Antibiotics-unspecified	2014	Unstated	Falsified-"Among the drugs seized there are […], antibiotics and […]"	Seizure	[13]
	Amoxicillin, Ampicillin	Oct-2015	3	Falsified-"List of the 22 falsified medicines: [….] Amoxicillin 500mg capsules, Amoxicillin 1000mg capsules, Ampicillin 1000mg capsules […]"	Recall/Warning/Alert	[14]
El Salvador	Ampicillin, Nitrofurantoin, Sulfamethoxazole-Trimethoprim	Jan-1991	1	Falsified – "[…] Scanprin tablets 400mg + 80mg score narrower and tablet is 1 mm higher than Scanpharm's; Furinamine s/c tablet 100mg weight approximately 560mg whereas Scanpharm's tablets weight approximately 460mg, colour brighter blue than Scanpharm's tablets; Ampiscan capsules 500mg capsule size 00 without snap fit whereas Scanpharm's capsules are size 0 E with snap fit."	Recall/Warning/Alert	[15]
France	Amoxicillin-Clavulanic Acid	Jan-2019	4	Substandard-labelling with incorrect administration instruction	Recall/Warning/Alert	[16]

Zabala GA, *et al. BMJ Global Health* 2022; 7:e008587. doi: 10.1136/bmjgh-2022-008587

Germany	Gentamicin	Jul-1997	1	Substandard-visible particulates	Recall/Warning/Alert	[17]
Guatemala	Gentamicin	Aug-2013	Unstated	Falsified–"A fake version of Gentamicina MK, injection solution 80mg […] under the name Gentamicina MIC"	Recall/Warning/Alert	[18]
Haiti	Ciprofloxacin	Apr-2001	1	SorF-"[…] unclear whether the ciprofloxacin he received in Haiti was substandard, his previous prompt response to quinolones alone suggested that it was."	Case report	[19]
India	Ampicillin, Cefalexin	2003	Unstated	Falsified-"[…] two antibiotics have been identified as Ampoxin-500 and Sporidex-500."	Seizure	[20]
	Amoxicillin-Clavulanic acid	Apr-2013	Unstated	Falsified–no API	Seizure	[21]
	Amoxicillin-dicloxacillin, ofloxacin	Jul-2014	Unstated	SorF–"Karnataka DC dept seizes 20 out-of-std quality drugs from pharmacies […] Staph-AC with amoxicillin and dicloxacillin […] Offmark-Oz containing ofloxacin and ornidazole […] Dorflox-OR (ofloxacin and ornidazole) […] Dibaymox which contains amoxicillin with dicloxacillin […]"	Seizure	[22]
	Ciprofloxacin	Nov-2014	13	SorF-contaminant	Case report	[23]
	Ciprofloxacin	Mar-2015	Unstated	SorF–API contents (not detailed)	Seizure	[24]
	Azithromycin	Jul-2015	1	SorF-visual inspection	Recall/Warning/Alert	[25]
	Cefixime	Jul-2015	1	SorF-API content and identification, dissolution, mass uniformity	Recall/Warning/Alert	[25]
	Erythromycin	Jul-2015	1	SorF-API content and identification, mass uniformity, water content	Recall/Warning/Alert	[25]
	Meropenem	Jul-2015	1	SorF-loss on drying	Recall/Warning/Alert	[25]
	Metronidazole	Jul-2015	1	SorF-dissolution	Recall/Warning/Alert	[25]
	Ceftazidime-Tazobactam	Oct-2015	1	SorF-impurity	Recall/Warning/Alert	[26]
	Clindamycin	May-2016	1	SorF-water content	Recall/Warning/Alert	[27]
	Norfloxacin	May-2016	1	SorF-dissolution	Recall/Warning/Alert	[27]
	Erythromycin	Aug-2016	2	SorF–API content (not detailed)	Seizure	[28]
	Amoxicillin, Cefixime, Ciprofloxacin, Ofloxacin	Dec-2016	Unstated	Falsified –"Koshia told TOI seized drugs included fake amoxicillin, ciprofloxacin, ofloxacin and even high-power antibiotics like cefixime."	Recall/Warning/Alert	[29]
	Gentamicin, Ofloxacin	Jun-2017	Unstated	SorF-"[…] found to be not of standard quality are […] Gentarid fo rIM/IV use which is gentamycin injection […] Lifobid-200 which are ofloxacin tablets […]"	Seizure	[30]
	Levofloxacin	Jul-2018	1	Substandard–low API, failed dissolution test	Case reports	[31]

	Amoxicillin-Clavulanic acid	Apr-2019	2	SorF-API content, API identification and particulate matter	Recall/Warning/Alert	[32]
	Cefpodoxime	Apr-2019	1	SorF-API content and disintegration	Recall/Warning/Alert	[32]
	Ciprofloxacin	Apr-2019	2	SorF-dissolution	Recall/Warning/Alert	[32]
	Gentamicin	Apr-2019	1	SorF-particulate matter	Recall/Warning/Alert	[32]
	Amikacin	Jun-2019	Unstated	Falsified-"[…] nearly 10,000 fake antibiotic vials of amikacin injection worth Rs 7 lakh were recovered in Agra."	Seizure	[33]
	Ceftriaxone	Nov-2019	1	SorF-dissolution	Recall/Warning/Alert	[34]
	Amoxicillin-Clavulanic acid	Dec-2019	1	SorF-API content, content uniformity	Recall/Warning/Alert	[35]
	Azithromycin, Cefadroxil, Cefuroxime, Ciprofloxacin, Erythromycin, Norfloxacin	Dec-2019	6	SorF-dissolution	Recall/Warning/Alert	[35]
	Azithromycin, Neomycin-Polymyxin, Tobramycin	Dec-2019	3	SorF-API content (not detailed)	Recall/Warning/Alert	[35]
	Cefixime	Dec-2019	1	SorF-API content, pH, water content	Recall/Warning/Alert	[35]
	Cloxacillin	Dec-2019	1	SorF-description, API identification	Recall/Warning/Alert	[35]
	Amoxicillin, Amoxicillin-Clavulanic acid, Clindamycin	Feb-2020	3	SorF-API content (not detailed)	Recall/Warning/Alert	[36]
	Erythromycin, Metronidazole	Feb-2020	2	SorF-dissolution	Recall/Warning/Alert	[36]
	Oxytetracycline	Feb-2020	1	SorF-water content	Recall/Warning/Alert	[36]
	Amoxicillin-Clavulanic acid	Apr-2020	1	Substandard-API content (not detailed)	Recall/Warning/Alert	[37]
	Cefotaxime	Apr-2020	1	Substandard-impurity	Recall/Warning/Alert	[37]
	Cefuroxime, Ofloxacin	Apr-2020	2	Substandard-API content	Recall/Warning/Alert	[37]
	Cefpodoxime, Chloramphenicol, Norfloxacin	Apr-2020	4	Substandard-dissolution	Recall/Warning/Alert	[37]
	Cefpodoxime, Ciprofloxacin	May-2020	2	SorF-dissolution	Recall/Warning/Alert	[38]
	Ofloxacin	May-2020	1	SorF-API identification	Recall/Warning/Alert	[38]
	Amoxicillin-Clavulanic acid	Jun-2020	1	SorF-API content (not detailed)	Recall/Warning/Alert	[39]
	Cefotaxime	Jun-2020	1	SorF-"water"	Recall/Warning/Alert	[39]
	Doxycycline	Jun-2020	2	SorF-API content, mass uniformity	Recall/Warning/Alert	[39]

	Nitrofurantoin	Jun-2020	1	SorF-dissolution	Recall/Warning/Alert	[39]
	Polymyxin B-Chloramphenicol-Dexamethasone	Jun-2020	1	SorF-API content (not detailed)	Recall/Warning/Alert	[39]
	Ciprofloxacin	Jul-2020	1	SorF-API content, dissolution	Recall/Warning/Alert	[40]
Indonesia	Antibiotics-unspecified	Mar-2009	Unstated	Falsified- "Counterfeit products included antibiotics…"	Seizure	[41]
Ireland	Gentamicin-Prednisolone eyedrops	Oct-2001	Unstated	Substandard-preservative efficacy	Recall/Warning/Alert	[42]
Kenya	Amoxicillin-Clavulanic acid	Aug-2019	1	Falsified–no API	Recall/Warning/Alert	[43]
Lao PDR	Tetracycline	Oct-2004	Unstated	SorF-API content	Case report	[44]
Mali	Amoxicillin	Mar-2014	Unstated	Falsified-"[…] district police of Bamako seized 192 cartons of counterfeit medicines. The boxes consisted […] amoxicillin."	Seizure	[45]
Nepal	Amoxicillin	Mar-2004	Unstated	Falsified–no API	Seizure	[46]
Niger	Amoxicillin	Oct-2014	1	Falsified–"[…] the following lots have been identified and confirmed as falsified Amoxycillin B.P 250mg […]"	Recall/Warning/Alert	[47]
Nigeria	Sulfamethoxazole-Trimethoprim	Feb-1993	Unstated	Falsified–wrong API	Recall/Warning/Alert	[48]
	Ampicillin-Cloxacillin, Lincomycin, Sulfamethoxazole-Trimethoprim	Jun-2010	Unstated	Falsified-"The fake drugs intercepted are […] lincomycin capsule 500, ampicillin/cloxacillin 500mg, trimethoprime 80mg/sulfametoxazol 400mg […]"	Recall/Warning/Alert	[49]
	Ampicillin-Cloxacillin, Chloramphenicol	Oct-2011	Unstated	Falsified-API identification	Recall/Warning/Alert	[50]
	Sulfamethoxazole-Trimethoprim	Oct-2011	Unstated	Unregistered-"Banned product"	Recall/Warning/Alert	[50]
	Amoxicillin-Clavulanic acid, Ampicillin-Cloxacillin, Bacitracin-Neomycin, Sulfamethoxazole-Trimethoprim	Jan-2014	Unstated	Falsified-"[…] the seized fake drugs […] Septrin suspension, Cicatrin powder, Ampiclox suspension, Augmentin syrup […]"	Seizure	[51]
Republic of the Philippines	Amoxicillin-Clavulanic acid, Cefdinir, Cefuroxime, Clindamycin, Gentamicin	Mar-2015	Unstated	Unregistered	Recall/Warning/Alert	[52]
	Clarithromycin	May-2015	Unstated	Falsified–no API, visual inspections	Recall/Warning/Alert	[53]
	Amoxicillin-Clavulanic acid	Jul-2015	Unstated	Substandard-Low API content	Recall/Warning/Alert	[54]

Tanzania	Amoxicillin, Ampicillin, Betamethasone-Clotrimazole-Gentamicin	Sep-2005	Unstated	Falsified-visible particulates, related substances, no API	Recall/Warning/Alert	[55]
	Betamethasone-Clotrimazole-Gentamicin	Sept-2005	Unstated	Falsified-low API, wrong API	Recall/Warning/Alert	[55]
Uganda	Tetracycline	Nov-2008	Unstated	Falsified–no API, wrong API (soya flour)	Seizure	[56]
	Ceftriaxone	Jan-2016	1	SorF-low API	Case report	[57]
	Amoxicillin-Clavulanic acid	Aug-2019	1	Falsified–no API	Recall/Warning/Alert	[43]
United Kingdom	Amoxicillin	Jul-2004	Unstated	SorF-low API	Recall/Warning/Alert	[58]
	Chloramphenicol eyedrops	Apr-2019	18	Substandard-incorrect PIL and labelling	Recall/Warning/Alert	[59]
United States of America	Tetracycline	Apr-1963	3	SorF-degraded "[…]patients suffering from Fanconi syndrome with suspicions that it was caused by a degradation product of tetracycline[…]"	Case report	[60]
	Clindamycin	Jun-2017	7	Substandard-microbial contamination	Recall/Warning/Alert	[61]
	Ceftriaxone	Jan-2019	42	Substandard-visual particulate in reconstituted vials	Recall/Warning/Alert	[62]
	Piperacillin-Tazobactam	Jul-2018	2	Substandard-presence of particulates identified as glass and silicone material	Recall/Warning/Alert	[63]
	Tetracycline	Apr-2020	8	SorF-dissolution	Recall/Warning/Alert	[64]
	Ceftazidime	May-2020	1	Substandard-stability	Recall/Warning/Alert	[65]
Unknown	Antibiotics-unspecified	Jun-2013	Unstated	Falsified-"[…] fake medicines seize during Pangea VI were antibiotics."	Seizure	[66]

REFERENCES

1 Donko P. Cameroon - Drug trafficking: 35 million FCFA of counterfeit drugs destroyed in the city of Bafoussam. Cameroon-Info.net. 2015.http://www.cameroon-info.net/article/cameroun-traffic-de-medicaments-35-millions-fcfa-de-medicaments-contrefaits-detruits-dans-la-ville-241500.html (accessed 7 Jun 2021).

2 Tchundju B. Sante : Amoxicillin 250 mg interdit de vente au Cameroun. Camersenat.info. 2016.http://www.camersenat.info/ (accessed 2 Sep 2017).

3 WHO. Medical Product Alert N° 4/2017: Falsified Penicillin V circulating in Cameroon. 2017.https://www.who.int/medicines/publications/drugalerts/drug_alert4-2017/en/ (accessed 1 Oct 2018).

4 WHO. Medical Product Alert N° 2/2018: Falsified 'Augmentin' circulating in Cameroon. 2018.https://www.who.int/medicines/publications/drugalerts/drug_alert2-2018/en/ (accessed 28 Sep 2019).

5 Sovuthy K. Authorities net more than 40 tonnes of counterfeit medicines - Khmer Times. Khmer Times. 2018.https://www.khmertimeskh.com/552535/authorities-net-more-than-40-tonnes-of-counterfeit-medicines/ (accessed 7 Jun 2021).

6 Health Canada. Three unauthorized health products labelled with prescription drug names seized at 'Taste of Ukraine' in Burnaby, B.C. - Recalls and safety alerts. Recalls Saf. alerts. 2015.https://healthycanadians.gc.ca/recall-alert-rappel-avis/hc-sc/2015/54918a-eng.php (accessed 1 Oct 2018).

7 Health Canada. Advisory - Unauthorized prescription antibiotic drugs seized from Gigi's Market in Ottawa, ON, may pose serious health risks. Cision Canada. 2018.https://www.newswire.ca/news-releases/advisory---unauthorized-prescription-antibiotic-drugs-seized-from-gigis-market-in-ottawa-on-may-pose-serious-health-risks-683078011.html (accessed 1 Oct 2018).

8 Ministerio de Salud Republica de Costa Rica. Deteccion De Medicamento Falso Rocephin 1 Gramo En Costa Rica. Alerta por Prod. en el Merc. 2018.https://registrelo.go.cr (accessed 1 Oct 2018).

9 Ognongo-Ibiaho AN, Atanda HL. Epidemiological study of fixed drug eruption in Pointe-Noire. *Int J Dermatol* 2012;**51**:30–1. doi:10.1111/j.1365-4632.2012.05561.x

10 Minilabs save lives. Minilabs save lives: Falsified antibiotics found in DRC. Minilabs save lives. 2015.https://web.facebook.com/minilab/photos/a.194864549665.125350.182507359665/10153694583429666/?_rdc=1&_rdr (accessed 4 Mar 2019).

11 Minilabs save lives. Falsified nalidixic acid with no active ingredient in DR-Congo. Facebook. 2016.https://www.facebook.com/minilab/posts/10154299278734666:0 (accessed 7 Jun 2021).

12 WHO. Medical Product Alert N° 1/2018: Falsified cefixime products circulating in the Democratic Republic of the Congo. 2018.https://www.who.int/medicines/publications/drugalerts/drug_alert1-2018/en/ (accessed 1 Oct 2018).

13 Anonymous. Health authorities seize counterfeit medicines in Santiago. Dominic. Today. 2014.https://dominicantoday.com/dr/local/2014/7/5/Health-authorities-seize-counterfeit-medicines-in-Santiago/ (accessed 7 Jun 2021).

14 Anonymous. The Dominican Republic Recalls 22 Batches Of Falsified Medicines. Int. Inst. Res. Against Counterfeit Med. 2015.http://www.iracm.com/en/2015/10/the-dominican-republic-recalls-22-batches-of-falsified-medicines/ (accessed 11 Jun 2018).

15 WHO. WHO Alert: Four counterfeit products recalled in El Salvador. 1991.https://www.who.int/

16 Sandoz Inc. Rappel de lots Amoxicilline Acide Clavulanique Sandoz Nourrissons et Enfants Document d ' information pour les patients. 2019.https://www.sandoz.fr/

17 WHO. Alert No. 60: Gentamicin ampoules defective product recall Helm Pharmaceuticals GMBH. 1997.https://www.who.int/

18 International Institute of Research Against Counterfeit Medicines (IRACM). Guatemala: Health Alert on Fake Medicines. Int. Inst. Res. Against Counterfeit Med. 2013.http://www.iracm.com/en/2013/08/guatemala-health-alert-on-fake-medicines/ (accessed 30 Oct 2018).

19 Reidenberg MM, Conner BA. Counterfeit and substandard drugs. *Clin Pharmacol Ther* 2001;**69**:189–93. doi:10.1067/mcp.2001.114672

20 India Express (New Delhi). Bad medicine: Sacks full of fake antibiotics seized. India Environ. Portal. 2003.http://www.indiaenvironmentportal.org.in/content/161624/bad-medicine-sacks-full-of-fake-antibiotics-seized/ (accessed 7 Jun 2021).

21 Hamid PA. Government hospitals in Indian-administered Kashmir supplied spurious antibiotics. Kashmir Newz. 2013.https://www.kashmirnewz.com/n000430.html (accessed 7 Jun 2021).

22 Bureau O. Karnataka DC dept seizes 20 not-of-std quality drugs from pharmacies, cos asked to withdraw stocks. Pharmabiz.com. 2014.http://www.pharmabiz.com/NewsDetails.aspx?aid=82889&sid=1 (accessed 7 Jun 2021).

23 Algar J. Rat poison in antibiotic pills may have killed at least 13 women in India. Tech Times. 2014.https://www.techtimes.com/articles/20314/20141116/shocking-rat-poison-chemical-in-antibiotic-pills-may-have-killed-at-least-13-indian-women-during-sterilization-drive.htm (accessed 12 Jun 2021).

24 Vijay N. Karnataka DC dept seizes 51 not-for-standard qlty drugs; cautions hospitals, public to not to use. Pharmabiz.com. 2015.

25 Central Drugs Standard Control Organization. Drug Alert list for month of July 2015. Notifications-Alerts.

2015.https://cdsco.gov.in/opencms/opencms/en/Notifications/Alerts/ (accessed 7 Jun 2021).

26 Central Drugs Standard Control Organization. Drug Alert list for month of October 2015. Notifications-Alerts. 2015.

27 Central Drugs Standard Control Organization. Drug Alert for the month of May 2016. Notifications-Alerts. 2016.

28 Vijay N. Karnataka DC seizes 11 NSQ drugs, cautions hospitals, public. Pharmabiz.com. 2016.

29 TNN. Fake antibiotics worth Rs 51 lakh seized. The Times of India. 2016.

30 Vijay N. Karnataka drugs control dept detects 16 NSQ drugs, alerts cos and pharmacies. Pharmabiz.com. 2017.http://www.pharmabiz.com/NewsDetails.aspx?aid=102744&sid=1 (accessed 8 Jun 2021).

31 Srinagar. Sale of substandard drugs continues unabated in Kashmir, alleges DAK. United News India. 2018.http://www.uniindia.com/sale-of-substandard-drugs-continues-unabated-in-kashmir-alleges-dak/states/news/1291562.html (accessed 8 Jun 2021).

32 Central Drugs Standard Control Organization. Drug Alert for the month of April 2019. Notifications-Alerts. 2019.

33 TNN. Nearly 10,000 fake antibiotic vials worth Rs 7 lakh recovered in Agra, 4 held. The Times of India. 2019.

34 Central Drugs Standard Control Organization. Drug Alert list for month of November 2019. Notifications-Alerts. 2019.https://cdsco.gov.in/opencms/opencms/en/Notifications/Alerts/ (accessed 8 Jun 2021).

35 Central Drugs Standard Control Organization. Drug Alert list for month of December 2019. Notifications-Alerts. 2019.

36 Central Drugs Standard Control Organization. Drug Alert for the month of February 2020. Notifications-Alerts. 2020.

37 Central Drugs Standard Control Organization. Drug Alert list for month of April 2020. Notifications-Alerts. 2020.

38 Central Drugs Standard Control Organization. Drug Alert for the month of May 2020. Notifications-Alerts. 2020.

39 Central Drugs Standard Control Organization. Drug Alert for the month of June 2020. Notifications-Alerts. 2020.

40 Central Drugs Standard Control Organization. Drug Alert for the month of July 2020. Notifications-Alerts. 2020.

41 Sagita D, Rachman A. Raids uncover counterfeit drugs. Jakarta Globe. 2009.

42 WHO Quality Assurance and Safety of Medicines Team. Alert No. 104: Rapid Alert Notification of a Quality Defect in Prednisolone- Gentamicin (Pred-G) Eye Drops. Rapid Alert Notif. a Qual. Defect. 2001.https://www.who.int/

43 WHO. Medical Product Alert N° 9/2019: Falsified Augmentin found in Uganda and Kenya. World Heal. Organ. 2019.https://www.who.int/medicines/publications/drugalerts/drug_alert-9-2019/en/ (accessed 3 Sep 2019).

44 Jähnke RWO. Counterfeit medicines and the GPHF-Minilab for rapid drug quality verification. *Fachthemen pharmind* 2004;**66**:1187–93.

45 International Institute of Research Against Counterfeit Medicines (IRACM). Mali: Seizure of counterfeit medicines. Media post. 2014.

46 Rai D. Which is real, and which is fake? Nepali Times. 2004.http://archive.nepalitimes.com/news.php?id=3765#.YL8guzb7RpR (accessed 8 Jun 2021).

47 WHO. Alert No. 132: Falsified Medicines west and central Africa. Drug Alert. 2014.https://www.who.int/medicines/publications/drugalerts/Alert_132_FalsifiedMedicinesWestandCentralAfricav2.pdf (accessed 19 Jun 2020).

48 WHO. Alert No. 33: Counterfeit Bactrim (Trimethoprim/sulfamethoxazole) syrup containing Valium (Diazepam). 1993.https://www.who.int/

49 Udoh F. Nigeria: NAFDAC Impounds consignments of fake drugs valued at N500 million. All Africa. 2010.

50 Yinka Oladoyinbo A. NAFDAC raises alarm over circulation of adulterated drugs in Ondo. Niger. Trib. 2011.

51 Adebowale S. Federal Operations Unit of Customs seizes fake drugs worth N12.5m. The Eagle Online. 2017.

52 Uy JR. FDA warns vs 20 unregistered drugs | Inquirer News. Inquirer.net. 2015.https://newsinfo.inquirer.net/680618/fda-warns-vs-20-unregistered-drugs (accessed 8 Jun 2021).

53 Solon A. Solon seeks probe on fake antibiotics. Phillipine Star. 2015.https://newsinfo.inquirer.net/739973/fda-warns-on-fake-antibiotics-for-kids-defective-anti-tb-drug-recalled (accessed 30 Jun 2017).

54 Jaymalin M. Substandard antibiotic recalled from market. Phillipine Star. 2015.http://www.philstar.com/headlines/2015/07/13/1476377/substandard-antibiotic-recalled-market (accessed 6 Jun 2017).

55 Ndomondo-Sigonda M. Counteracting Counterfeiting: Strategies for Improving the Integrity of the Medicinal Marketplace in Tanzania. Tanzania Food Drugs Auth. 2005.

56 Bagala A. 3 held over fake malaria drugs. Dly. Monit. 2008.

57 Nickerson JW, Attaran A, Westerberg BD, *et al.* Fatal Bacterial Meningitis Possibly Associated with Substandard Ceftriaxone — Uganda, 2013. *Morb Mortal Wkly Rep* 2016;**64**:1375–7. doi:10.15585/mmwr.mm6450a2

58 Anonymous. Amoxicillin injection recall. Pharm. J. 2004;**273**:105.

59 Medicines and Healthcare products Regulatory Agency. Class 4 Medicines Defect Information: Chloramphenicol 0.5% W/V Antibiotic Eye Drops (MDR 105-03/19). Drug Alert. 2019.https://www.gov.uk/drug-device-alerts/class-4-medicines-defect-information-chloramphenicol-0-5-w-v-antibiotic-eye-drops-mdr-105-03-19 (accessed 31 May 2019).

60 Frimpter GW, Timpanelli AE, Eisenmenger WJ, *et al.* Reversible 'Faconi syndrome' caused by degraded tetracycline. *JAMA - J Am Med Assoc* 1963;**184**:111–3. doi:10.1001/jama.1963.03700150065010

61 US Food & Drug Administration. Alvogen issues voluntary nationwide recall of clindamycin injection due to a potential for a lack of sterility assurance. Recalls, Mark. Withdrawals, Saf. Alerts. 2017.https://www.fda.gov/safety/recalls-market-withdrawals-safety-alerts/alvogen-issues-voluntary-nationwide-recall-clindamycin-injection-due-potential-lack-sterility (accessed 8 Jun 2021).

62 US FDA. Lupin Pharmaceuticals, Inc. Issues Voluntary Recall of Ceftriaxone for Injection USP, 250mg, 500mg, 1g and 2g | FDA. Recalls, Mark. Withdrawals, Saf. Alerts. 2019.https://www.fda.gov/safety/recalls-market-withdrawals-safety-alerts/lupin-pharmaceuticals-inc-issues-voluntary-recall-ceftriaxone-injection-usp-250mg-500mg-1g-and-2g (accessed 25 Jan 2019).

63 US FDA. AuroMedics Pharma LLC Issues Voluntary Nationwide Recall of Piperacillin and Tazobactam for Injection 3.375 grams per Vial, Due to Presence of Particulates Identified as Glass and Silicone Material | FDA. Recalls, Mark. Withdrawals, Saf. Alerts. 2018.https://www.fda.gov/safety/recalls-market-withdrawals-safety-alerts/auromedics-pharma-llc-issues-voluntary-nationwide-recall-piperacillin-and-tazobactam-injection-3375 (accessed 10 Aug 2018).

64 US Food & Drug Administration. Avet Pharmaceuticals Inc. issues voluntary nationwide recall of tetracycline HCl capsules USP, 250mg and 500mg due to failed dissolution specifications. Recalls, Mark. Withdrawals, Saf. Alerts. 2020.

65 US Food & Drug Administration. Ceftazidime injection by B. Braun: Recall - due to Out-of-Specification results for high molecular weight polymers. MedWatch. 2020.https://www.fda.gov/safety/recalls-market-withdrawals-safety-alerts/b-braun-medical-inc-issues-voluntary-nationwide-recall-one-1-lot-ceftazidime-injection-usp-and (accessed 8 Jun 2021).

66 Taylor P. Pangea VI swoop nets $41m-worth of fake medicines. Secur. Ind. 2013.
