# Supplementary material for: Substandard and falsified antibiotics: neglected drivers of antimicrobial resistance?
Source: BMJ Glob Health. 2022 Aug 18;7(8):e008587. doi: 10.1136/bmjgh-2022-008587 (PMC9394205; doi:10.1136/bmjgh-2022-008587)
Supplement: Supplementary data [file bmjgh-2022-008587supp013.pdf]

Substandard and falsified antibiotics: neglected drivers of antimicrobial resistance?

Supplementary file 13. Index of substandard and falsified antibiotic consumption.

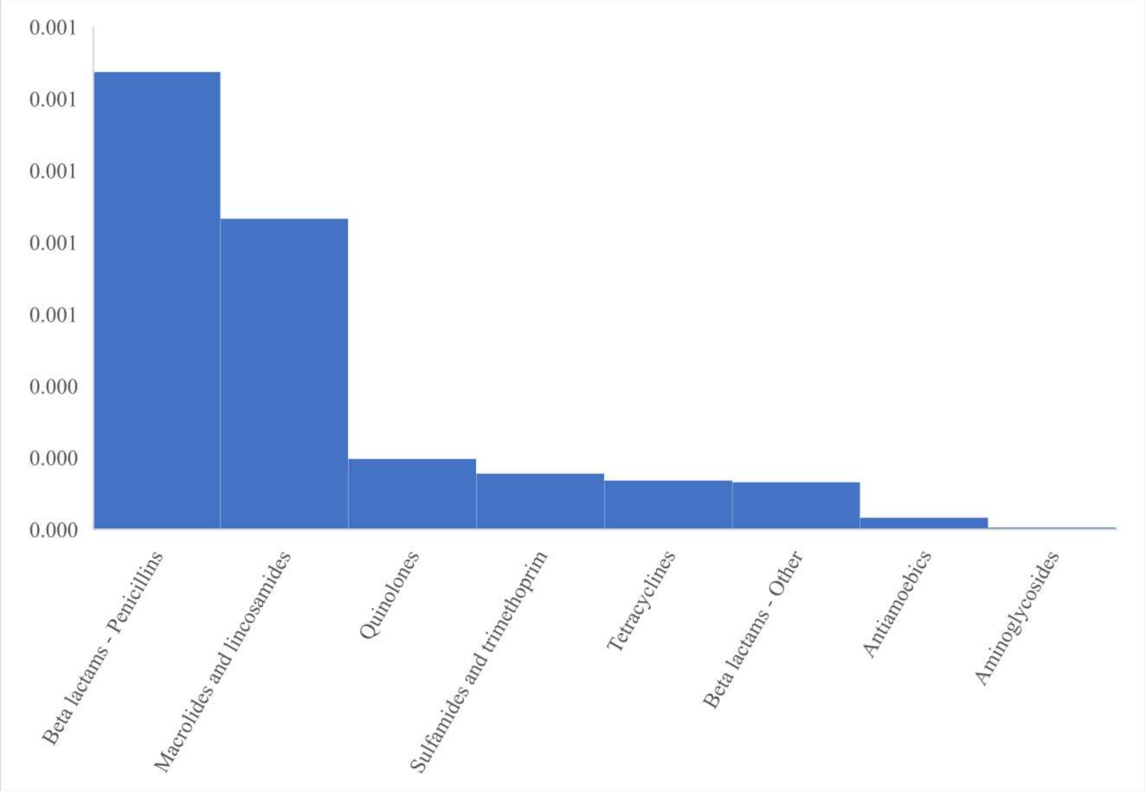

Global median SF antibiotic consumption measured in Defined Daily Doses per 1,000 inhabitants per day (Y axis), for different antibiotic groups. Calculated by multiplying global FF times global AMC

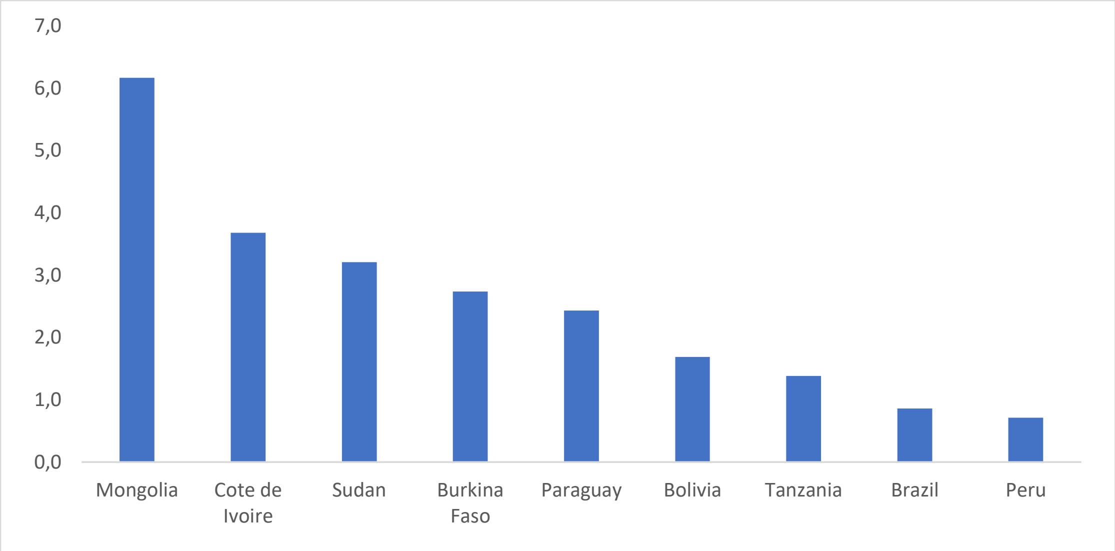

Median SF antibiotic AMC (in DDD per 1,000 inhabitants per day) (Y axis) for countries in which the WHO AMC report and our review had matching data.[1]

1 World Health Organization. WHO report on surveillance of antibiotic consumption: 2016-2018 early implementation. 2018;:1–127.https://www.who.int/medicines/areas/rational_use/oms-amr-amc-report-2016-2018/en/ (accessed 8 Sep 2019).
